# Structure and catalytic activity of the SAM-utilizing ribozyme SAMURI

**DOI:** 10.1038/s41589-024-01808-w

**Published:** 2025-01-08

**Authors:** Hsuan-Ai Chen, Takumi Okuda, Ann-Kathrin Lenz, Carolin P. M. Scheitl, Hermann Schindelin, Claudia Höbartner

**Affiliations:** 1 Institute of Organic Chemistry, Julius-Maximilians-Universität Würzburg, Würzburg, Germany; 2 Rudolf Virchow Center for Integrative and Translational Bioimaging, Julius-Maximilians-Universität Würzburg, Würzburg, Germany; 3 Center for Nanosystems Chemistry (CNC), Julius-Maximilians-Universität Würzburg, Würzburg, Germany

**Keywords:** RNA, X-ray crystallography

## Abstract

Ribozymes that catalyze site-specific RNA modification have recently gained increasing interest for their ability to mimic methyltransferase enzymes and for their application to install molecular tags. Recently, we reported SAMURI as a site-specific alkyltransferase ribozyme using *S*-adenosylmethionine (SAM) or a stabilized analog to transfer a methyl or propargyl group to *N*^3^ of an adenosine. Here, we report the crystal structures of SAMURI in the postcatalytic state. The structures reveal a three-helix junction with the catalytic core folded into four stacked layers, harboring the cofactor and the modified nucleotide. Detailed structure–activity analyses explain the cofactor scope and the structural basis for site selectivity. A structural comparison of SAMURI with SAM riboswitches sheds light on how the synthetic ribozyme overcomes the strategies of natural riboswitches to avoid self-methylation. Our results suggest that SAM and its analogs may serve as substrates for various RNA-catalyzed reactions, for which the corresponding ribozymes remain to be identified.

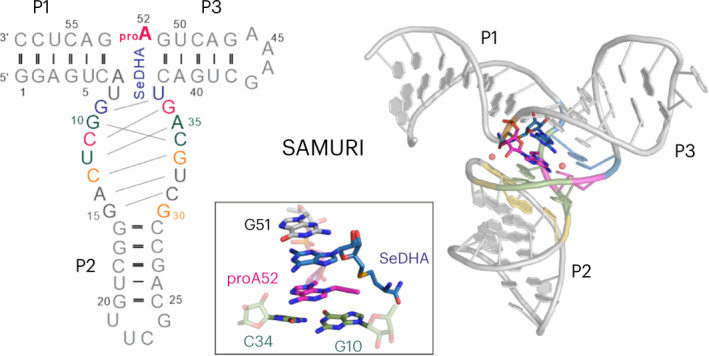

## Abstract (web summary)

Crystal structures of the ribozyme SAMURI reveal the alkyltransferase mechanism and show *N*^3^-modified adenosine and dealkylated cofactor in the active site, allowing for a comparison with riboswitches that bind *S*-adenosylmethionine but do not catalyze methyl transfer.

## Main

RNAs can fold into complex three-dimensional structures to enable versatile functions in nature and in the laboratory. Ribozymes are one class of structured RNAs that catalyze chemical reactions, mostly modifying themselves or other RNAs^1^. Natural nucleolytic ribozymes catalyze transformations of phosphodiester bonds (that is, cleavage or ligation events) mostly using metal ions or general acid–base catalysis^2^. After the accidental discovery of self-splicing ribozymes^3^, 11 classes were found in all kingdoms of life. New catalytic RNA motifs with often unknown biological functions were identified in sequence databases with the help of bioinformatic studies^4^ and many have been biochemically and structurally well characterized^5,6^. Examples of recently discovered ribozymes include the catalytic motif in Hovlinc^7^ RNA, hepatitis delta virus-like ribozymes involved in processing of bacteriophage transfer RNAs (tRNAs)^8^ and hydrolytic endonucleolytic ribozymes^9^ in bacterial genomes. Synthetic ribozymes that emerged from random RNA libraries by in vitro evolution focusing on phosphodiester formation can catalyze RNA ligation^10,11^, RNA polymerization^12–14^ and site-specific RNA labeling^15,16^. In addition, ribozymes have evolved to catalyze other chemical reactions^17^, including nucleoside synthesis^18^ and tRNA aminoacylation^19,20^, as well as reactions involving small molecules such as Diels–Alder, aldol and redox reactions^21–24^. In vitro selected ribozymes have also gained attention for site-specific RNA alkylation (for example, using iodoacetamide^25,26^ or epoxide derivatives^27,28^) for the attachment of fluorophores or biotin on RNA. Other ribozymes have evolved in vitro to use metabolites, such as thiamine^29^ or *S*-adenosylmethionine (SAM), which are prominent riboswitch ligands in nature.

Riboswitches represent a class of natural structured RNAs involved in the regulation of gene expression. Riboswitches contain ligand-binding aptamer domains that specifically bind small-molecule ligands such as enzyme cofactors and metabolites^30–32^. The most abundant riboswitch classes include thiamine pyrophosphate, SAM, adenosylcobalamine, tetrahydrofolate, flavin mononucleotide, amino acids, nucleotides and their derivatives. Riboswitches are considered ancient systems that may have their origin in the RNA world, where ribozymes and other functional RNAs served to store genetic information, guide metabolic states and promote chemical catalysis. It has been speculated that the RNA world exceeded the limited catalytic competence of contemporary ribozymes^33,34^. However, apart from the metabolite-activated nucleolytic glmS riboswitch ribozyme^35^, none of the currently known riboswitches has yet been shown to function as a ribozyme using a natural cofactor.

While structure-based studies of natural ribozymes and riboswitches have unveiled the catalytic mechanisms and ligand specificities, only limited structures of artificial ribozymes have been reported, where alkyltransferase ribozymes have recently been in the spotlight. For instance, a self-alkylating ribozyme was shown to adopt a preorganized helical structure accommodating its epoxide substrate in a dedicated binding pocket^28^. More recently, RNA-catalyzed RNA methylation was achieved with *O*^6^-methylguanine (m^6^G)^36^ or SAM^37^ as cofactors. The m^6^G-dependent ribozyme MTR1 generates 1-methyladenosine (m^1^A) and uses a protonated cytidine in the active site^38–41^. The postcatalytic state of MTR1 with bound guanine is structurally reminiscent of purine riboswitches^38^. The SAM-dependent ribozyme SMRZ requires Cu^2+^ for ligand binding and generation of 7-methylguanosine (m^7^G) in the active site^37^. Another example involves the preQ1 riboswitch, which was repurposed into a methyltransferase ribozyme by the use of the synthetic cofactor *O*^6^-methyl prequeuosine (m^6^preQ1)^42^. In addition, we recently reported the ribozyme SAMURI, which transfers a propargyl group from a stabilized SAM analog to the target RNA in a site-specific manner^43^. This small alkyne tag enables further functionalization of RNA (for example, through azide–alkyne click reactions). SAMURI uses a cell-permeable Se-methionine amide cofactor and maintains its catalytic activity under physiological Mg^2+^ concentrations, which enabled intracellular applications. In addition, SAMURI also showed methyltransferase activity with SAM as cofactor to generate 3-methyladenosine (m^3^A)-modified RNA in vitro but the structure and mechanism of SAMURI had yet to be determined.

Here, we report two crystal structures of SAMURI in the postcatalytic state, after reaction with either propargylic Se-2,6-diaminopurinribosyl-selenomethionineamide (ProSeDMA) or SAM. Both structures of the product complexes were solved at a resolution of 2.9 Å and feature the *N*^3^-propargylated or *N*^3^-methylated adenosine and the reacted cofactors Se-2,6-diaminopurinribosyl-selenohomocysteineamide (SeDHA) or *S*-adenosyl-l-homocysteine (SAH), respectively, in the core of a three-way helical junction (3HJ). Supported by in-line probing and mutagenesis data, the structures reveal how the target adenosine is juxtaposed with the cofactor for efficient transfer of the alkyl group. The comparison with natural riboswitch structures suggests how to overcome the strategies of natural RNAs to avoid self-methylation.

## Results

### Crystallization and overall structure of SAMURI

**Fig. 1:**
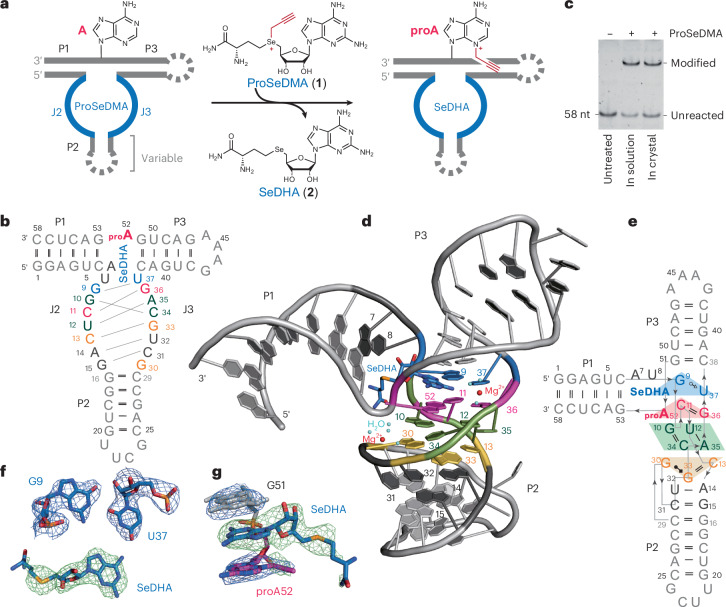
SAMURI-catalyzed propargyl transfer reaction and the overall structure. **a**, SAMURI transfers the propargyl group from ProSeDMA to *N*^3^ of adenosine and releases SeDHA. The reactive adenosine A52 is located in between P1 and P3. The dotted loops are present in the final crystallization construct. **b**, Sequence of the 58-nt crystallized RNA (R2; Supplementary Table 1) resulting in the postcatalytic structure with proA52 and SeDHA. **c**, Native PAGE analysis of a streptavidin-binding assay comparing the complex in solution and in the dissolved crystals, followed by copper-catalyzed click reaction with biotin azide, confirming the propargyl modification of the crystallized RNA (representative image from two independent experiments). **d**, Cartoon illustration of the three-dimensional structure of SAMURI. **e**, Schematic secondary-structure diagram with the four-layer architecture of the catalytic core highlighted in blue, red, green and yellow. **f**,**g**, Zoomed-in view of the cofactor-binding site in top (**f**) and side (**g**) views. The blue mesh represents the σA-weighted 2*F*_o_ − *F*_c_ map contoured at 1σ, while the position of SeDHA is confirmed by the *F*_o_ − *F*_c_ omit map (green, contoured at 3σ). Source data

The in vitro selected ribozyme SAMURI catalyzes the transfer of a propargyl group from the cofactor ProSeDMA (**1**) to *N*^3^ of the target adenosine (Fig. 1a), forming the alkylated nucleobase within the target RNA and releasing the uncharged cofactor SeDHA (**2**)^43^. The target adenosine is flanked by two Watson–Crick base-paired helices P1 and P3. The ribozyme core contains a central stem P2 that is connected to the binding arms P1 and P3 through the junctions J2 and J3, which are 9 and 8 nt long, respectively. The active site is formed in the center of the 3HJ architecture. The Watson–Crick base-paired stems P1, P2 and P3 are variable in length and sequence and can be blunt-ended, contain overhangs or capped with tetraloops (dotted lines in Fig. 1a). We screened several monomolecular, bimolecular and trimolecular RNA constructs for crystallization in the presence of ProSeDMA and found the best reproducibility and diffraction quality with a 58-nt monomolecular RNA (Fig. 1b). The SAMURI RNA constructs were prepared by in vitro transcription and the activity was confirmed by incubation with ProSeDMA, followed by click reaction with biotin azide and analysis by streptavidin binding on native PAGE (Fig. 1c). To evaluate the modification state in the crystal, crystals were harvested, washed and dissolved and the RNA was analyzed using the same assay. The similarity of the band pattern in the native gel from dissolved crystals and a control reaction in solution strongly suggests that the crystals contained propargylated RNA; hence, the resulting structure represents a postcatalytic state.

**Extended Data Fig. 1:**
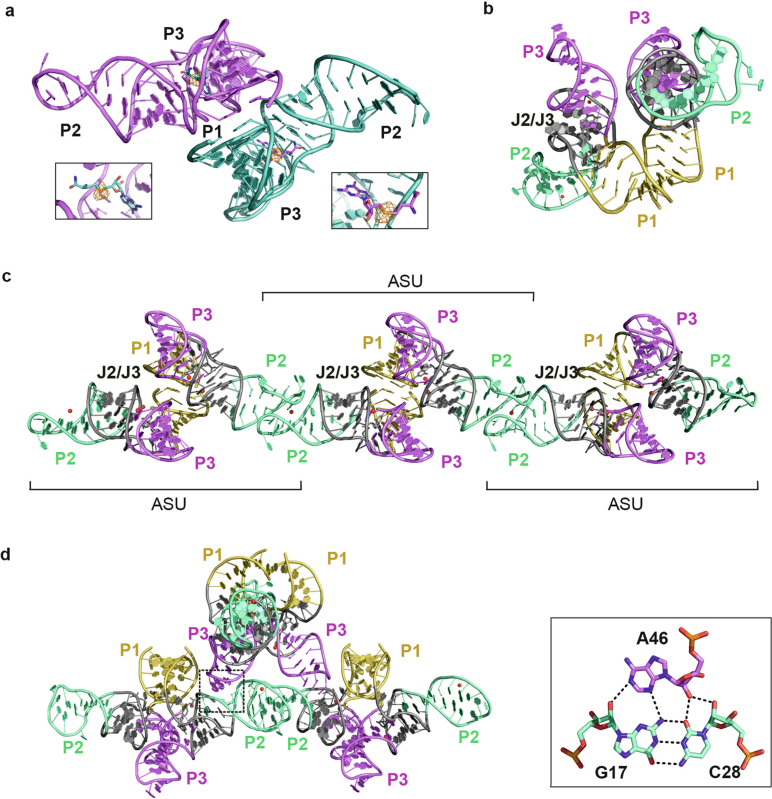
Crystal packing and contacts of SAMURI. **a**. Arrangement of the two SAMURI molecules present in the asymmetric unit. Orange meshes represent the anomalous difference map calculated with Phaser from data collected at the selenium K-edge at a contour level of 5σ. **b**. The P2, P3 and J2/J3 elements of SAMURI form coaxial helices, while the blunt ends of adjacent P1 stems are stacked onto each other to build up another semi-continuous duplex. **c**. The UUCG tetraloops of P2 from different asymmetric units contact each other via their major grooves to form an uncommon kissing loop interaction, which is stabilized by a hydrated magnesium ion (red sphere). This, together with the P1-P1 continuous helixes, builds up a compound zigzag helical structure. **d**. The GAAA loop of P3 (pink) contacts the minor groove of neighboring P2 stems (green), where two pairs of type I A minor motifs further stabilize the interaction (one example is shown in the box).

**Extended Data Fig. 2:**
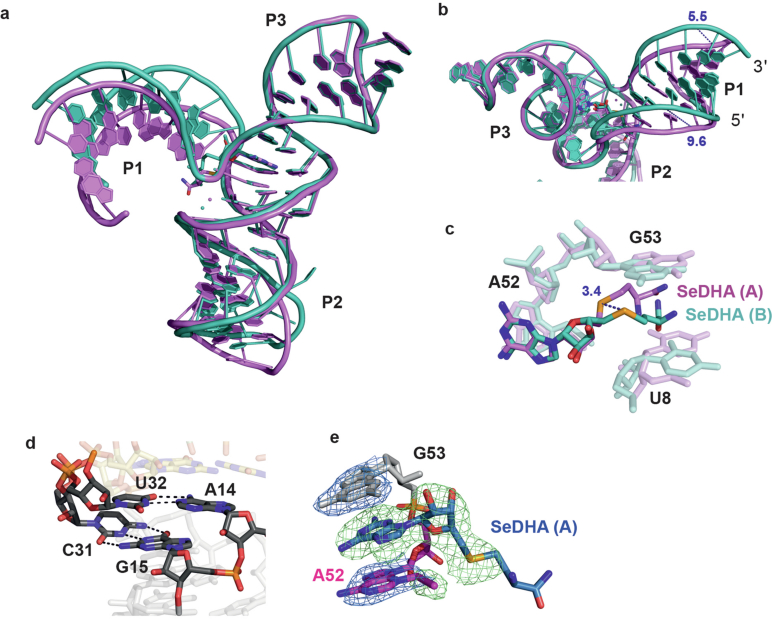
Comparison of the two molecules present in the asymmetric unit of SAMURI-ProSeDMA. **a**. Superimposition of the two molecules present in the asymmetric unit. **b**. The maximal distances between the molecules are located in the P1 stem with 9.6 and 5.5 Å at the 5′ and 3′ ends, respectively. This structural diversity correlates with the flexible location of the methionine moiety in the pocket (**c**). **d**. Zipper region connecting the bottom layer and P2. **e**. Electron density maps for the active site in the A molecule. Blue mesh represents the σA- weighted 2Fo-Fc map contoured at 1σ and positive difference density for the cofactor and the propargyl group is shown in green at a contour level of 3σ.

The crystals from the 58-nt RNA showed the best diffraction to a resolution of 2.9 Å. The crystals obtained from the ProSeDMA–SAMURI complex retained the Se-containing cofactor SeDHA, which facilitated phasing by single-wavelength anomalous dispersion (SAD). The hybrid substructure search (HySS) algorithm^44^ located two anomalous scatterers originating from the Se atoms of two copies of the molecule present in the asymmetric unit (Extended Data Fig. 1a). After calculation of experimental phases by Phaser^45^ and density modification by RESOLVE^46^, a continuous density map could be easily traced and fitted to the stem-loop sequences. Afterward, iterations of manual model building and refinement resulted in the final model presented in Fig. 1d. SAMURI crystals belong to space group *P*4_2_ and both copies present in the asymmetric unit feature bound SeDHA (Extended Data Fig. 1a). The two molecules within the asymmetric unit stack face to face through the blunt ends of P1 (Extended Data Fig. 1a,b) and the two tetraloops in P2 and P3 are involved in crystal contacts. The UUCG tetraloop on P2 is used as a dimerization interface through major groove contacts stabilized by a hydrated magnesium ion. The GAAA tetraloop on P3 forms a type I A-minor interaction with the minor groove of P2 from a neighboring molecule. These intermolecular stacking and H-bonding interactions build up the crystal lattice that resembles a compounded zigzag helical structure (Extended Data Fig. 1c). Superimposition of the two molecules within the asymmetric unit showed overall structural similarity (Extended Data Fig. 2a) as reflected in a root-mean-square deviation (r.m.s.d.) of 0.93 Å for 1,341 atom pairs and the fact that the catalytic core and P3 helix of both molecules are almost completely superimposable. In contrast, the P1 and P2 helices are somewhat displaced and the Se position of the cofactors is translocated by 3.4 Å (Extended Data Fig. 2b,c).

### Architecture of the active site of SAMURI

**Fig. 2:**
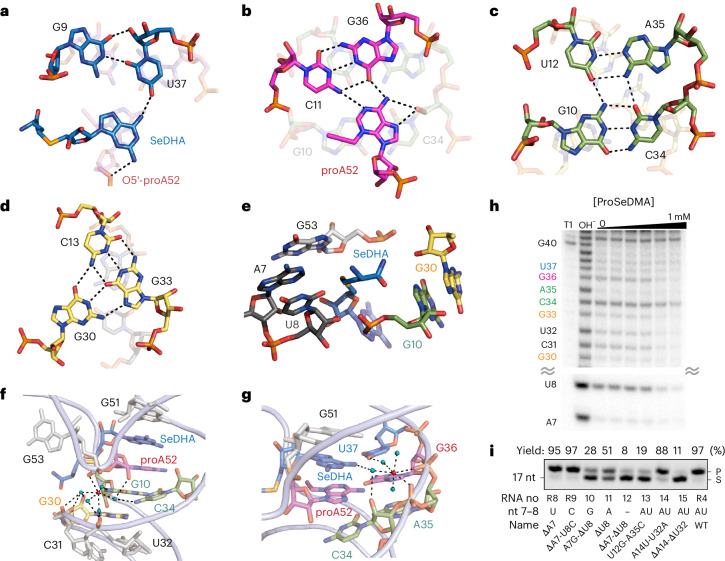
Structural details of SAMURI postcatalytic core. **a**, In the cofactor layer, SeDHA is contacted by G9 and U37 through Watson–Crick interactions with the sugar edge. **b**, In the reaction layer, the target site A52 forms a base triple with C11•G36. **c**, In the stabilization layer G10•C34 interacts with U12•A35. **d**, The bottom layer contains the base triple of C13•G33 and G30. **e**, Environment of the methionine moiety of SeDHA. **f**,**g**, The kink structure around the target site is stabilized by two hydrated magnesium ions. **h**, Representative excerpt of an in-line probing gel of SAMURI (pH 8.0, 20 °C, 18 h). **i**, The *trans*-activity analysis of SAMURI mutants. Reaction conditions: 1 µM Cy5-labeled substrate RNA R1, 10 µM SAMURI wild type (R4) or mutants (R8–R15) (Extended Data Fig. 4 and Supplementary Table 1), 10 µM ProSeDMA, 10 mM MgCl_2_, 37 °C, 4 h. In **h**,**i**, representative gel images are shown from three independent experiments. Source data

The 58-nt SAMURI construct folds into a 3HJ with the paired regions P1, P2 and P3 forming the expected three A-form helices, with P1 extending perpendicularly from the coaxial arrangement of P2 and P3. The nucleotides in J2 and J3 build up a four-layer structure that connects P2 and P3 through continuous *π*–*π* stacking. A schematic illustration of the tertiary structure with the interactions in the four layers (cofactor layer, reaction layer, stabilizing layer and bottom layer) of SAMURI is depicted in Fig. 1e. Electron density of the cofactor is clearly visible for the diaminopurine, ribose and Se atom (even from the first electron map generated), whereas the methionine amide tail is flexible and poorly resolved in both molecules of the asymmetric unit (Fig. 1f,g). The cofactor layer contains a base triple formed by the diaminopurine of SeDHA, G9 and U37. The noncanonical interaction features G9 in *syn* conformation, directing the Watson–Crick edge of G9 to interact with the sugar edge of U37 in the form of a *trans* G•U base pair. This arrangement positions *O4* of U37 to form a H-bond with the exocyclic *N*^6^ amino group of SeDHA (Fig. 2a). Another weak interaction to the diaminopurine is formed by the H-bond between the *N*^2^ amino group and *O*^5′^ of A52. In the reaction layer, the target adenosine forms a base triple with the C11•G36 Watson–Crick base pair. The Watson–Crick face of the *N*^3^-propargylated A52 (proA52) interacts with the canonical C11•G36 base pair from the major groove side (Fig. 2b). The Hoogsteen edge of A52 forms two additional H-bonds with the 2′-OH of C34. The stabilizing layer consists of two coplanar Watson–Crick base pairs (G10•C34 and U12•A35) that show additional inter-base-pair H-bonds between the major groove edge of U12•A35 and the minor groove side of G10•C34 (Fig. 2c). Another base triple (C13•G33 and G30) composes the bottom layer (Fig. 2d), in which G30 in the C2′-endo conformation docks into the major groove of C13•G33. Below the bottom layer, two canonical Watson–Crick base pairs (A14•U32 and G15•C33, zipper region) extend the P2 helix (Extended Data Fig. 2d). Only two nucleotides, A7 and U8 in J2, do not show any H-bonding interactions with other nucleotides; however, they are in the vicinity of the methionine tail of SeDHA (Fig. 2e).

An interesting feature of the SAMURI structure is the kink between the P1 and P3 helices (Figs. 1d and 2f), which splays apart A52 and G53, resulting in the intercalation of the target site A52 between the cofactor and G10. This orientation brings the reactive *N*^3^ of A52 and the Se center into proximity. Two hydrated magnesium ions stabilize the organization of the catalytic core (Fig. 2f,g). The kink in the phosphodiester backbone between A52 and G53 is stabilized by one hydrated magnesium ion that neutralizes the negative charges from the nearby phosphates of C31 and U32 and is coordinated to the nucleobase of G10 and C34 and the ribose of A52 and G30. The second magnesium ion is located on the nucleobase side of the cofactor, between the phosphate backbone of A35 and G36, and mediates the arrangement of the reaction layer and cofactor layer by H-bonding with *N*^6^ of SeDHA, *O*^2′^ of C34 and *O*^4^ of U37 through coordinated water molecules.

**Fig. 3:**
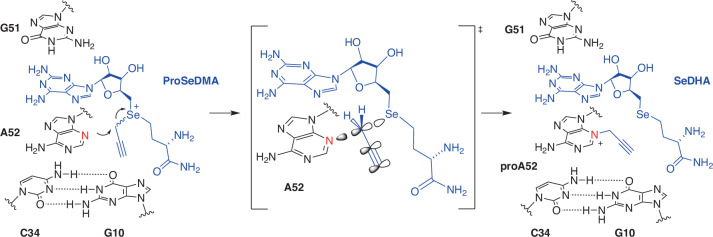
SAMURI-catalyzed propargyl transfer mechanism. The transpropargylation reaction proceeds through an SN2-like mechanism, featuring an in-line orientation of the nucleophile N3 (red) and the selenium leaving group.

The kink in the structure places the modification site in line with the Se center and supports the transfer of the propargyl group from ProSeDMA to A52. The reaction mechanism involves a direct nucleophilic displacement reaction following an S_N_2 like mechanism, likely without direct involvement of acid–base or metal ion catalysis. The proposed reaction mechanism is displayed in Fig. 3. The role of the magnesium ions is to stabilize the architecture of the active center (Fig. 2f,g), which facilitates desolvation of the minor groove nitrogen and in-line arrangement of the nucleophile and electrophile in close spatial proximity. The *π*–*π* stacking interaction of the triple bond with G10 likely also contributes to lowering the activation barrier. Consistent with this confined space in the active site, SAMURI was shown to transfer propargyl, allyl and methyl groups^43^ but it cannot accommodate larger alkyl groups. The transfer of a benzyl group failed (Extended Data Fig. 5), likely because of a steric clash with the ribose phosphate backbone of G10.

### Validation of the crystal structure by in vitro experiments

**Extended Data Fig. 3:**
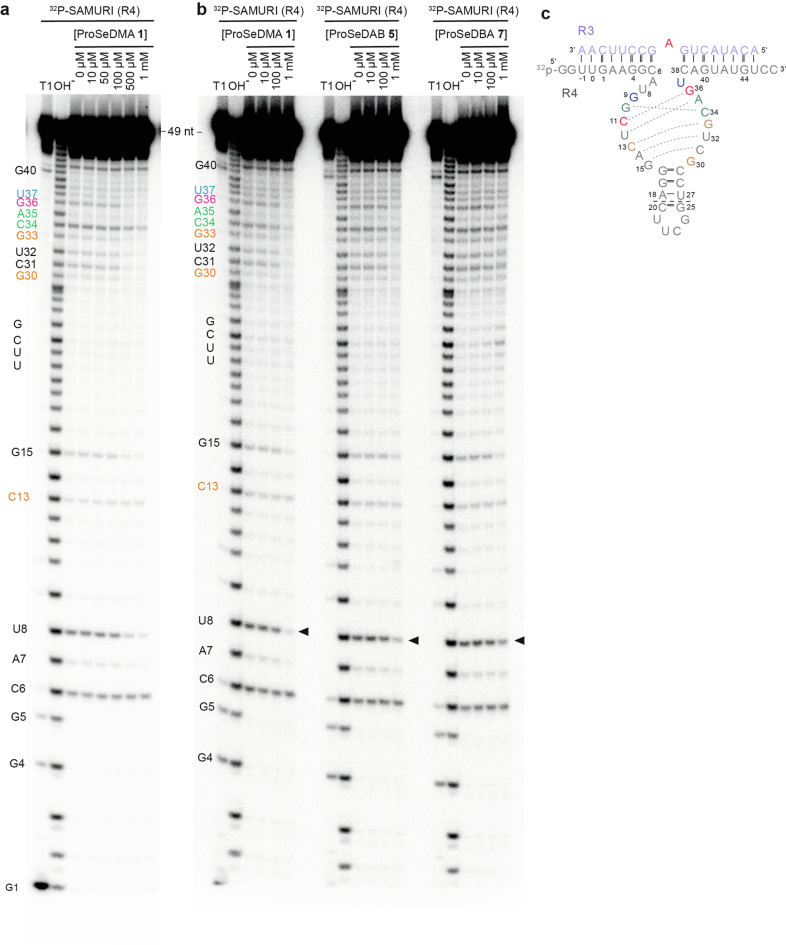
In-line probing of SAMURI. **a**. Full gel image of the in-line probing of SAMURI with increasing concentrations of ProSeDMA. **b**. In-line probings of SAMURI with ProSeDMA 1, ProSeDAB 5, and ProSeDBA 7. Different cofactors were calibrated via the absorption of diaminopurine at 280 nm (ε = 9894 L/mol•cm) to the same stock concentration. **c**. Bimolecular construct used in (a) and (b); numbering and color codes follow the scheme depicted in Fig. 1. All probing experiments were performed at pH 8, 20 °C for 16–18 h with two (b) and three (a) independent replicates. Source data

The cofactor is anchored in SAMURI through its diaminopurine nucleobase that forms H-bonds with the RNA and stacks in between G51 and proA52 (Figs. 1g and 2a), while the amino acid tail is more flexible, reflected by the weak electron density surrounding the methionine amide in the crystal structure (Figs. 1f and 2e). In-line probing experiments provided additional insights into potential interaction sites of the methionine moiety (Fig. 2h and Extended Data Fig. 3a). Increasing the concentration of ProSeDMA resulted in reduced intensities of the cleavage bands for several core nucleotides, including U8, G15, C31, U32, G33 and G36. Although the crystal structure does not give a strong hint for interactions of U8, the response in in-line probing experiments suggests that it may have a functional role. Thus, the contributions of U8 and other core nucleotides were further investigated using mutagenesis experiments.

**Extended Data Fig. 4:**
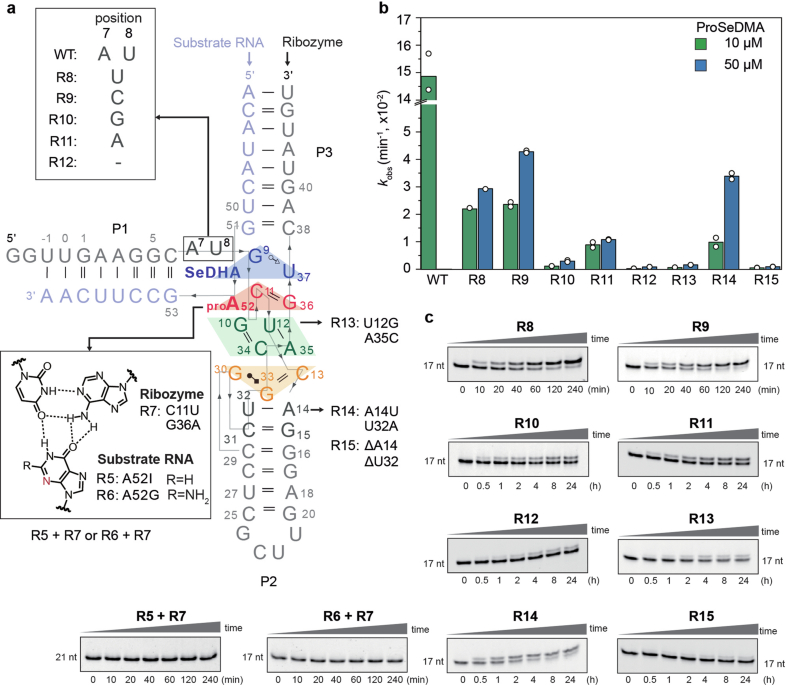
Kinetics of SAMURI mutants. **a**. RNA oligonucleotides used for in-trans kinetic assays in a schematic depiction according to the structure, with mutated nucleotides and base-pairs indicated. Sequence information for RNA substrate oligonucleotides R1, R5, R6, and ribozymes R7-R15 are provided in Supplementary Table 1. The box on the lower left details the suggested H-bonding interactions of the mutant ribozyme tested for alkylation of guanosine or inosine instead of adenosine. **b**. Summary of rate constants determined at two different ProSeDMA concentrations of 10 µM and 50 µM for the slower mutants. At least two independent replicates were performed. Exemplary gel images are shown in (**c**). Source data

Activity assays were performed in a bimolecular setup with a 3′ fluorescently labeled 17-nt substrate RNA (R1) hybridized to a 49-nt ribozyme strand (wild type, R4; mutants, R8–R15). This setup allowed the separation of modified and unmodified substrate RNAs by denaturing PAGE, enabling quantitative activity analysis of individual SAMURI mutants (Fig. 2i and Extended Data Fig. 4). The deletion of A7 was tolerated (R8 in Fig. 2i), suggesting that a single uridine as the linker nucleotide is sufficient for structure formation and the *trans*-alkylation reaction. Mutation to cytidine had a similar effect (R9), while the change to purines (R10 and R11) resulted in reduced activity, as reflected in both the observed rate constants (Extended Data Fig. 4) and the yields after 4 h of incubation time (Fig. 2i). These results (U ≈ C > A > G) suggested that pyrimidines are preferred and that the *O*^2^ H-bond acceptor assists the reaction, although it is not essential for the catalytic activity (because the purine mutants still maintained low activity). The double-deletion mutation (R12, ΔA7-ΔU8) resulted in severely reduced activity, likely because of the strong alteration of the distance or orientation between the P1 helix and the active center. Exchanging the Watson–Crick base pair U12•A35 to G12•C35 (R13) in the stabilizing layer was also strongly disfavored, which is consistent with the importance of the H-bonding interaction between the two base pairs U12•A35 and G10•C34 within the noncanonical quartet. Within the zipper region that extends P2, swapping of the canonical base pair A14•U32 to U14•A32 was well tolerated (R14), while deletion of this base pair strongly inhibited the reaction (R15). This result suggested that the length of the zipper is critical for enabling the base triple formation of G30 with C13•G33 in the bottom layer.

### Influence of cofactor structure on SAMURI activity

**Fig. 4:**
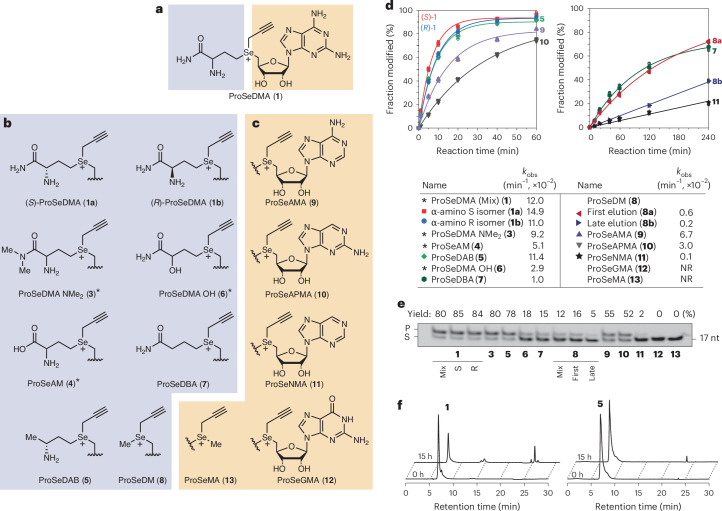
Structure–activity relationships of ProSeDMA derivatives. **a**, Chemical structure of ProSeDMA. Color code representing the methionine amide unit (blue) and nucleoside unit (orange). **b**, Mutations of the methionine unit. **c**, Mutations of the nucleoside unit. **d**, Kinetics of transpropargylation with ProSeDMA derivatives. The *k*_obs_ values were obtained under pseudo-first-order reaction conditions for 60 min. For ProSeDBA **7**, ProSeDM **8a/b**, ProSeNMA **11**, ProSeGMA **12** and ProSeMA **13**, incubation was extended to 4 h and a linear fit was applied for **8b** and **11**. Asterisks denote *k*_obs_ values reported in a previous study^43^. **e**, Gel image showing product formation with ProSeDMA derivatives. Reaction conditions: 1 µM Cy5-labeled substrate R1 (Supplementary Table 1), 10 µM SAMURI wild type (R4), 10 µM ProSeDMA derivatives, 10 mM MgCl_2_, 37 °C, 0.5 h. Representative gel image of three individual experiments. **f**, Stability comparison of ProSeDMA **1a** and ProSeDAB **5**. Here, 1 mM cofactor was incubated in 50 mM HEPES (pH 7.0), 120 mM KCl, 5 mM NaCl and 10 mM MgCl_2_ at 37 °C for 15 h and analyzed by RP-HPLC. In **d**, individual data points and the mean ± s.d. of three independent experiments are shown. Source data

**Extended Data Fig. 5:**
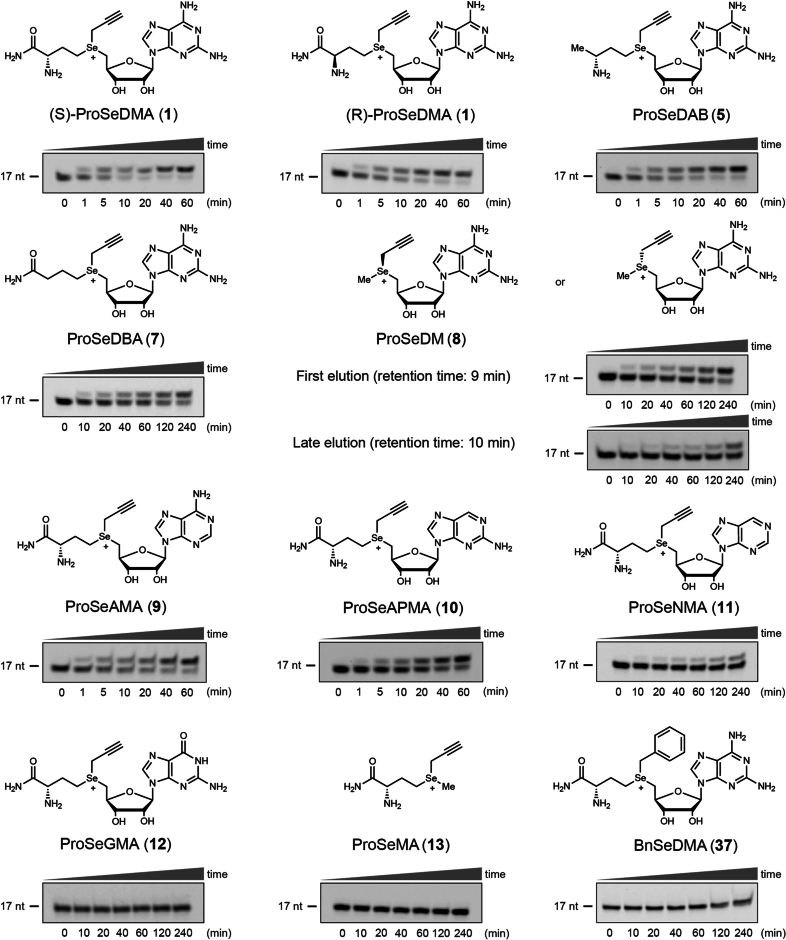
Influence of ProSeDMA variants on SAMURI activity. Gel images for kinetics graphs shown in Fig. 4d. Conditions: 10 pmol Cy5 labelled substrate RNA and 100 pmol ribozyme R4 in reaction buffer (50 mM HEPES, 120 mM KCl and 5 mM NaCl); after annealing (3 min at 95 °C, 10 min at 25 °C), 10 mM MgCl_2_ and 100 pmol respective cofactor were added (total reaction volume 10 μL). Incubation at 37 °C, 1 μL aliquots mixed with 4 μL of stop solution at each time point; *k*_obs_ determined from pseudo-first order curve fit; Representative gel images of three independent replicates for all cofactors. Source data

To further evaluate the contribution of individual functional groups for cofactor recognition, ten ProSeDMA analogs were synthesized and their activity was investigated by kinetic alkyltransferase experiments (Fig. 4 and Extended Data Fig. 5; details of the synthesis in the Supplementary Note). The extensive mutagenesis campaign of the cofactor structure (Fig. 4a) can be divided into two categories: modification of the methionine amide tail (Fig. 4b) and alteration of the nucleobase (Fig. 4c). First, we aimed at further insights into the interactions of the methionine amide moiety. We previously showed that alkylation of the terminal amide to dimethylamide in ProSeDMA-NMe_2_ (**3**) and the replacement of the amide by a carboxylic acid in ProSeAM (**4**) were tolerated^43^. To further test this observation and to assess the importance of the carbonyl group for recognition, the new cofactor ProSeDAB (**5**) was synthesized, which features a methyl group in place of the amide (that is, the α-amino butylamide is replaced by a secondary amine, the 2-(*R*)-amino-butyl group). Interestingly, ProSeDAB reacted almost as efficiently as ProSeDMA, yielding 90% propargylated RNA within 1 h. By contrast, alteration of the α-amino group by replacement with a α-hydroxyl group in ProSeDMA-OH (**6**)^43^ or deletion of the α-amino group in the butylamide in ProSeDBA (**7**) slowed the reaction drastically, yielding only ~20% propargylated RNA within 1 h. These results support the hypothesis that the α-amino group interacts with *O*^2^ of U8 to enhance the reaction. The comparison of the in-line probing patterns generated in the presence of **1**, **5** and **7** also supported this interpretation because the reduced cleavage response of U8 was observed with **1** and **5** but not with **7** (Extended Data Fig. 3b).

Next, we examined the effect of the configuration of the α-amino group on the reaction rate by comparing (*S*)-ProSeDMA and (*R*)-ProSeDMA. Both epimers showed comparably efficient reactions, indicating conformational flexibility in the recognition of the α-amino group. This result would be expected because the in vitro selection of SAMURI was performed with a mixture of diastereomers^43^, while the *S* epimer was used for crystallization. However, much to our surprise, the cofactor variant ProSeDM (**8**), in which the methionine unit was truncated to a methyl group, still served as propargyl donor. The reaction was about one order of magnitude slower than ProSeDMA and comparable to the variant **7** lacking the α-amino group. Interestingly, for the shortened analog **8**, the epimers at the Se center could be separated by reversed-phase high-performance liquid chromatography (RP-HPLC) and tested individually. The earlier-eluting isomer had an approximately threefold higher reaction rate than the later-eluting epimer. Although the absolute configuration of the epimers was not determined, these results suggested that, also for ProSeDMA, both *Se*-epimers may be accommodated in the active site, which would be consistent with the open space in the crystal structure and the lack of strong interactions involving the α-aminoamide side chain.

In our original design of ProSeDMA, the methionine amide was introduced to stabilize the cofactor compared to the earlier known ProSeAM with a carboxylic acid, which degraded quickly through an intramolecular cyclization reaction. Although the half-life of ProSeDMA was increased because the amide is less nucleophilic than the carboxylate, ProSeDMA still showed 40% degradation after 15 h at 37 °C under neutral pH conditions. However, because SAMURI does not benefit from the terminal amide, removing the nucleophile could further suppress the self-degradation and enhance cofactor stability. Indeed, the amino-butyl cofactor **5** showed very little decomposition (<10% after 15 h; Fig. 4f).

The second group of cofactors examined several atomic mutations of the nucleobase in ProSeDMA. The crystal structure revealed key interactions by H-bonding with the *N*^2^ and *N*^6^ amino groups of diaminopurine and by base stacking between G51 and A52. To evaluate the importance of each interaction, several derivatives with alternative nucleosides were synthesized and tested, including adenosine and 2-aminopurine riboside in ProSeAMA (**9**) and ProSeAPMA (**10**), each lacking one amino group compared to diaminopurine, nebularine (ProSeNMA (**11**), lacking both amino groups) and guanosine (ProSeGMA (**12**), different H-bond donor and acceptor pattern). Cofactors **9** and **10** showed slower kinetics than ProSeDMA but still yielded 70–80% propargylated RNA after 1 h. The kinetics were consistent with the H-bonds observed in the structure; adenosine (that is, 6-aminopurine) showed a twofold faster *k*_obs_ than 2-aminopurine. The importance of the amino groups was also reflected in the reactivity of the nebularine derivative **11**, which could only engage in *π*–*π* stacking but did not have exocyclic amino groups. Cofactor **11** showed only poor activity, reaching 20% product formation after 4 h. In contrast, with **12**, SAMURI activity was completely abolished. The cofactor **13**, in which the entire nucleoside was replaced by a methyl group, showed no activity as anticipated.

Overall, these cofactor mutagenesis experiments qualitatively reflected the combined effects of cofactor binding and influence on reaction rate. The results verified three major interaction elements between SAMURI and its cofactor: the nucleoside is the minimal recognition motif, where *π*–*π* stacking together with H-bond interactions secures the correct positioning that enables the *trans*-propargylation reaction, which is accelerated by installation of the 2-amino-butyl side chain that interacts with U8.

### Comparison of SAMURI structure with natural SAM riboswitches

**Fig. 5:**
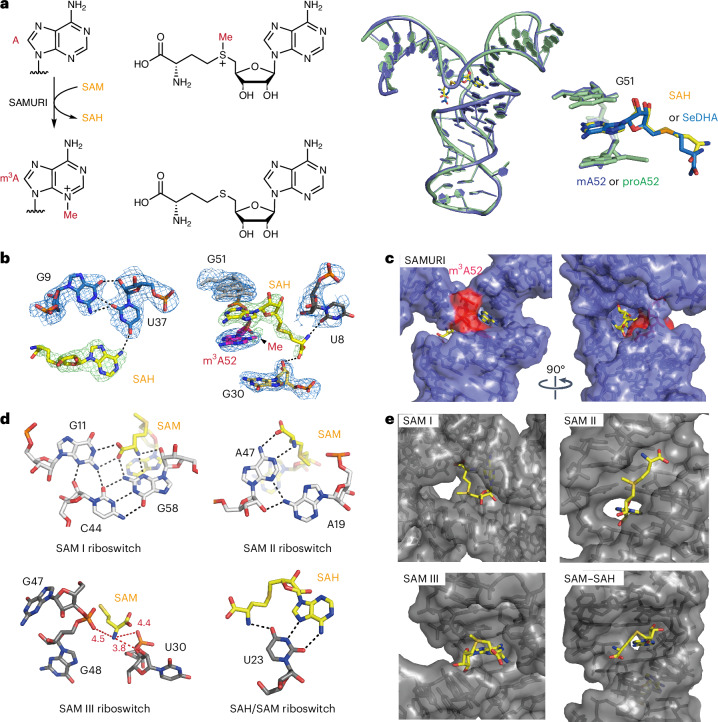
Structural comparison of SAM-using SAMURI and natural SAM riboswitches. **a**, The SAMURI-catalyzed methylation of A52 and the alignment of SAM-reacted SAMURI (blue) and ProSeDMA-reacted SAMURI (green). Overall structure and excerpt of the modified adenosine and reacted cofactor are shown on the right. **b**, Detailed structure of SAH-bound and m^3^A-containing SAMURI with the electron density map from front and top views. The σA-weighted 2*F*_o_ − *F*_c_ map contoured at 1σ is presented as a blue mesh, while the position of SAH and the methyl group is confirmed by the *F*_o_ − *F*_c_ omit map (green, contoured at 3σ). **c**, SAM-binding pocket of SAMURI shown in surface representation (blue) with SAH (yellow) and methylated A52 (red). **d**,**e**, SAM binding by natural riboswitches. Characteristic interactions between the methionine tail and the SAM-binding pocket of *Thermoanaerobacter tengcongensis* SAM I riboswitch (Protein Data bank (PDB) 2GIS), *metX* SAM II riboswitch (PDB 2QWY), *Enterococcus faecalis* SAM III riboswitch (PDB 3E5C) and the SAM–SAH riboswitch (PDB 6YL5).

**Extended Data Fig. 6:**
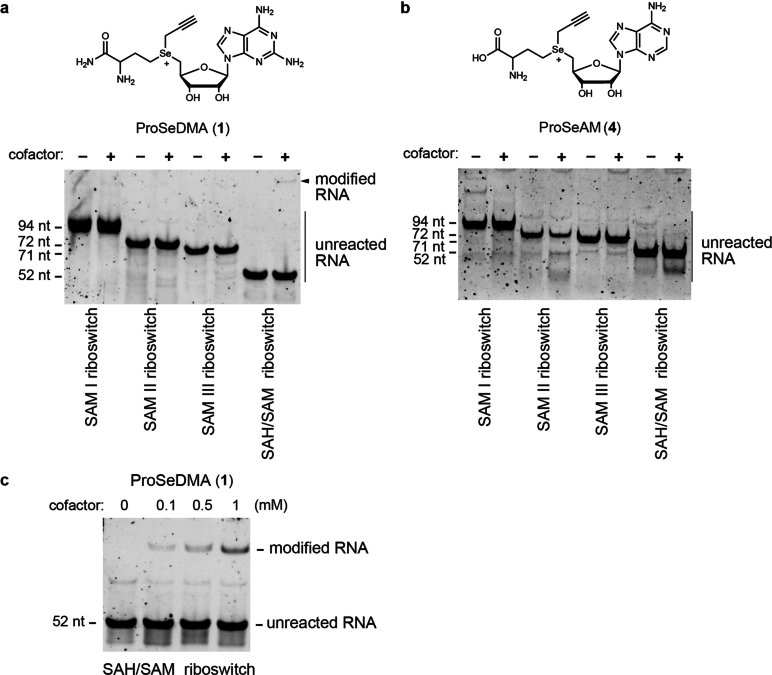
Self-alkylation activity of SAM riboswitches with ProSeDMA and ProSeAM. Streptavidin binding assay of four SAM riboswitches confirmed propargyl modification following copper-catalyzed click reaction with biotin azide. Reaction conditions: For trans- alkylation, 100 pmol riboswitch RNA, 2 nmol ProSeDMA (**a**) or ProSeAM (**b**) were incubated in 20 μL reaction buffer (50 mM HEPES, 120 mM KCl and 5 mM NaCl, 10 mM MgCl_2_) at 37 °C for 15 h. For click reaction, 100 pmol alkylated RNA, 500 μM CuBr, 1 mM TBTA and 1 mM biotin azide were incubated in H_2_O/DMSO/tBuOH = 5/3/1 solution at 37 °C for 1 h. For streptavidin gel shift assay, 5 pmol RNA and 1 μg of streptavidin were incubated in 1× TBS buffer at room temperature for 15 min. **c**. Concentration dependent self- alkylation of the SAM/SAH riboswitch with ProSeDMA. Same conditions were used for streptavidin binding assay. Representative gel images of independent duplicates for all riboswitches. Source data

Four distinct families of natural SAM riboswitches have been identified to date and were shown to regulate key elements of the SAM metabolic pathway through modulation of RNA structure and dynamics upon binding of SAM^47^. Despite comprehensive studies of riboswitch tertiary structures and conformational landscapes, there has been no report of methyl transfer reactions facilitated by either natural or engineered SAM-dependent riboswitches^33,48,49^. A SAM-using synthetic ribozyme was found through in vitro selection to generate m^7^G in its active site^37^. In our previous study, we demonstrated that SAMURI can use SAM as a cofactor to generate m^3^A in the target RNA^43^. To allow for a more thorough comparison between natural SAM riboswitches and synthetic methyltransferase ribozymes, we cocrystallized SAMURI together with SAM and solved the structure of the postcatalytic complex with bound SAH. The overall structure of SAM–SAMURI greatly resembled the ProSeDMA–SAMURI structure (r.m.s.d. = 0.34 Å for 1,564 pairs of atoms) (Fig. 5a). The reacted ligand SAH was bound in a similar manner to SeDHA. Therefore, the structural features of SAMURI discussed above also hold for the SAM–SAMURI complex. To further support the structural comparison of ribozyme and riboswitches, we tested the reactivity of several classes of SAM riboswitches with ProSeDMA or ProSeAM. Of the SAM I, SAM II, SAM III and SAH–SAM riboswitches, only the SAH–SAM riboswitch showed trace amounts of self-propargylation (<10%) when provided with a high concentration of 1 mM ProSeDMA or an extensive incubation time of 15 h (Extended Data Fig. 6). For comparison, SAMURI achieved >90% reaction with 10 µM ProSeDMA in only 1 h (Extended Data Fig. 5).

A notable structural difference between SAMURI and SAM riboswitches was observed in the architecture of their cofactor-binding pockets. In all classes of SAM riboswitches, a characteristic feature is the cavity-like space surrounding the sulfonium atom^50–53^. The generally favorable Coulombic attraction between the positive charge and the RNA is often supported by charge-assisted chalcogen-bonding interactions that result from specific positioning of uracil oxygen atoms in close distance and an almost linear orientation to the σ* of the sulfur carbon bond^54^. This arrangement of the cofactor avoids placing reactive RNA nucleophiles near the electrophilic carbon atom of SAM to prevent self-methylation (Fig. 5e). In contrast, SAMURI exploits a distinct kink in the RNA to expose the *N*^3^ of the target adenosine to the reactive methyl group of SAM (Fig. 5b,c).

**Extended Data Fig. 7:**
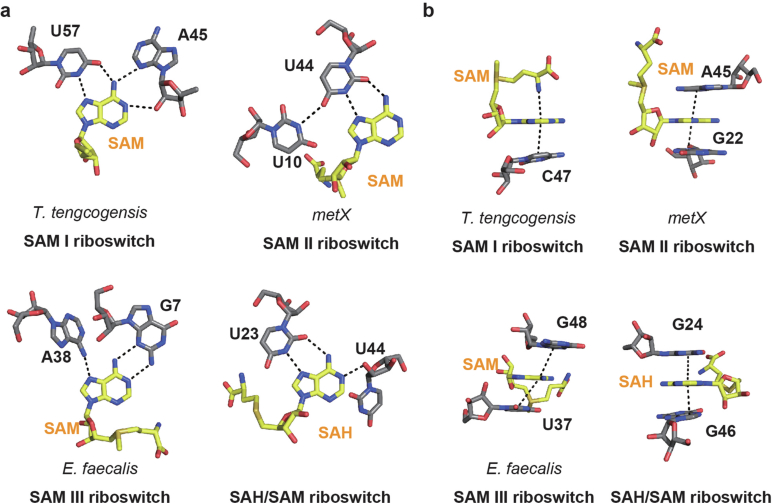
Excerpts of exemplary SAM riboswitch structures. **a, b**. Characteristic interactions involving the adenosine unit in SAM riboswitches displaying H-bond networks (a) and stacking interactions (b). Pymol figures were prepared from the models of *T. tengcogensis* SAM I riboswitch (PDB: 2GIS), metX SAM II riboswitch (PDB: 2QWY), *E. faecalis* SAM III riboswitch (PDB: 3E5C) and the SAM-SAH riboswitch (PDB: 6YL5).

While the adenine nucleobase is rigidified by base stacking and engaged in H-bonding through its Watson–Crick and/or Hoogsteen edges in all SAM riboswitches, the cofactor recognition pattern demonstrates considerable variability for the amino acid unit of SAM (Fig. 5d and Extended Data Fig. 7). Despite the different global orientation and compact versus extended conformations of SAM bound to class I and class II riboswitches, the methionine amino acid terminus engages heavily with the SAM I and SAM II RNAs through electrostatic interactions and H-bonding (Fig. 5d). Consistent with the importance of these noncovalent interactions, SAM derivatives lacking the carboxylic acid or α-amino group in the methionine unit lose the binding affinity to the SAM I (ref. ^55^) and SAM II (ref. ^56^) riboswitches. In contrast, SAM III and SAH–SAM riboswitches and SAMURI exhibit a more relaxed approach to recognition of the amino acid terminus. In particular, SAMURI even performs alkylation in the absence of the methionine unit (Fig. 4), although the suggested interaction between the α-amino group and *O*^2^ of U8 enhances the reactivity. A similar H-bond between the α-amino group and uracil is seen in the structure of the SAM–SAH riboswitch^53^, which was the only one of the tested natural RNA sequences that showed traces of the alkylation product in a concentration-dependent manner (Extended Data Fig. 6). These results suggest that this riboswitch has the potential to be an alkylation ribozyme and encourage the search for other natural RNAs with cofactor-dependent catalytic activities.

## Discussion

The crystal structure of SAMURI reveals a 3HJ of the postcatalytic state, comprising the modified RNA and the reacted cofactor in the active site. Both structures with (1) the propargylated adenosine and SeDHA and (2) the methylated adenosine and SAH display very similar architectures. The catalytic core comprises four parallel layers of nucleobases, each contributing to the stabilization of the structure through *π*–*π* stacking interactions (Fig. 1). In addition, SAMURI uses noncanonical tertiary interaction motifs that have also been found in other functional RNAs. For example, the G9•U37 *trans* base pair features G9 in the *syn* conformation and restricted flexibility of U37 for interaction with the ligand. This attests to the previously reported prevalence of *syn* conformations of purine nucleotides in the active sites of ribozymes and aptamers^57^. The target nucleotide A52 is positioned in the active site by *π*–*π* stacking and is engaged in H-bonding with all nitrogen atoms participating, except *N*^3^, which is the nucleophile that is exposed to the propargyl group at the Se center of the cofactor. This proximity and orientation explain the high specificity of SAMURI for modifying the *N*^3^ of adenosine. The stacking interaction of the propargyl/methyl group with G10 is consistent with the experimentally determined cofactor scope for the size of the transferred alkyl group. Any moieties larger than propargyl would result in a steric clash with the backbone. The negative charge at the kink in the substrate RNA is neutralized by a hydrated magnesium ion, which is found in both molecules of the asymmetric unit in both the ProSeDMA and the SAM structures. Another conserved magnesium ion is located near the Watson–Crick side of the cofactor, which may fine-tune the positioning of the cofactor and reaction layers. The structures indicate that magnesium ions are crucial for proper folding of SAMURI, which is consistent with the observation that SAMURI lost its activity in the absence of magnesium ions^43^ and SAMURI crystals only grew if Mg^2+^ ions were supplemented. Thus, the proposed reaction mechanism follows an S_N_2 pathway on the basis of proximity and in-line positioning but does not involve any obvious metal ion assistance of acid–base catalysis. Similar reaction mechanisms have been proposed for a subset of protein methyltransferases, particularly for class I methyltransferases such as the capping enzyme RNA (*N*^7^-guanine) methyltransferase Ecm1 that installs m^7^G in the mRNA cap^58,59^. However, no methyltransferase protein enzyme has yet been reported to generate m^3^A in RNA nor to remove it through direct or indirect mechanisms. In contrast, for 3-methyladenine in DNA, the specific base excision repair enzyme m^3^A DNA glycosylase has been described^60^.

In both ProSeDMA–SAMURI and SAM–SAMURI structures, the methionine tail of the cofactor points to the open space between the P1 and J2 or J3 elements of the structure. Consequently, the electron density was weakly defined; hence, only a putative interaction between the α-amino group of ProSeDMA or SAM and *O*^2^ of U8 in the RNA could be inferred. This is distinct from class I methyltransferases in which an evolutionarily conserved sequence motif contacts the l-methionine part of SAM^61,62^ and from the Cu^2+^-dependent binding of SAM by the recent in vitro selected ribozyme that generates m^7^G in RNA^37^. However, the combined results from in-line probing, SAMURI mutagenesis experiments and the structure–activity analyses of cofactor variants substantiate the hypothesis that the weak interaction between the α-amino group of ProSeDMA and the pyrimidine oxygen also contributes to enhancing the reaction rate of the SAMURI ribozyme.

While deducing the recognition pattern of ProSeDMA, we refined the cofactor structure and further improved the chemical stability of the propargyl donor. This is a notable finding for further development of SAMURI for cellular applications, in which the generally short half-life of SAM analogs has been recognized as one of the global problems^63^. Surprisingly, we found that complete truncation of the amino acid unit to a methyl group still resulted in detectable propargylation (Fig. 4). This observation raises the exciting possibility that ribozymes may be able to use each of the three substituents at the chalcogen atom for new reactions. Considering that SAM is an extremely versatile cofactor in nature that can donate the methyl, adenosyl or aminoalkyl group in protein-catalyzed biosynthesis reactions, we speculate that analogous reactions may be afforded by RNA catalysis. In this context, it is also interesting to note the recent discovery of a spermidine-responsive riboswitch that has high similarity to class I SAM riboswitches^64^. Furthermore, an unusual methyltransferase enzyme was found in plants (EC 2.1.1.10)^65^ that uses *S*-methylmethionine as the methyl group donor, which is a cofactor that lacks the adenosyl moiety. The existence of such enzyme–cofactor pairs implies the possibility that the propargylated Se-methionine amide (ProSeMA, **13**) could also serve as the alkyl donor. Although **13** was not an active substrate for SAMURI, we expect that a corresponding ribozyme could be found in a new in vitro selection experiment that directly uses propargylselenomethionine or **13** as a cofactor. In short, we predict that a rich diversity of RNA-catalyzed reactions is waiting to be uncovered in the future. The comparison of such new ribozymes with SAMURI, combined with in vitro evolution experiments of partially randomized SAM riboswitches and enrichment of active sequences from genomic or metagenomic RNA libraries may provide further insights into the scope of RNAs catalytic potential and possible ancestors of methyl group donors in a prebiotic world.

## Methods

### Complex formation and crystallization

The 58-nt *cis*-SAMURI RNA (R2; sequence in Supplementary Table 1) was transcribed from a purchased (Microsynth) DNA template (D2; sequence in Supplementary Table 2) with home-made T7 RNA polymerase as described previously^15^. After PAGE purification, the transcript was recovered, diluted to roughly 50 μM in buffer (10 mM HEPES pH 7.5 and 50 mM KCl) and annealed by heating at 95 °C for 5 min and incubation at 22 °C for 10 min. The RNA solution was concentrated by ultrafiltration on a VivaSpin column (Satorius) and washed extensively with water. Afterward, the concentrate was transferred to a fresh tube where construct buffer and cofactor were added and adjusted to a final RNA concentration of 0.4 mM in 10 mM HEPES pH 7.5, 50 mM KCl, 5 mM MgCl_2_ and 0.6 mM ProSeDMA. The construct was incubated at 37 °C for 2 h and stored at −20 °C. The construct with SAM was prepared in the same manner except for changing the cofactor concentration from 0.6 to 3 mM.

Crystals were grown by the hanging drop method by mixing construct solutions and reservoir in a 1:1 ratio with a 1-µl drop volume at 20 °C. Rod-shaped crystals appeared in 2 days and developed to full size within 5 days by equilibrating against a reservoir containing 50 mM MES pH 6.5, 100 mM NaCl, 100 mM LiCl, 15–30 mM MgCl_2_ and 39% MPD. Single crystals were mounted in cryo loops (Hampton Research) and directly flash-frozen without additional cryoprotectants.

### Streptavidin-binding assay of the propargylated RNA derived from crystals

For analysis of the propargylation status of the RNA in the crystal, a few crystals were collected from the drop. After washing with crystal growing buffer, the crystals were dissolved in water and the concentration was measured. Then, 100 pmol of RNA was used for the copper-catalyzed azide–alkyne cycloaddition reaction, where 500 μM CuBr, 1 mM TBTA and 1 mM biotin azide were incubated in a solution of H_2_O, DMSO and *tert*-butyl alcohol (5:3:1, respectively) at 37 °C for 1 h. After ethanol precipitation, labeled RNA was analyzed by a streptavidin gel shift assay by native PAGE (5 pmol RNA and 1 μg of streptavidin in 1× Tris-buffered saline (TBS)).

### Data collection and structure determination

X-ray diffraction experiments were conducted at 100 K and data were collected on Eiger2 X 16M and Eiger X 16M detectors at the P11 or P13 beamlines (German Electron Synchrotron (DESY)), respectively. Reflections were indexed, integrated and scaled with XDS^66^. Scaling was analyzed in AIMLESS and crystal packing was checked with MATTHEWS_COEF from the CCP4 suite (CCP4 7.0 package)^67^. SAD phasing was performed for SAMURI–ProSeDMA crystals using AutoSol in PHENIX (1.20)^68^ with data collected at the Se K-edge at a wavelength of 0.979 Å. The HySS^44^ algorithm found two Se atoms and an initial map was generated by Phaser^45^ (figure of merit (FOM) ≈ 0.3), which was subsequently improved by RESOLVE^46^ resulting in an FOM of 0.72 from which a first model was built. The initial model was incomplete with the P2 and P3 helical regions being placed correctly. The J2/J3 elements could be easily traced and were manually built in Coot (0.9.4)^69^ to generate a partial model for another round of AutoSol, where phases were further improved. The resulting map was superior and additional regions were automatically built. The model was completed and partially corrected by manual building in Coot and refinement with PHENIX^70^ incorporating the experimental phases and turning off the noncrystallographic symmetry averaging in an iterative procedure. The resulting SAMURI–ProSeDMA model was used as search model for molecular replacement in PHENIX Phaser to solve the SAMURI–SAM crystal structure. Data collection and refinement statistics of both structures are summarized in Supplementary Table 4. Restraints of ligands and special nucleotides (5′ terminal guanine diphosphate, propargylated adenosine, m^3^A and reacted cofactor SeDHA) were generated in eLBOW^71^ of PHENIX. Figures presented in this article were prepared using PyMol (Schrödinger, version 4.6.0).

### In-line probing

Probing experiments were performed in a bimolecular setup where an excess of substrate R3 (25 pmol) was mixed with 5′-^32^P -labeled ribozyme R4. The mixture was denatured at 95 °C for 4 min and annealed by cooling gradually to 22 °C over 10 min. In-line buffer was added to a final concentration of 20 mM Tris-HCl pH 8.0, 20 mM KCl and 20 mM MgCl_2_ along with cofactors at various concentrations in a final reaction volume of 5 μl. Reactions progressed at 20 °C for 16–18 h and were quenched by addition of 5 μl of loading dye (80% formamide, 89 mM Tris-HCl, 89 mM boric acid, 52 mM EDTA, 0.025% (w/v) bromophenol blue and 0.025% xylene cyanol). The samples were applied to a 20% denaturing PAGE with reference R4 treated by RNase T1 digestion and alkaline hydrolysis and run for 160 min at 45 W. RNase T1 digestion was performed by incubating ^32^P -labeled ribozyme R4 with RNase T1 enzyme (final concentration: 0.5 U per μl; Thermo Scientific) in 50 mM Tris-HCl pH 7.5 at 37 °C for 20 s and immediately quenched by adding an equal volume of loading dye. For alkaline hydrolysis, labeled ribozyme mixed in 20 mM NaOH was incubated at 95 °C for 4 min and subsequently quenched by the addition of loading dye. The sequencing gel was dried at 80 °C under vacuum and visualized by autoradiography (Amersham, Typhoon). All in-line probing experiments were carried out as three independent experiments.

### Kinetic assays of SAMURI-catalyzed transpropargylation reactions with ProSeDMA derivatives

First, 10 pmol of Cy5-labeled substrate R1 (Supplementary Table 3) and 100 pmol of ribozyme R4 were annealed in reaction buffer (50 mM HEPES, 120 mM KCl and 5 mM NaCl). Then, 10 mM MgCl_2_ and 100 pmol of the respective cofactor (synthesis described in Supplementary Note) were added and the reaction mixture was adjusted with water to a final volume of 10 µl. The mixture was incubated at 37 °C and 1-µl aliquots were taken into 4 µl of stop solution (80% formamide, 89 mM Tris-HCl, 89 mM boric acid and 50 mM EDTA) at the desired time points. Then, 2 µl of sample at each time point was applied on the 20% denaturing PAGE. The modified RNA showed a mobility shift (slower migration than the starting material) and the band intensity was quantified by fluorescence image using a 695/55-nm emission filter. The yield versus time data were fitted to *Y* = *Y*_max_(1 − exp(−*k*_obs_*t*)) using Origin (2019). All kinetic assays were carried out as three independent experiments.

### Stability assay of ProSeDMA analogs by HPLC analysis

First, 1 mM ProSeDMA or ProSeDAB was mixed in a total volume of 100 μl of reaction buffer (50 mM HEPES, 120 mM KCl, 5 mM NaCl and 10 mM MgCl_2_, pH 7.0). The reaction mixtures were incubated at 37 °C and 20-μl aliquots were taken from the samples and added to 40 μl of a solution of H_2_O and 0.1% trifluoroacetic acid (TFA) after 15 h. The collected samples were analyzed by RP-HPLC (NUCLEOSIL 100-5 C18 column; 5 μm, 125 × 4 mm). The analysis was run with a linear gradient of 5–7% B (0–15 min) and 7–70% B (15–30 min). Solvent A was H_2_O and 0.1% TFA; solvent B was acetonitrile and 0.1% TFA. The flow rate was 0.7 ml min^−1^ at 30 °C with ultraviolet detection at 260 nm.

### Reporting summary

Further information on research design is available in the Nature Portfolio Reporting Summary linked to this article.

## Online content

Any methods, additional references, Nature Portfolio reporting summaries, source data, extended data, supplementary information, acknowledgements, peer review information; details of author contributions and competing interests; and statements of data and code availability are available at 10.1038/s41589-024-01808-w.

## Supplementary information

Supplementary Information Supplementary Tables 1–4 and Note (chemical synthesis).

Reporting Summary

## Source data

Source Data Fig. 1 Unprocessed gel.

Source Data Fig. 2 Unprocessed gels.

Source Data Fig. 4e Unprocessed gel.

Source Data Fig. 4d Statistical source data.

Source Data Extended Data Fig. 3 Unprocessed gels.

Source Data Extended Data Fig. 4 Unprocessed gels.

Source Data Extended Data Fig. 4b Statistical source data.

Source Data Extended Data Fig. 5 Unprocessed gels.

Source Data Extended Data Fig. 6 Unprocessed gels.

## Data Availability

The atomic coordinates and structure factors were deposited to the PDB (www.rcsb.org) under accession codes 9FN3 for ProSeDMA–SAMURI and 9FN2 for SAM–SAMURI. Existing structures used throughout the study were also obtained from the PDB under accession codes 2GIS, 2QWY, 3EC5 and 6YL5. DNA and RNA sequences are provided in Supplementary Tables 1–3 and data collection and refinement statistics are provided in Supplementary Table 4. The Supplementary Note contains synthetic procedures and nuclear magnetic resonance spectra of synthetic intermediates and final cofactors. Source data are provided with this paper.
