# Supplementary material for: Structure and catalytic activity of the SAM-utilizing ribozyme SAMURI
Source: Nat Chem Biol. 2025 Jan 8;22(6):938–47. doi: 10.1038/s41589-024-01808-w (PMC13226088; doi:10.1038/s41589-024-01808-w)
Supplement: Supplementary file 1 — Supplementary Tables 1–4 and Note (chemical synthesis). [file 41589_2024_1808_MOESM1_ESM.pdf]

nature chemical biology

Supplementary information

https://doi.org/10.1038/s41589-024-01808-w

Structure and catalytic activity of the SAM-utilizing ribozyme SAMURI

In the format provided by the authors and unedited

Table of contents page

Supplementary Tables

Supplementary Table 1: RNA oligonucleotides prepared by solid-phase synthesis 1

Supplementary Table 2: DNA oligonucleotides used as primers and templates 1

Supplementary Table 3: ESI-MS data for RNA substrates 1

Supplementary Table 4: Data collection and refinement statistics 2

Supplementary Note 3

Synthetic procedures for ProSeDMA analogues 4

NMR spectra 24

Supplementary References 48

Supplementary Tables

Supplementary Table 1. RNA oligonucleotides: substrate RNA and important transcript sequences

No.	Description	5′-sequence-3′
R1	Substrate RNA for *trans*_SAMURI	ACAUACUGAGCCUUCAA-NH2
R2	*cis*-SAMURI for xtal (transcript from D2)	GGAGUCAUGGCUCAGGGCUGUUCGCAGCCGCUGCAGUCAGUCGAAAGACUGAG ACUCC
R3	Substrate RNA for in-line probing	ACAUACUGAGCCUUCAA
R4	*trans*-SAMURI (transcript from D3, WT)	GGUUGAAGGCAUGGCUCAGGGACUUCGGUCCGCUGCAGUCAGUAUGUCC
R5	Substrate RNA for *trans*_SAMURI (A9G)	AlK-GACAUACUGGGCCUUCAAAUA
R6	Substrate RNA for *trans*_SAMURI (A9I)	ACAUACUGIGCCUUCAA-NH2
R7	*trans*-SAMURI (C11U, G36A)	GGUUGAAGGCAUGGUUCAGGGACUUCGGUCCGCUGCAAUCAGUAUGU
R8	*trans*-SAMURI (ΔA7)	GGUUGAAGGC UGGCUCAGGGACUUCGGUCCGCUGCAGUCAGUAUGU
R9	*trans*-SAMURI (ΔA7, U8C)	GGUUGAAGGC CGGCUCAGGGACUUCGGUCCGCUGCAGUCAGUAUGU
R10	*trans*-SAMURI (ΔA7, U8G)	GGUUGAAGGC GGGCUCAGGGACUUCGGUCCGCUGCAGUCAGUAUGU
R11	*trans*-SAMURI (ΔA7, U8A)	GGUUGAAGGC AGGCUCAGGGACUUCGGUCCGCUGCAGUCAGUAUGU
R12	*trans*-SAMURI (ΔA7, ΔU8)	GGUUGAAGGC GGCUCAGGGACUUCGGUCCGCUGCAGUCAGUAUGU
R13	*trans*-SAMURI (U12G, A35C)	GGUUGAAGGCAUGGCGCAGGGACUUCGGUCCGCUGCCGUCAGUAUGU
R14	*trans*-SAMURI (A14U, U32A)	GGUUGAAGGCAUGGCUCUGGGACUUCGGUCCGCAGCAGUCAGUAUGU
R15	*trans*-SAMURI (ΔA14, ΔU32)	GGUUGAAGGCAUGGCUC GGGACUUCGGUCCGC GCAGUCAGUAUGU

Supplementary Table 2. DNA oligonucleotides: Primers and transcription templates

No	Description	5′-sequence-3′
D1	T7 promotor	CTGTAATACGACTCACTATA
D2	Template (*cis*-active SAMURI for Xtal)	GGAGTCTCAGTCTTTCGACTGACTGCAGCGGCTGCGAACAGCCCTGAGCCATG ACTCCTATAGTGAGTCGTATTACAG
D3	Template (*trans*-active SAMURI, WT)	GGACATACTGACTGCAGCGGACCGAAGTCCCTGAGCCATGCCTTCAACCTATA GTGAGTCGTATTACAG
D4	Template (*trans*-active mut.SAMURI ΔA7)	ACATACTGACTGCAGCGGACCGAAGTCCCTGAGCCAGCCTTCAACCTATAGTG AGTCGTATTACAG
D5	Template (*trans*-active mut.SAMURI ΔA7, U8C)	ACATACTGACTGCAGCGGACCGAAGTCCCTGAGCCGGCCTTCAACCTATAGTG AGTCGTATTACAG
D6	Template (*trans*-active mut.SAMURI ΔA7, U8G)	ACATACTGACTGCAGCGGACCGAAGTCCCTGAGCCCGCCTTCAACCTATAGTG AGTCGTATTACAG
D7	Template (*trans*-active mut.SAMURI ΔA7, U8A)	ACATACTGACTGCAGCGGACCGAAGTCCCTGAGCCTGCCTTCAACCTATAGTG AGTCGTATTACAG
D8	Template (*trans*-active mut.SAMURI ΔA7, ΔU8)	ACATACTGACTGCAGCGGACCGAAGTCCCTGAGCCGCCTTCAACCTATAGTGA GTCGTATTACAG
D9	Template (*trans*-active mut.SAMURI U12G, A35C)	ACATACTGACGGCAGCGGACCGAAGTCCCTGCGCCATGCCTTCAACCTATAGT GAGTCGTATTACAG
D10	Template (*trans*-active mut.SAMURI A14U, U32A)	ACATACTGACTGCTGCGGACCGAAGTCCCAGAGCCATGCCTTCAACCTATAGT GAGTCGTATTACAG
D11	Template (*trans*-active mut.SAMURI ΔA14, ΔU32)	ACATACTGACTGCGCGGACCGAAGTCCCGAGCCATGCCTTCAACCTATAGTGA GTCGTATTACAG
D12	Template (SAM I riboswitch)	TGGCTCATCTTTCAACGTTTCCGCTGCAGGAATTGGCACCATTTCTGGTTGCC GGGTTTCATCGGGCCAGTCCCTCCACCTCTCTTGATAAGCCTATAGTGAGTCG TATTACAG
D13	Template (SAM II riboswitch)	CCCTTTTTAGCTACATTTATCACGCGCTTGCAATACGGTTAAATCAGCGCGAC CTATAGTGAGTCGTATTACAG
D14	Reverse primer (SAM II riboswitch)	GGTAAGGTGGACATACTGCCCTTTTTAGCTACATTTAT
D15	Template (SAM III riboswitch)	ATTCCCCCTTTCGGTTACAAGGCATCTGGCGTTTCCGCCATCCTTTCGGGAAC TATAGTGAGTCGTATTACAG
D16	Reverse primer (SAM III riboswitch)	GGTAAGGTGGACATACTGATTCCCCCTTTCGGTTACAA
D17	Template (SAM/SAH riboswitch)	TTGCTCCACTGAGGTCACCTCGTCACGCCAGGAAGCCGTTGTGACAGGTACC TATAGTGAGTCGTATTACAG

Supplementary Table 3. ESI-MS analysis of synthetic RNAs

No.	Description	Chemical formula	Mass calculated	Mass found
R1	Substrate RNA for SAMURI	$C_{167}H_{215}N_{64}O_{118}P_{17}$	5530.83 Da	5530.86 Da
R1Cy5	Substrate RNA for SAMURI with Cy5	$C_{199}H_{251}N_{66}O_{125}P_{17}S_2$	6155.03 Da	6155.04 Da

Supplementary Table 4. Data collection and refinement statistics

	SAMURI-ProSeDMA	SAMURI-SAM
PDB code	9FN3	9FN2
**Data collection**		
Wavelength	0.9790	0.9763
Resolution (Å)	42.92 - 2.90 (3.08 - 2.90)*	43.10 - 2.90 (3.08 - 2.90)
Space group	P 4_2	P 4_2
Unit cell		
a, b, c (Å)	75.44 75.44 72.25	75.44 75.44 73.15
α, β, γ (°)	90 90 90	90 90 90
Unique reflections	18088 (2901)	9208 (1471)
Multiplicity	14.0 (14.6)	13.9 (14.0)
Completeness (%)	99.9 (100)	99.9 (100)
R_{merge}	0.060 (2.112)	0.051 (1.180)
R_{pim}	0.020 (0.593)	0.015 (0.326)
Mean I/sigma(I)	22.1 (1.2)	24.9 (2.4)
$CC_{1/2}$	99.9 (59.1)	100 (83.3)
Wilson B-factor (Å^2)	126.4	113.0
No. mol / ASU	2	2
**Refinement**		
Resolution (Å)	42.92 - 2.90 (3.12 - 2.90)	33.74-2.90 (3.32-2.90)
No. reflections for Rwork	15568 (2522)	8699 (2895)
No. reflections for R-free	743 (126)	489 (139)
Rwork / R-free	19.91 / 22.96	20.42 / 23.03
Coordinate error	0.30	0.26
No. non-hydrogen atoms	2590	2593
RNA	2498	2494
Ligands / ions / water	92	99
Average B-factor (Å^2)	51.57	52.09
Chain A (Å^2)	51.12	56.22
Chain B (Å^2)	52.60	48.67
Water (Å^2)	29.70	30.78
r.m.s. deviations		
Bond length (Å)	0.002	0.002
Bond angles (Å)	0.565	0.556
Clashscore	1.58	1.05
Number of TLS groups	2	2

*Statistics for the highest-resolution shell are shown in parentheses.

Supplementary Note

General materials and methods

All standard chemicals and solvents were purchased from commercial suppliers. Dry solvents (dichloromethane, THF, DMF, acetonitrile) were obtained from a solvent purification system (SPS). Water for in vitro experiment was obtained from a Sartorius Arium® pro ultrapure water system. Silica gel plates coated with fluorescent indicator were used for thin layer chromatography (TLC) and the plates were visualized with UV light. Silica gel (Kieselgel 60, Merck, 0.063 – 0.200 mm) was used for column chromatography. NMR spectra were measured on a Bruker Avance III HD 400 spectrometer at 400 MHz. Spectral assignments were verified by 2D NMR experiments. High resolution ESI mass spectra were measured on a Bruker micrOTOF-Q III spectrometer. Monoisotopic masses for oligonucleotides were obtained by charge deconvolution of the raw spectra. Unmodified DNA oligonucleotides were purchased from Microsynth and purified by PAGE. Fluorophores were purchased from lumiprobe. All stock solutions for crystallization were filtered through 0.22 mM syringe filters (Avantor). Initial screening was performed with Helix at 20°C (Molecular Dimensions). Hanging-drop fine screens were done in 24-well VDX plates (Hampton research). All other chemicals were purchased from Sigma-Aldrich or ABCR and used without further purification. HPLC grade solvents were purchased from VWR. Fluorescent imaging of the kinetic and activity assay gels was taken using a BioRad Chemidoc gel-documentation device.

Synthesis of ProSeDMA analogues

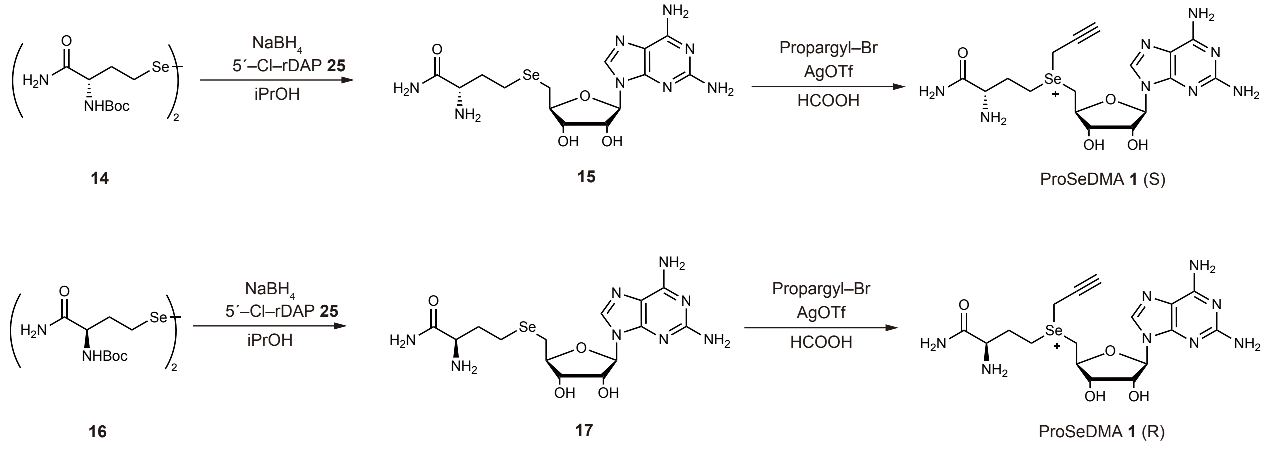

Supplementary Scheme 1. Synthesis of ProSeDMA (R) and (S)

Se-2,6-Diaminopurineribosyl-L-selenohomocysteineamide (15)
Se-2,6-Diaminopurineribosyl-D-selenohomocysteineamide (17)

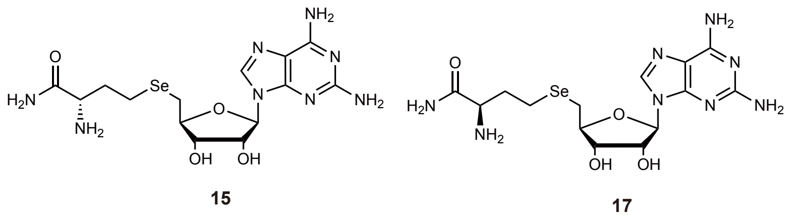

Compounds **14** or **16** were synthesized from stereo pure amino-γ-butytolactone followed by previous report[1]. Starting material **14** or **16** **(**10 mg, 17 µmol**)** was activated by sodium borohydride (7.5 mg, 198 µmol) in *i*PrOH (1 mL). After 15 min, 9-(5′-chloro-5′-deoxy-β-D-ribofuranosyl)-2,6-diamiopurine (= 5′-chloro-2-aminoadenosine) (9 mg, 30 µmol) was added and the reaction was proceeded for 2 h with heating under reflux. The reaction mixture was diluted by DCM and extracted with water. The aqueous phase was dried by evaporation and the residue was dissolved in formic acid (1 mL). After overnight Boc-deprotection, the solvent was removed and the crude mixture was purified by C18 reverse phase HPLC (2 mL/min, NUCLEODUR® C18 Pyramid VP 250/21 column, linear gradient B conc. 5% - 7% (0 min to 15 min), 7% - 70% (15min to 30 min); solvent A was H_2O + 0.1% TFA; solvent B was MeCN + 0.1% TFA; flow rate was 2 ml/min at 30 °C with UV detection at 260 nm.) Fractions containing the desired material were combined and the solvent was removed by lyophilization. Yield: 12.6 µmol (determined by UV absorbance at 279 nm, $\varepsilon_{279\ nm}$ = 9,894 $Lmol^{-1}cm^{-1}$) of **15** as white foam (42 %).

^{1}H NMR (400 MHz, D_2O) δ 8.03 (s, 1H), 5.80 (d, *J* = 4.9 Hz, 1H), 4.74 – 4.72 (m, 1H), 4.29 (t, *J* = 4.9 Hz, 1H), 4.22 – 4.18 (m, 1H), 3.97 – 3.94 (m, 1H), 2.95 – 2.91 (m, 2H), 2.58 – 2.52 (m, 2H), 2.14 – 2.04 (m, 2H). **^{13}C NMR** (100 MHz, D_2O) δ 171.31, 152.28, 151.60, 149.81, 140.36, 111.56, 87.58, 83.74, 73.05, 72.87, 52.81, 31.67, 25.58, 17.98.
ESI-MS (*m/z*): $[M+H]^+$ calcd for $C_{14}H_{23}N_8O_4Se$, 447.1002; found 447.1021.

10.8 µmol (determined by UV absorbance at 279 nm, $\varepsilon_{279\ nm}$ = 9,894 $Lmol^{-1}cm^{-1}$) of **17** as white foam (36 %).
^{1}H NMR (400 MHz, D_2O) δ 8.03 (s, 1H), 5.80 (d, *J* = 4.9 Hz, 1H), 4.74 – 4.71 (m, 1H), 4.29 (t, *J* = 4.9 Hz, 1H), 4.23 – 4.18 (m, 1H), 3.97 – 3.94 (m, 1H), 2.99 – 2.89 (m, 2H), 2.60 – 2.49 (m, 2H), 2.14 – 2.04 (m, 2H). **^{13}C NMR** (100 MHz, D_2O) δ 171.30, 152.26, 151.60, 149.81, 140.32, 111.55, 87.57, 83.71, 73.03, 72.86, 52.78, 31.67, 25.54, 17.96.
ESI-MS (*m/z*): $[M+H]^+$ calcd for $C_{14}H_{23}N_8O_4Se$, 447.1002; found 447.1024.

Propargylic *Se*-2,6-diaminopurineribosyl-L-selenomethionineamide (S)
Propargylic *Se*-2,6-diaminopurineribosyl-D-selenomethionineamide (R)

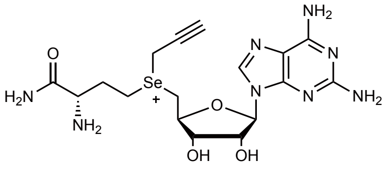

ProSeDMA **1** (S)

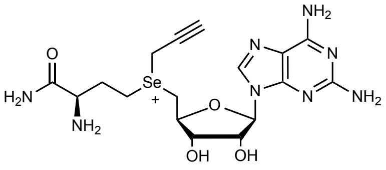

ProSeDMA **1** (R)

Compound **15** or **17** (4.5 mg, 10 µmol) was dissolved in formic acid (1 mL) and the solution was cooled in an ice bath. Propargyl bromide (80% in toluene, ca. 9.2 mol/L, 109 µL) and silver triflate (13 mg, 50 µmol) were added and the reaction mixture was stirred for 48 h. The reaction was quenched by MeOH (500 µL) and the solvent was removed by evaporation. The residue was suspended in H_2O and insoluble Ag salt was filtered off. The crude mixture was purified by C18 reverse phase HPLC (2 mL/min, NUCLEODUR® C18 Pyramid VP 250/21 column, linear gradient B conc. 5% - 7% (0 min to 15 min), 7% - 70% (15min to 30 min); solvent A was H_2O + 0.1% TFA; solvent B was MeCN + 0.1% TFA; flow rate was 2 ml/min at 30 °C with UV detection at 260 nm. Fractions containing the desired material were combined and the solvent was removed by lyophilization.
Yield: 1.6 µmol (determined by UV absorbance at 279 nm, $\varepsilon_{279\ nm}$ = 9,894 $Lmol^{-1}cm^{-1}$) of **1 (S)** as white foam (16 %).

^{1}**H NMR** (400 MHz, D_2O) δ 7.98 – 7.97 (m, 1H), 5.87 – 5.84 (m, 1H), 4.74 – 4.71 (m, 1H), 4.60 – 4.52 (m, 1H), 4.44 – 4.39 (m, 1H), 4.14 – 3.75 (m, 2H), 3.39 – 3.33 (m, 2H), 3.12 – 3.05 (m, 1H), 2.34 – 2.24 (m, 2H). ^{13}**C NMR** (100 MHz, D_2O) δ 170.04, 154.91, 154.12, 152.81, 140.94, 111.93, 89.52, 79.13, 78.54, 73.01, 72.99, 70.57, 51.73, 41.19, 36.32, 33.10, 25.90.
ESI-MS (*m/z*): $[M]^+$ calcd for $C_{17}H_{25}N_8O_4Se$, 485.1158; found 485.1155.

1.2 μmol (determined by UV absorbance at 279 nm, $\varepsilon_{279\ nm}$ = 9,894 $Lmol^{-1}cm^{-1}$) of **1 (R)** as white foam (12 %).
^{1}**H NMR** (400 MHz, D_2O) δ 7.97 – 7.96 (m, 1H), 5.87 – 5.85 (m, 1H), 4.76 – 4.74 (m, 1H), 4.61 – 4.52 (m, 1H), 4.44 – 4.40 (m, 1H), 4.14 – 3.78 (m, 5H), 3.12 – 3.05 (m, 1H), 2.90 – 2.78 (m, 2H), 2.30 – 2.23 (m, 2H). Due to low sample amount, signal was not observed in ^{13}**C NMR.**
ESI-MS (*m/z*): $[M]^+$ calcd for $C_{15}H_{25}N_8O_4Se$, 461.1158; found 461.1151.

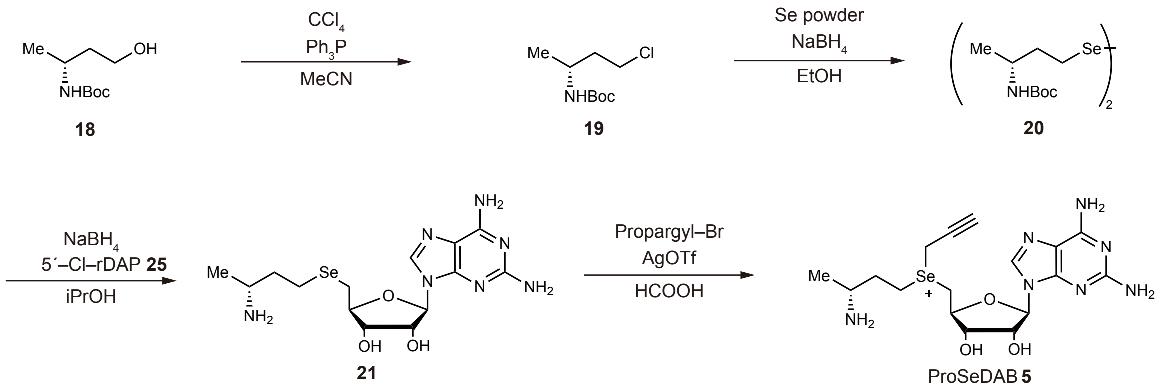

Supplementary Scheme 2. Synthesis of ProSeDAB

N$^{\alpha}$-Boc-(R)-2-Amino-4-chlorobutan (19)

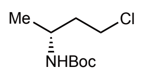

19

To a solution of compound **18**[2] (1 g, 5.3 mmol) in dry acetonitrile (50 mL), CCl_4 (612 µL, 6.3 mmol) and triphenylphosphine (2 g, 7.6 mmol) were added under stirring at room temperature. The reaction was continued for 1 h with heating under reflux. After quenching with MeOH, solvent was removed by evaporation and the residue was extracted with EtOAc / saturated $NaHCO_3$ aq., water, and brine. The organic layer was dried over Na_2SO_4 and evaporated. The crude product was purified by column chromatography on SiO_2 with Hexane / EtOAc = 8 / 2. Yield: 502 mg of **19** as white solid (46%).
^{1}H NMR (400 MHz, DMSO-d6) δ 6.77 (d, J = 8.5 Hz, 1H), 3.65–3.57 (m, 3H), 1.78 (m, 2H), 1.38 (s, 9H), 1.03 (d, J = 6.6 Hz, 3H). **^{13}C NMR** (100 MHz, DMSO) δ 155.51, 77.96, 44.06, 42.86, 39.39, 28.72, 21.06.
ESI-MS (*m/z*): $[M+Na]^+$ calcd for $C_9H_{18}ClNNaO_2$, 230.0918; found 230.0906.

Bis(N$^{\alpha}$-boc-(R)-2-amino-butyl)-4, 4′-diselenido (20)

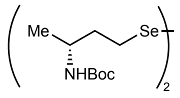

20

To a suspension of selenium powder (450 mg, 5.7 mmol) in ethanol (20 mL), sodium borohydride (143 mg, 3.8 mmol) was added. After stirring for 30 min at room temperature, compound **19** (400 mg, 1.9 mmol) was added in the solution and the reaction was allowed to proceed next 15 h. The solvent

was switch to EtOAc and extracted by saturated $NaHCO_3$ aq., water, brine. The organic layer was filtered by cerite pad and dried by Na_2SO_4 and evaporated. The crude product was purified by column chromatography on SiO_2 with Hexane / EtOAc = 8 / 2. Yield: 303 mg of **20** as slightly yellow foam (61 %).

^{1}H NMR (400 MHz, DMSO-d6) δ 6.74 (d, J = 8.5 Hz, 1H), 3.57–3.48 (m, 1H), 2.87 (t, J = 7.6 Hz, 2H), 1.82–1.69 (m, 2H), 1.38 (s, 9H), 1.03 (d, J = 6.6 Hz, 3H). **^{13}C NMR** (100 MHz, DMSO) δ 155.52, 77.85, 46.14, 38.26, 28.74, 26.64, 21.07.

ESI-MS (*m/z*): $[M+Na]^+$ calcd for $C_{18}H_{36}N_2NaO_4Se_2$ 527.0897; found 527.0922.

Se-2,6-Diaminopurineribosylseleno-2-(R)-amino-butane (21)

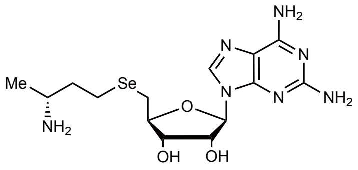

21

Compound **20 (**100 mg, 190 µmol**)** was activated by sodium borohydride (15 mg, 397 µmol) in *i*PrOH (2 mL). After 30 min, 5′-chloro-2-aminoadenosine (120 mg, 400 µmol) was added and the reaction was proceeded for 2 h with heating under reflux. The reaction mixture was diluted by DCM and extracted with water. The aqueous phase was dried by evaporation and the residue was dissolved in formic acid (1 mL). After overnight Boc-deprotection, the solvent was removed and the crude mixture was purified by C18 reverse phase HPLC (2 mL/min, NUCLEODUR® C18 Pyramid VP 250/21 column, linear gradient B conc. 5% - 7% (0 min to 15 min), 7% - 70% (15min to 30 min); solvent A was H_2O + 0.1% TFA; solvent B was MeCN + 0.1% TFA; flow rate was 2 ml/min at 30 °C with UV detection at 260 nm.) Fractions containing the desired material were combined and removal of solvent by lyophilization. Yield: 42 µmol (determined by UV absorbance at 279 nm, $\varepsilon_{279\ nm}$ = 9,894 $Lmol^{-1}cm^{-1}$) of **21** as white foam (11 %).

^{1}H NMR (400 MHz, D_2O) δ 8.01 (s, 1H), 5.72 (d, J = 4.8 Hz, 1H), 4.65 (dd, J = 5.0, 4.8 Hz, 1H), 4.21 (dd, J = 5.0, 4.8 Hz, 1H), 4.13 (dt, J = 6.9, 5.0 Hz, 1H), 3.27–3.19 (m, 1H) , 2.89–2.78 (m, 2H), 2.54 – 2.40 (m, 2H), 1.85 – 1.67 (m, 2H), 1.06 (d, J = 6.6 Hz, 3H). **^{13}C NMR** (100 MHz, D_2O) δ 152.12, 151.40, 149.46, 140.24, 110.86, 87.75, 83.82, 73.11, 72.82, 47.46, 34.50, 25.34, 19.10, 16,96.

ESI-MS (*m/z*): $[M+H]^+$ calcd for $C_{14}H_{24}N_7O_3Se$, 418.1101; found 418.1125.

Propargylic *Se*-2,6-Diaminopurineribosylseleno-2-(R)-amino-butane (5)

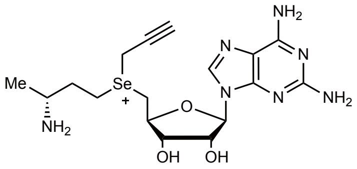

ProSeDAB **5**

Compound **21** (17 mg, 41 μmol) was dissolved in formic acid (5 mL) and the solution was cooled on ice bath. Propargyl bromide (80% in toluene, ca. 9.2 mol/L, 400 μL) and silver triflate (48 mg, 187 μmol) were added and the reaction mixture was stirred next 24 h. The reaction was quench by MeOH (500 μL) and extracted with hexane. The solvent was removed by evaporation. The residue was suspended in H_2O and insoluble Ag salt was filtered off. The crude mixture was purified C18 reverse phase HPLC (2 mL/min, NUCLEODUR® C18 Pyramid VP 250/21 column, linear gradient B conc. 5% - 7% (0 min to 15 min), 7% - 70% (15min to 30 min); solvent A was H_2O + 0.1% TFA; solvent B was MeCN + 0.1% TFA; flow rate was 2 ml/min at 30 °C with UV detection at 260 nm.) Fractions containing the desired material were combined and removal of solvent by lyophilization. Yield: 28 μmol (determined by UV absorbance at 279 nm, $\varepsilon_{279\,nm}$ = 9,894 $Lmol^{-1}cm^{-1}$) of **5** as white foam (68 %).

^{1}H NMR (400 MHz, D_2O) δ 7.98 (s, 1H), 5.83 – 5.81 (m, 1H), 4.74 – 4.71 (m, 1H), 4.57 – 4.50 (m, 1H), 4.41 – 4.36 (m, 1H), 4.09 – 3.73 (m, 4H), 3.37 – 3.22 (m, 3H), 3.07 – 3.00 (m, 1H), 2.12 – 1.85 (m, 2H), 1.18 – 1.06 (m, 3H).

^{13}C NMR (100 MHz, D_2O) δ 152.09, 151.39, 149.63, 140.96, 111.74, 89.46, 80.45, 78.95, 73.19, 72.77, 70.66, 46.80, 41.05, 34.32, 28.97, 25.59, 16.70.

ESI-MS (*m/z*): $[M]^+$ calcd for $C_{17}H_{26}N_7O_3Se^+$, 456.1257; found 456.1260.

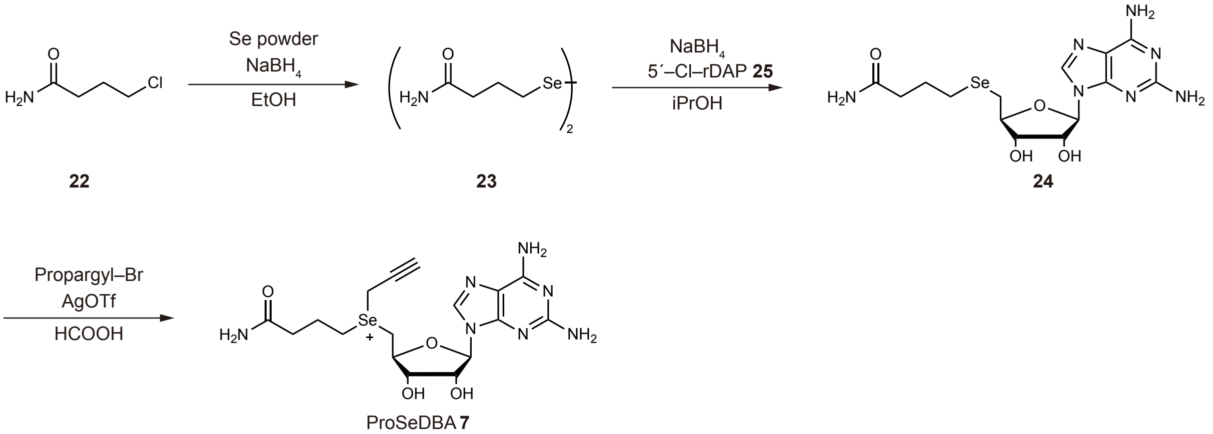

Supplementary Scheme 3. Synthesis of ProSeDBA

4,4′-diselanediylbutanamide (23)

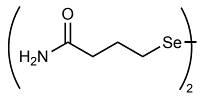

23

To a suspension of selenium powder (970 mg, 12.3 mmol) in ethanol (20 mL), sodium borohydride (310 mg, 8.2 mmol) was added. After stirring for 30 min at room temperature, 4-chlorobutanamide **22** (500 mg, 4.1mmol, BLD pharm) was added in the solution and the reaction was allowed to proceed next 15 h. The solvent was switch to EtOAc and extracted by saturated $NaHCO_3$ aq., water, brine. The organic layer was dried by Na_2SO_4 and evaporated. The crude product was purified by column chromatography on SiO_2 with DCM / EtOAc = 2 / 8. Yield: 256 mg of **23** as slightly yellow foam (36 %).

^{1}H NMR (400 MHz, DMSO-d6) δ 7.46 (d, J = 50.8 Hz, 1H), 7.33 (d, J = 50.8 Hz, 1H), 3.63 (t, J = 13.2 Hz, 2H), 2.20 (t, J = 7.3 Hz, 2H), 1.91 – 1.89 (m, 2H). **^{13}C NMR** (100 MHz, DMSO) δ 173.73, 45.54, 32.45, 28.51.

ESI-MS (*m/z*): $[M+Na]^+$ calcd for $C_8H_{16}N_2NaO_2Se_2$ 354.9434; found 354.9430.

_Se_-2,6-Diaminopurineribosyl-selenobutanamide (24)

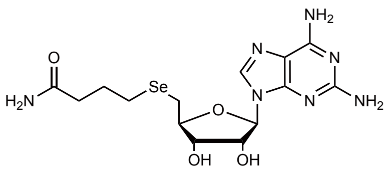

24

Compound **23** (100 mg, 303 µmol) was activated by sodium borohydride (22 mg, 582 µmol) in *i*PrOH (3 mL). After 30 min, 5′-chloro-2-aminoadenosine (174 mg, 580 µmol) was added and the reaction was proceeded for 2 h with heating under reflux. The reaction mixture was diluted by DCM and extracted water. The aqueous phase was dried by evaporation and the crude mixture was purified C18 reverse phase HPLC (2 mL/min, NUCLEODUR® C18 Pyramid VP 250/21 column, linear gradient B conc. 5% - 7% (0 min to 15 min), 7% - 70% (15min to 30 min); solvent A was H_2O + 0.1% TFA; solvent B was MeCN + 0.1% TFA; flow rate was 2 ml/min at 30 °C with UV detection at 260 nm.) Fractions containing the desired material were combined and removal of solvent by lyophilization. The property of **24** was similar as the 5′-chloro-2,6-diaminopurineribose and they were eluted at the same retention time on the reverse phase HPLC. Yield: 12 µmol (determined by UV absorbance at 279 nm, $\varepsilon_{279\ nm}$ = 9,894 $Lmol^{-1}cm^{-1}$.) of **24** as white foam (2 %) with 3 µmol 5′-chloro-2-aminoadenosine (analyzed by signal ration of H8 on diaminopurine).

^{1}H NMR (400 MHz, D_2O) δ 8.01 (s, 1H), 5.77 (d, *J* = 5.0 Hz, 1H), 4.69 – 4.78 (m, 1H), 4.61 (t, *J* = 5.0 Hz, 1H), 4.32 – 4.21 (m, 1H), 3.81 – 3.72 (m, 2H), 2.43 (t, *J* = 7.4 Hz, 2H), 2.22 – 2.11 (m, 2H), 1.79 – 1.66 (m, 2H). **^{13}C NMR** (100 MHz, D_2O) δ 179.00, 152.16, 151.40, 149.49, 139.98, 110.93, 87.63, 83.43, 73.30, 70.66, 43.99, 34.87, 25.89, 22.25.

ESI-MS (*m/z*): $[M+H]^+$ calcd for $C_{14}H_{22}N_7O_4Se$, 432.0893; found 432.0896.

_Se_-Propargyl- _Se_-2,6-Diaminopurineribosyl-selenobutanamide (7)

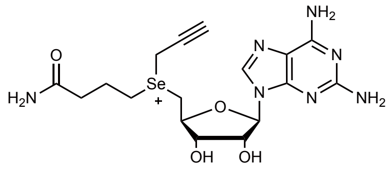

ProSeDBA **7**

Compound **24** (5 mg, 12 µmol) was dissolved in formic acid (1 mL) and the solution was cooled on ice bath. Propargyl bromide,80% in toluene, ca. 9.2 mol/L, 500 µL, and silver triflate (7.7 mg, 30 µmol) were added and the reaction mixture was stirred next 24 h. The reaction was quench by MeOH (500 µL) and the solvent was removed by evaporation. The residue was suspended in H_2O and

insoluble Ag salt was filtered off. The crude mixture was purified C18 reverse phase HPLC (2 mL/min, NUCLEODUR® C18 Pyramid VP 250/21 column, linear gradient B conc. 5% - 7% (0 min to 15 min), 7% - 70% (15min to 30 min); solvent A was H_2O + 0.1% TFA; solvent B was MeCN + 0.1% TFA; flow rate was 2 ml/min at 30 °C with UV detection at 260 nm.) Fractions containing the desired material were combined and removal of solvent by lyophilization. Yield: 0.9 µmol (determined by UV absorbance at 279 nm, $\varepsilon_{279\ nm}$ = 9,894 $Lmol^{-1}cm^{-1}$) of **7** as white foam (7.5 %).

^{1}H NMR (400 MHz, D_2O) δ 8.05 (s, 1H), 5.82 (d, *J* = 5.0 Hz, 1H), 4.82 – 4.75 (m, 2H), 4.36 – 4.28 (m, 3H), 3.15 (t, *J* = 2.6 Hz, 1H), 3.12 – 3.05 (m, 2H), 2.51 – 2.45 (m, 2H), 2.35 – 2.14 (m, 4H). Due to low sample yield, signal was not observed in **^{13}C NMR,**

ESI-MS (*m/z*): $[M]^+$ calcd for $C_{17}H_{24}N_7O_4Se$, 470.1050; found 470.1033.

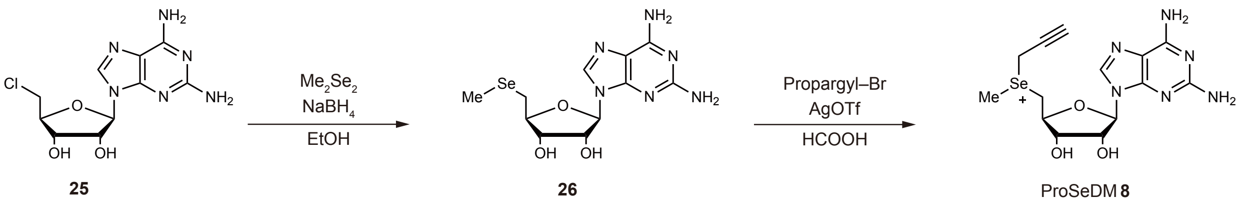

Supplementary Scheme 4. Synthesis of ProSeDM

5′-*Se*-methyl-5′-seleno-2,6-Diaminopurineribose (26)

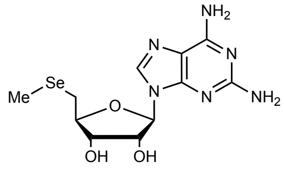

26

Dimethyldiselenid (112 mg, 596 µmol) was activated by sodium borohydride (22 mg, 582 µmol) in *i*PrOH (3 mL). After 30 min, 5′-chloro-2-aminoadenosine (100 mg, 333 µmol) was added and the reaction was proceeded for 15 h. The reaction mixture was diluted by DCM and extracted water. The organic phase was dried Na_2SO_4 and evaporated. The crude product was purified by column chromatography on SiO_2 with DCM / MeOH = 9 / 1. Yield: 72 mg of **26** as white foam (60 %).

^{1}H NMR (400 MHz, D_2O) δ 8.05 (s, 1H), 5.81 (d, *J* = 5.3 Hz, 1H), 4.76 (t, *J* = 5.3 Hz, 1H), 4.29 (t, *J* = 5.3 Hz, 1H), 4.23 (q, *J* = 5.3 Hz, 1H), 2.90– 2.80 (m, 2H), 1.90 (s, 3H). **^{13}C NMR** (100 MHz, D_2O) δ 152.23, 151.65, 151. 149.81, 140.37, 111.60, 87.41, 84.00, 73.03, 72.87, 26.78, 4.43.

ESI-MS (*m/z*): $[M+H]^+$ calcd for $C_{11}H_{17}N_6O_3Se$, 361.0522; found 361.0522.

5′-*Se*-Propargyl-5′-*Se*-methyl-5′-seleno-2,6-Diaminopurineribose (8)

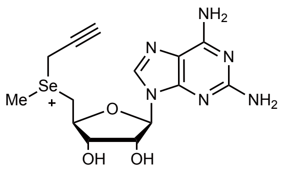

ProSeDM **8**

Compound **26** (20 mg, 56 µmol) was dissolved in formic acid (5 mL) and the solution was cooled on ice bath. Propargyl bromide (80% in toluene, ca. 9.2 mol/L, 2.3 mL) and silver triflate (35 mg, 136 µmol) were added and the reaction mixture was stirred next 24 h. The reaction was quench by MeOH (500 µL) and the solvent was removed by evaporation. The residue was suspended in H_2O and insoluble Ag salt was filtered off. The crude mixture was purified C18 reverse phase HPLC (2 mL/min, NUCLEODUR® C18 Pyramid VP 250/21 column, linear gradient B conc. 5% - 7% (0 min to 15 min), 7% - 70% (15min to 30 min); solvent A was H_2O + 0.1% TFA; solvent B was MeCN + 0.1% TFA; flow rate was 2 ml/min at 30 °C with UV detection at 260 nm.) Fractions containing the desired material were combined and removal of solvent by lyophilization. Yield: 25 µmol (determined by UV absorbance at 279 nm, $\varepsilon_{279\ nm}$ = 9,894 $Lmol^{-1}cm^{-1}$) of **8** as white foam (45 %).

^{1}H NMR (400 MHz, D_2O) δ 7.99 (s, 1H), 5.85 (d, *J* = 5.8 Hz, 1H), 4.76 – 4.72 (m, 1H), 4.56 (t, *J* = 5.8 Hz, 1H), 4.43 – 4.38 (m, 1H), 3.92 (dd, *J* = 8.3 Hz, 2.7 Hz, 2H) 3.71 – 3.69 (m, 2H), 2.98 (t, *J* = 2.7 Hz, 1H) 2.68 (s, 3H). **^{13}C NMR** (100 MHz, D_2O) δ 152.11, 151.54, 149.72, 140.74, 111.19, 89.17, 79.83, 79.49, 73.42, 72.97, 70.95, 41.21, 26.39, 18.79.

ESI-MS (*m/z*): $[M]^+$ calcd for $C_{14}H_{19}N_6O_3Se^+$, 399.0678; found 399.0669.

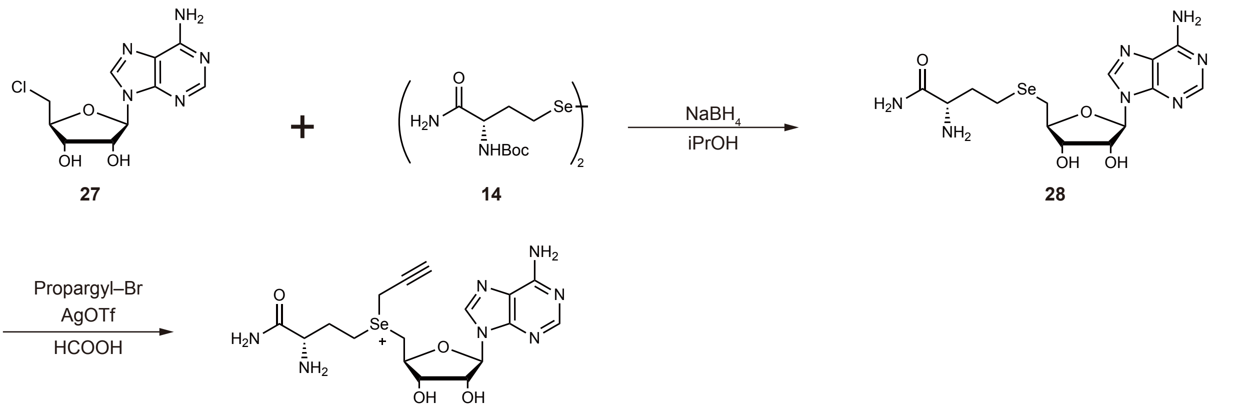
ProSeAMA **9**

Supplementary Scheme 5. Synthesis of ProSeAMA

***Se*-2,6-adenosyl-L-selenomethionineamide (28)**

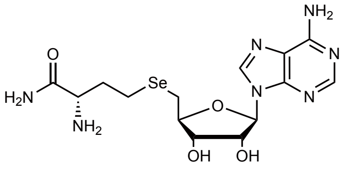
28

Compound **14 (**392 mg, 700 µmol**)** was activated by sodium borohydride (26 mg, 700 µmol) in *i*PrOH (3.5 mL). After 30 min, 5′-chloro-adenosine[3] (100 mg, 350 µmol) was added, and the reaction was proceeded for 1 h with heating under reflux. The reaction mixture was diluted by DCM and extracted with water. The aqueous phase was dried by evaporation and the residue was dissolved in formic acid (1 mL). After overnight Boc-deprotection, the solvent was removed and the crude mixture was purified by C18 reverse phase HPLC (2 mL/min, NUCLEODUR® C18 Pyramid VP 250/21 column, linear gradient B conc. 5% - 7% (0 min to 15 min), 7% - 70% (15min to 30 min); solvent A was H_2O + 0.1% TFA; solvent B was MeCN + 0.1% TFA; flow rate was 2 ml/min at 30 °C with UV detection at 260 nm.) Fractions containing the desired material were combined and removal of solvent by lyophilization. Yield: 37 µmol (determined by UV absorbance at 260 nm, $\varepsilon_{260\ nm}$ = 15,400 $Lmol^{-1}cm^{-1}$) of **28** as white foam (11%).

^{1}H NMR (400 MHz, D_2O) δ 8.41 (s, 1H) 8.34 (s, 1H), 6.03 (d, *J* = 4.7 Hz, 1H), 4.76 (t, *J* = 4.7 Hz, 1H), 4.32 (t, *J* = 5.1 Hz, 1H), 4.28 – 4.23 (m, 1H), 3.96 (t, *J* = 6.5 Hz 1H), 3.01 – 2.99 (m, 2H), 2.58 – 2.53 (m, 2H), 2.14 – 2.06 (m, 2H). **^{13}C NMR** (100 MHz, D_2O) δ 171.31, 149.94, 148.27, 144.49, 142.80, 114.80, 88.32, 83.90, 73.62, 72.82, 52.79, 31.67, 25.42, 17.90.

ESI-MS (*m/z*): $[M+H]^+$ calcd for $C_{14}H_{22}N_7O_4Se$, 432.0893; found 432.0892.

***Se*-Propargyl-*Se*-adenosyl-L-selenomethionineamide (9)**

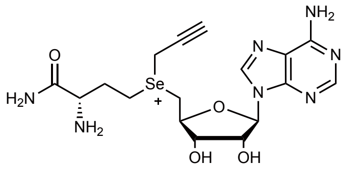

ProSeAMA **9**

Compound **28** (15 mg, 35 μmol) was dissolved in formic acid (3.5 mL) and the solution was cooled on ice bath. Propargyl bromide (80% in toluene, ca. 9.2 mol/L, 1.4 mL) and silver triflate (23 mg, 88 μmol) were added and the reaction mixture was stirred next 24 h. The reaction was quench by MeOH (500 μL) and the solvent was removed by evaporation. The residue was suspended in H_2O and insoluble Ag salt was filtered off. The crude mixture was purified C18 reverse phase HPLC (2 mL/min, NUCLEODUR® C18 Pyramid VP 250/21 column, linear gradient B conc. 5% - 7% (0 min to 15 min), 7% - 70% (15min to 30 min); solvent A was H_2O + 0.1% TFA; solvent B was MeCN + 0.1% TFA; flow rate was 2 ml/min at 30 °C with UV detection at 260 nm.) Fractions containing the desired material were combined and removal of solvent by lyophilization. Yield: 8.7 μmol (determined by UV absorbance at 260 nm, $\varepsilon_{260\ nm}$ = 15,400 $Lmol^{-1}cm^{-1}$) of **9** as white foam (25 %).

^{1}H NMR (400 MHz, D_2O) δ 8.37 (s, 1H) 8.35 (s, 1H), 6.08 – 6.05 (m, 1H), 4.76 – 4.73 (m, 1H), 4.57 – 4.43 (m, 2H), 4.16 – 3.77 (m, 5H), 3.42 – 3.34 (m, 2H), 3.15 – 3.04 (m, 1H), 2.38 – 2.26 (m, 2H).

^{13}C NMR (100 MHz, D_2O) δ 163.12, 151.59, 147.95, 144.82, 142.98, 114.82, 89.71, 80.78, 73.42, 73.33, 70.66, 51.76, 40.80, 32.98, 25.88.

ESI-MS (*m/z*): $[M]^+$ calcd for $C_{17}H_{24}N_7O_4Se$, 470.1050; found 470.1040.

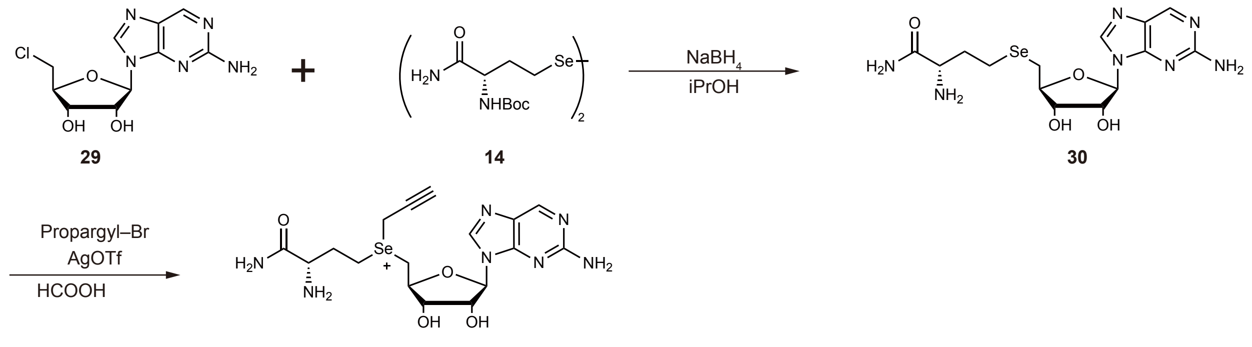

ProSeAPMA **10**

Supplementary Scheme 6. Synthesis of ProSeAPMA

***Se*-2-aminopurineribosyl -L-selenomethionineamide (30)**

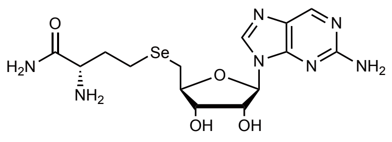

30

Compound **14 (**100 mg, 180 μmol**)** was activated by sodium borohydride (7 mg, 185 μmol) in *i*PrOH (1 mL). After 30 min, 5′-chloro-2-aminopurineribose[4] (25mg, 88 μmol) was added, and the reaction was proceeded for 1 h with heating at 30 °C. The reaction mixture was diluted by DCM and extracted with water. The aqueous phase was dried by evaporation and the residue was dissolved in formic acid (1 mL). After overnight Boc-deprotection, the solvent was removed and the crude mixture was purified by C18 reverse phase HPLC (2 mL/min, NUCLEODUR® C18 Pyramid VP 250/21 column, linear gradient B conc. 5% - 7% (0 min to 15 min), 7% - 70% (15min to 30 min); solvent A was H_2O + 0.1% TFA; solvent B was MeCN + 0.1% TFA; flow rate was 2 ml/min at 30 °C with UV detection at 260 nm.) Fractions containing the desired material were combined and removal of solvent by lyophilization. Yield: 13 μmol (determined by UV absorbance at 260 nm, $\varepsilon_{260\ nm}$ = 1,000 $Lmol^{-1}cm^{-1}$) of **30** as white foam (15%).

^{1}H NMR (400 MHz, D_2O) δ 8.64 (s, 1H), 8.48 (s, 1H), 5.93 (d, *J* = 5.0 Hz, 1H), 4.78 (t, *J* = 5.0 Hz, 1H), 4.32 (t, *J* = 5.0 Hz, 1H), 4.24 – 4.20 (m, 1H), 3.96 (t, *J* = 6.5 Hz 1H), 3.00 – 2.89 (m, 2H), 2.59 – 2.52 (m, 2H), 2.14 – 2.07 (m, 2H). **^{13}C NMR** (100 MHz, D_2O) δ 171.31, 157.17, 153.66, 148.77, 138.09, 126.57, 88.12, 83.77, 73.90, 72.86, 52.79, 31.66, 25.42, 17.86.

ESI-MS (*m/z*): $[M+H]^+$ calcd for $C_{14}H_{22}N_7O_4Se$, 432.0893; found 432.0895.

***Se*-Propargyl-*Se*-2-aminopurineribosyl-L-selenomethionineamide (10)**

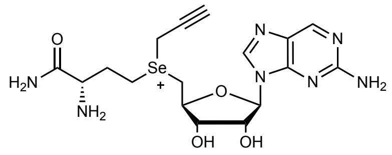

ProSeAPMA **10**

Compound **30** (5 mg, 12 μmol) was dissolved in formic acid (1.2 mL) and the solution was cooled on ice bath. Propargyl bromide (80% in toluene, ca. 9.2 mol/L, 483 μL) and silver triflate (7.5 mg, 29 μmol) were added and the reaction mixture was stirred next 24 h. The reaction was quench by MeOH (500 μL) and the solvent was removed by evaporation. The residue was suspended in H_2O and insoluble Ag salt was filtered off. The crude mixture was purified C18 reverse phase HPLC (2 mL/min, NUCLEODUR® C18 Pyramid VP 250/21 column, linear gradient B conc. 5% - 7% (0 min to 15 min), 7% - 70% (15min to 30 min); solvent A was H_2O + 0.1% TFA; solvent B was MeCN + 0.1% TFA; flow rate was 2 ml/min at 30 °C with UV detection at 300 nm.) Fractions containing the desired material were combined and removal of solvent by lyophilization. Yield: 1.6 μmol (determined by UV absorbance at 260 nm, $\varepsilon_{260\ nm}$ = 1,000 $Lmol^{-1}cm^{-1}$) of **10** as white foam (13 %).

^{1}H NMR (400 MHz, D_2O) δ 8.66 (s, 1H), 8.43 (s, 1H), 5.97 – 5.95 (m, 1H), 4.74 – 4.71 (m, 1H), 4.62 – 4.54 (m, 2H), 4.46 – 4.40 (m, 2H), 4.17 – 3.90 (m, 3H) 3.85 – 3.75 (m, 2H), 3.43 – 3.31 (m, 2H), 3.14 – 3.04 (m, 1H), 2.38 – 2.22 (m, 2H). **^{13}C NMR** (100 MHz, D_2O) δ 169.90, 156.77, 153.78, 148.55, 138.69, 126.66, 89.73, 80.71, 79.16, 73.42, 73.04, 70.65, 51.76, 40.69, 32.96, 25.98, 25.85.

ESI-MS (*m/z*): $[M]^+$ calcd for $C_{17}H_{24}N_7O_4Se$, 470.1050; found 470.1051.

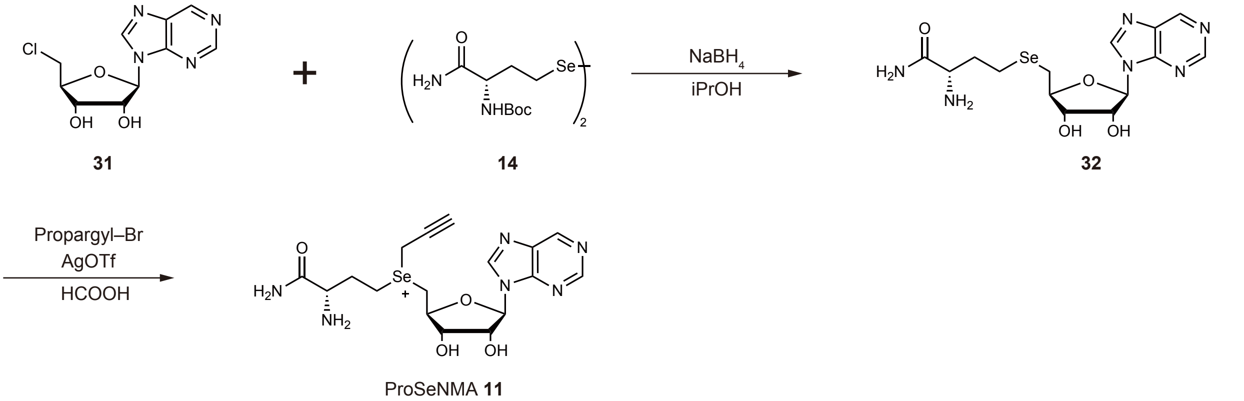

Supplementary Scheme 7. Synthesis of ProSeNMA

Se-purineribosyl -L-selenomethionineamide (32)

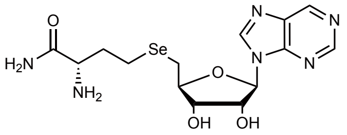

32

Compound **14** **(**42 mg, 75 µmol**)** was activated by sodium borohydride (3 mg, 79 µmol) in *i*PrOH (1 mL). After 30 min, 5′-chloro-purineribose[5] (10 mg, 37 µmol) was added, and the reaction was proceeded for 1 h with heating at 30 °C The reaction mixture was diluted by DCM and extracted with water. The aqueous phase was dried by evaporation and the residue was dissolved in formic acid (1 mL). After overnight Boc-deprotection, the solvent was removed and the crude mixture was purified by C18 reverse phase HPLC (2 mL/min, NUCLEODUR® C18 Pyramid VP 250/21 column, linear gradient B conc. 5% - 7% (0 min to 15 min), 7% - 70% (15min to 30 min); solvent A was H_2O + 0.1% TFA; solvent B was MeCN + 0.1% TFA; flow rate was 2 ml/min at 30 °C with UV detection at 260 nm.) Fractions containing the desired material were combined and removal of solvent by lyophilization. Yield: 9 µmol (determined by UV absorbance at 245 nm, $\varepsilon_{245\ nm}$ = 7,000 $Lmol^{-1}cm^{-1}$) of **32** as white foam (24%).

^{1}H NMR (400 MHz, D_2O) δ 9.32 (s, 1H), 9.14 (s, 1H), 8.86 (s, 1H), 6.19 (d, *J* = 4.5 Hz, 1H), 4.84 (t, *J* = 4.5 Hz, 1H), 4.36 (t, *J* = 4.5 Hz, 1H), 4.32 – 4.27 (m, 1H), 3.96 (t, *J* = 6.5 Hz 1H), 3.04 – 2.93 (m, 2H), 2.58 – 2.53 (m, 2H), 2.14 – 2.07 (m, 2H). **^{13}C NMR** (100 MHz, D_2O) δ 171.29, 152.96, 149.02, 148.38, 142.99, 133.14, 88.68, 83.95, 73.53, 72.88, 52.78, 31.66, 25.38, 17.90.

ESI-MS (*m/z*): $[M+H]^+$ calcd for $C_{14}H_{21}N_6O_4Se$, 417.0784; found 417.0782.

***Se*-Propargyl-*Se*-2-purineribosyl-L-selenomethionineamide (11)**

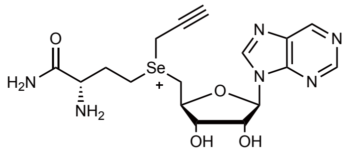

ProSeNMA **11**

Compound **32** (3.3 mg, 8 µmol) was dissolved in formic acid (1 mL) and the solution was cooled on ice bath. Propargyl bromide (80% in toluene, ca. 9.2 mol/L, 333 µL), and silver triflate (5.1 mg, 20 µmol) were added and the reaction mixture was stirred next 24 h. The reaction was quench by MeOH (500 µL) and the solvent was removed by evaporation. The residue was suspended in H_2O and insoluble Ag salt was filtered off. The crude mixture was purified C18 reverse phase HPLC (2 mL/min, NUCLEODUR® C18 Pyramid VP 250/21 column, linear gradient B conc. 5% - 7% (0 min to 15 min), 7% - 70% (15min to 30 min); solvent A was H_2O + 0.1% TFA; solvent B was MeCN + 0.1% TFA; flow rate was 2 ml/min at 30 °C with UV detection at 260 nm.) Fractions containing the desired material were combined and removal of solvent by lyophilization. Yield: 2.4 µmol (determined by UV absorbance at 245 nm, $\varepsilon_{245\ nm} = 7{,}000\ Lmol^{-1}cm^{-1}$) of **11** as white foam (30 %).

^{1}H NMR (400 MHz, D_2O) δ 9.22 (s, 1H), 9.04 (s, 1H), 8.69 (s, 1H), 6.19 – 6.15 (m, 1H), 4.85 – 4.80 (m, 1H), 4.68 – 4.59 (m, 1H), 4.53 – 4.48 (m, 1H), 4.14 – 3.84 (m, 5H), 3.39 – 3.31 (m, 2H) 3.16 – 2.95 (m, 1H), 2.35 – 2.20 (m, 2H). **^{13}C NMR** (100 MHz, D_2O) δ 170.41, 151.05, 150.24, 147.70, 145.62, 132.81, 89.86, 80.68, 79.28, 73.44, 73.20, 51.73, 32.98, 31.61, 25.89, 25.86.

ESI-MS (*m/z*): $[M]^+$ calcd for $C_{17}H_{23}N_6O_4Se^+$, 455.0941; found 455.0903.

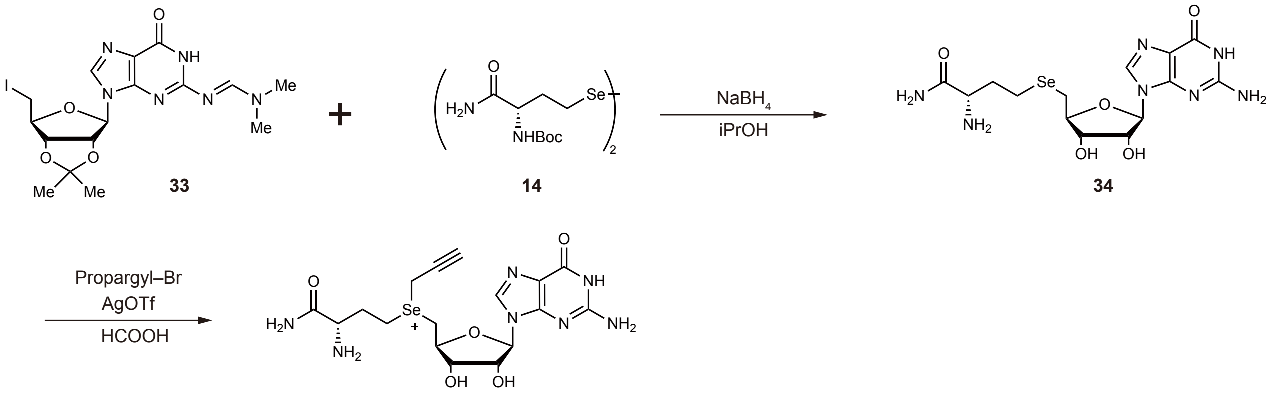

ProSeGMA **12**

Supplementary Scheme 8. Synthesis of ProSeGMA

***Se*-guanosyl -L-selenomethionineamide (34)**

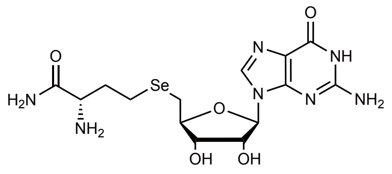
34

Compound **14** (223 mg, 398 μmol) was activated by sodium borohydride (15 mg, 397 μmol) in *i*PrOH (2 mL). After 30 min, guanosine derivatives **33**[6] (100 mg, 205 μmol) was added, and the reaction was proceeded for 1 h with heating at 30 °C. The reaction mixture was diluted by DCM and extracted with water. The organic phase was dried by evaporation and the residue was dissolved in 28% NH_4 aq. (2 mL) for N2-DMF-deprotection. After 2h, ammonia was removed by evaporation and formic acid (1 mL) was added. After overnight Boc-deprotection, the solvent was removed and the crude mixture was purified by C18 reverse phase HPLC (2 mL/min, NUCLEODUR® C18 Pyramid VP 250/21 column, linear gradient B conc. 5% - 7% (0 min to 15 min), 7% - 70% (15min to 30 min); solvent A was H_2O + 0.1% TFA; solvent B was MeCN + 0.1% TFA; flow rate was 2 ml/min at 30 °C with UV detection at 260 nm.) Fractions containing the desired material were combined and removal of solvent by lyophilization. Yield: 18 μmol (determined by UV absorbance at 260 nm, $\varepsilon_{260\ nm}$ = 11,500 $Lmol^{-1}cm^{-1}$) of **34** as white foam (9%).

^{1}H NMR (400 MHz, D_2O) δ 8.69 (s, 1H), 5.88 (d, *J* = 5.0 Hz, 1H), 4.72 (t, *J* = 5.0 Hz, 1H), 4.27 – 4.22 (m, 2H), 3.96 (t, *J* = 6.5 Hz 1H), 2.97 – 2.94 (m, 2H), 2.62 – 2.50 (m, 2H), 2.13 – 2.07 (m, 2H).

^{13}C NMR (100 MHz, D_2O) δ 171.30, 155.91, 154.99, 149.98, 136.31, 110.16, 89.31, 83.93, 73.36, 72.73, 52.79, 31.68, 25.30, 17.96.

ESI-MS (*m/z*): $[M+H]^+$ calcd for $C_{14}H_{22}N_7O_4Se$, 432.0893; found 432.0892.

***Se*-Propargyl-*Se*-2-purineribosyl-L-selenomethionineamide (12)**

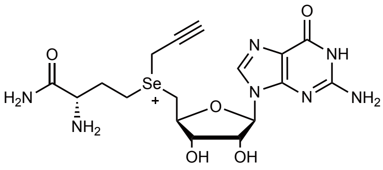

ProSeGMA **12**

Compound **34** (6 mg, 15 µmol) was dissolved in formic acid (1.5 mL) and the solution was cooled on ice bath. Propargyl bromide(80% in toluene, ca. 9.2 mol/L, 625 µL) and silver triflate (9.6 mg, 37 µmol) were added and the reaction mixture was stirred next 24 h. The reaction was quench by MeOH (500 µL) and the solvent was removed by evaporation. The residue was suspended in H_2O and insoluble Ag salt was filtered off. The crude mixture was purified C18 reverse phase HPLC (2 mL/min, NUCLEODUR® C18 Pyramid VP 250/21 column, linear gradient B conc. 5% - 7% (0 min to 15 min), 7% - 70% (15min to 30 min); solvent A was H_2O + 0.1% TFA; solvent B was MeCN + 0.1% TFA; flow rate was 2 ml/min at 30 °C with UV detection at 260 nm.) Fractions containing the desired material were combined and removal of solvent by lyophilization. Yield: 1.5 µmol (determined by UV absorbance at 260 nm, $\varepsilon_{260\ nm}$ = 11,500 $Lmol^{-1}cm^{-1}$) of **12** as white foam (10 %).

^{1}H NMR (400 MHz, D_2O) δ 8.45 (s, 1H), 5.91 (m, 1H), 4.77 – 4.74 (m, 1H), 4.63 – 4.55 (m, 2H), 4.47 – 4.42 (m, 1H), 4.16 – 3.82 (m, 5H), 3.42 – 3.29 (m, 2H), 3.14 – 3.06 (m, 1H), 2.34 – 2.23 (m, 2H).

^{13}C NMR (100 MHz, D_2O) δ 169.80, 160.87, 155.15, 154.60, 137.23, 111.94, 90.52, 80.67, 79.42, 73.30, 73.04, 70.52, 51.76, 41.01, 33.02, 25.96, 25.84.

ESI-MS (*m/z*): $[M]^+$ calcd for $C_{17}H_{24}N_7O_5Se^+$, 486.0999; found 486.0999.

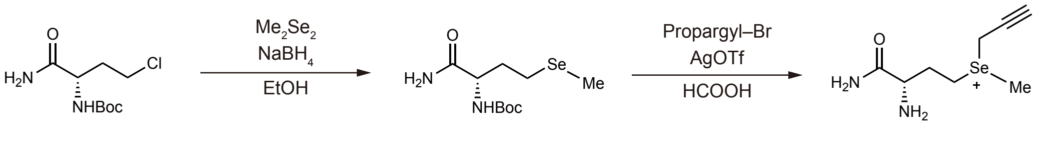

35 **36** ProSeMA **13**

Supplementary Scheme 9. Synthesis of ProSeMA

N-Boc-L-selenomethionineamide (36)

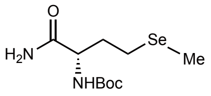

36

Dimethyldiselenid **(**120 µL, 1.3 mmol**)** was activated by sodium borohydride (240 mg, 6.3 mmol) in EtOH (12 mL). After 30 min, *N*-Boc-2-amino-4-chlorobutanamide **35**[1] (300 mg, 1.3 mmol) was added and the reaction was proceeded for 15 h. The reaction mixture was diluted by DCM and extracted water. The organic phase was dried Na_2SO_4 and evaporated. The crude product was purified by column chromatography on SiO_2 with DCM / MeOH = 95 / 5. Yield: 153 mg of **39** as white foam (40 %).
^{1}H NMR (400 MHz, DMSO-d6) δ 7.25 (s, 1H), 6.99 (s, 1H), 6.87 (d, *J* = 8.3 Hz, 1H), 3.92 (td, *J* = 8.3, 4.7 Hz, 1H) 2.54–2.40 (m, 2H), 1.94 (s, 3H),1.90–1.78 (m, 2H), 1.38 (s, 9H). **^{13}C NMR** (100 MHz, DMSO) δ 174.18, 155.86, 78.46, 54.75, 33.21, 28.66, 21.25, 3.96.
ESI-MS (*m/z*): $[M+Na]^+$ calcd for $C_{10}H_{20}N_2NaO_3Se$ 319.0531; found 319.0521.

Se-Propargyl-selenomethionineamide (13)

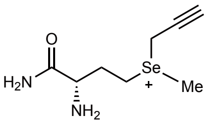

ProSeMA **13**

Compound **36** (20 mg, 68 µmol) was dissolved in formic acid (1 mL) and the solution was cooled on ice bath. Propargyl bromide (80% in toluene, ca. 9.2 mol/L, 280 µL) and silver triflate (45 mg, 176 µmol) were added and the reaction mixture was stirred next 24 h. The reaction was quench by MeOH (500 µL) and the solvent was removed by evaporation. The residue was suspended in H_2O and insoluble Ag salt was filtered off. The crude mixture was purified C18 reverse phase HPLC (2 mL/min, NUCLEODUR® C18 Pyramid VP 250/21 column, linear gradient B conc. 5% - 7% (0 min to 15 min), 7% - 70% (15min to 30 min); solvent A was H_2O + 0.1% TFA; solvent B was MeCN + 0.1% TFA; flow rate was 2 ml/min at 30 °C with UV detection at 220 nm.) Fractions containing the desired

material were combined and removal of solvent by lyophilization. Yield: 6 mg, 26 μmol of **13** as slightly yellow foam (38 %).

^{1}H NMR (400 MHz, D_2O) δ 4.11 – 4.04 (m, 3H), 3.34 – 3.28 (m, 2H), 3.10 – 3.09 (m, 1H), 2.73 (s, 3H), 2.40 – 2.28 (m, 2H). **^{13}C NMR** (100 MHz, D_2O) δ 170.00, 80.23, 70.64, 51.83, 32.98, 25.89 25.30, 18.63.

ESI-MS (*m/z*): $[M]^+$ calcd for $C_8H_{15}N_2OSe^+$, 235.0344; found 235.0344.

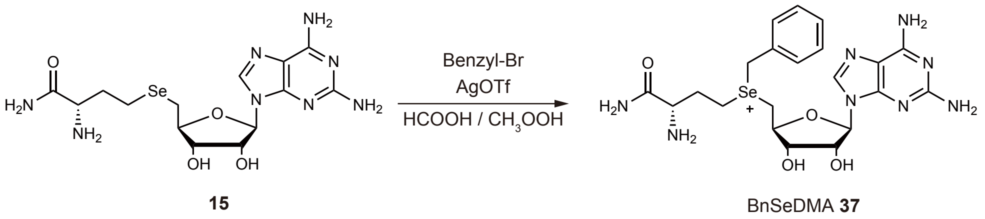

Supplementary Scheme 10. Synthesis of BnSeDMA

Benzylic *Se*-2,6-diaminopurineribosyl-L-selenomethionineamide (37)

Compound **15** (4.5 mg, 10 μmol) was dissolved in acetic acid / formic acid = 1/1 (1 mL) and the solution was cooled in an ice bath. Benzyl bromide (119 μL, 1 mmol) and silver triflate (13 mg, 50 μmol) were added and the reaction mixture was stirred for 15 h. The reaction was quenched by MeOH (500 μL) and the solvent was removed by evaporation. The residue was suspended in H_2O and insoluble Ag salt was filtered off. The crude mixture was purified by C18 reverse phase HPLC (2 mL/min, NUCLEODUR® C18 Pyramid VP 250/21 column, linear gradient B conc. 5% - 7% (0 min to 15 min), 7% - 70% (15min to 30 min); solvent A was H_2O + 0.1% TFA; solvent B was MeCN + 0.1% TFA; flow rate was 2 ml/min at 30 °C with UV detection at 260 nm. Fractions containing the desired material were combined and the solvent was removed by lyophilization. During the purification and lyophilization, some portion of the **37** was decomposed via intramolecular cyclization reactions.

Yield: 240 nmol (determined by UV absorbance at 279 nm, $\varepsilon_{279\ nm}$ = 9,894 $Lmol^{-1}cm^{-1}$) of **37** as white foam (2.4 %) with 72 nmol 5′-Benzylseleno-2-aminoadenosine (analyzed by signal ration of 1′H on diaminopurine).

^{1}H NMR (400 MHz, D_2O) δ 7.91 – 7.89 (m, 1H), 7.31 –7.13 (m, 5H) 5.76 – 5.75 (m, 1H), 4.68 – 4.47 (m, 4H), 4.34 – 4.28 (m, 1H), 4.02 – 3.90 (m, 2H), 3.74 – 3.65 (m, 2H), 3.31 – 3.16 (m, 2H), 2.34 – 2.19 (m, 2H). Due to low sample yield, signal was not observed in **^{13}C NMR.**

ESI-MS (*m/z*): $[M]^+$ calcd for $C_{21}H_{29}N_8O_4Se^+$, 537.1471; found 537.1997.

NMR spectra

Compound 15 ¹HNMR

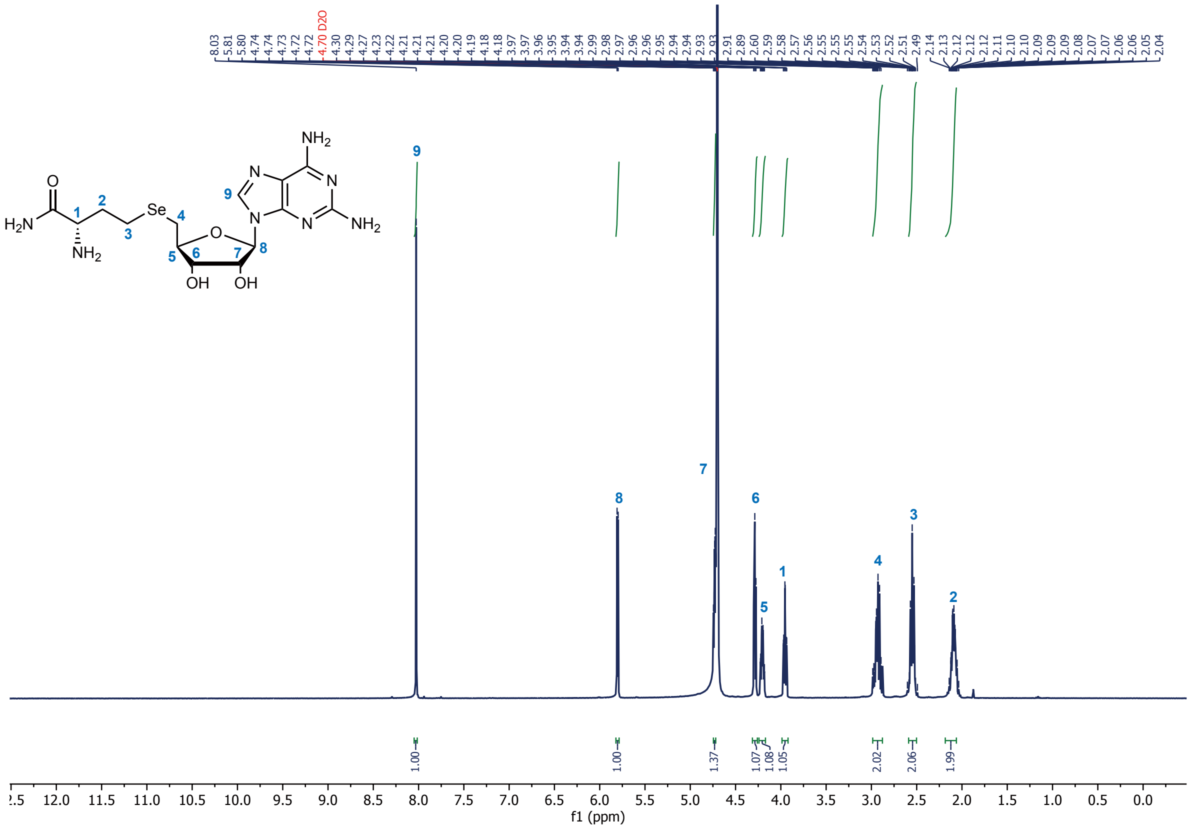

Compound 15 ¹³CNMR

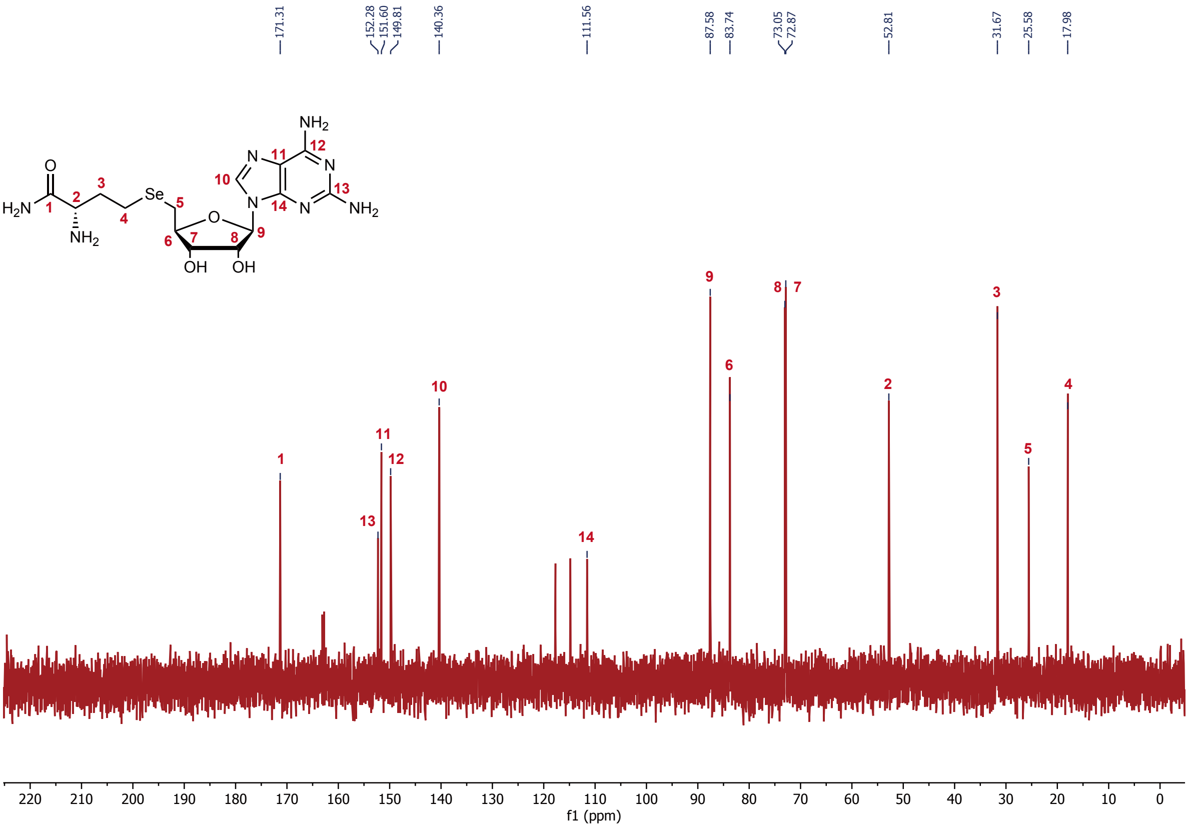

Compound 17 ¹HNMR

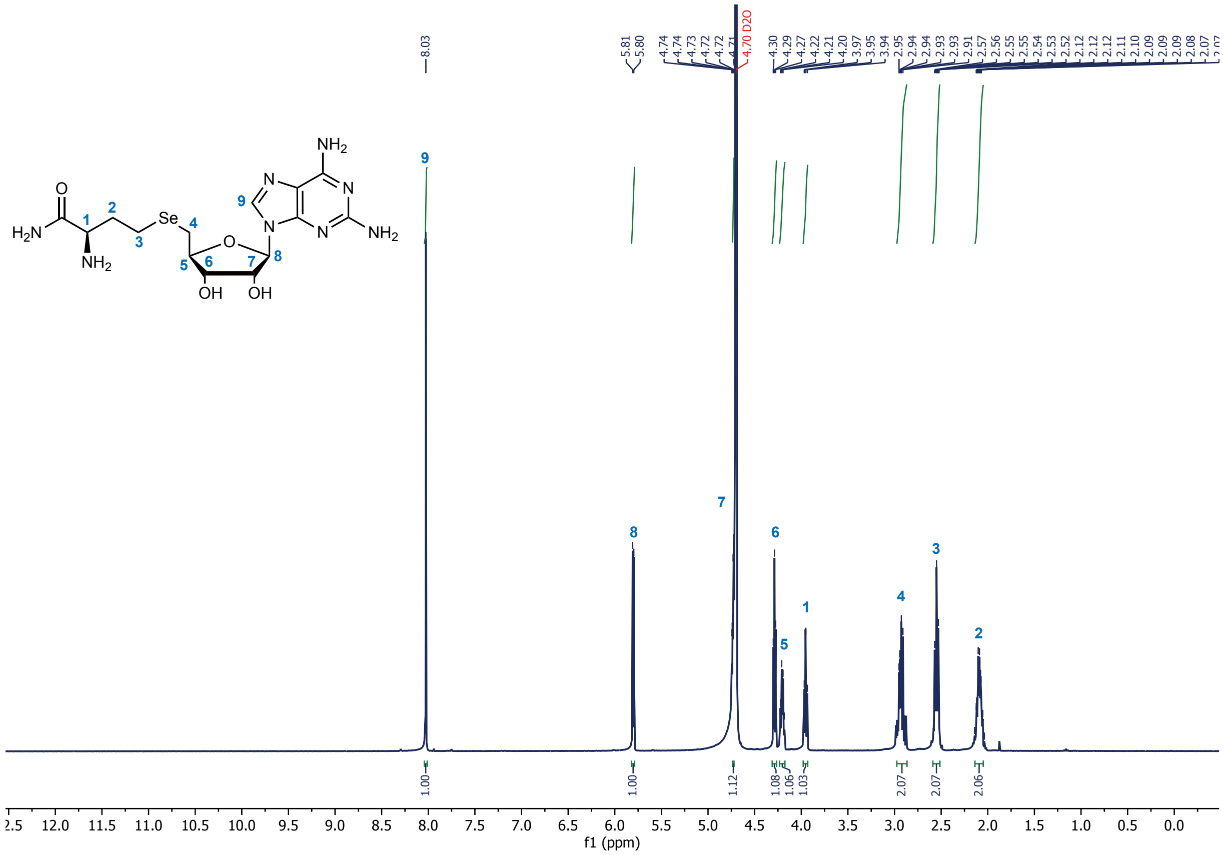

Compound 17 ¹³CNMR

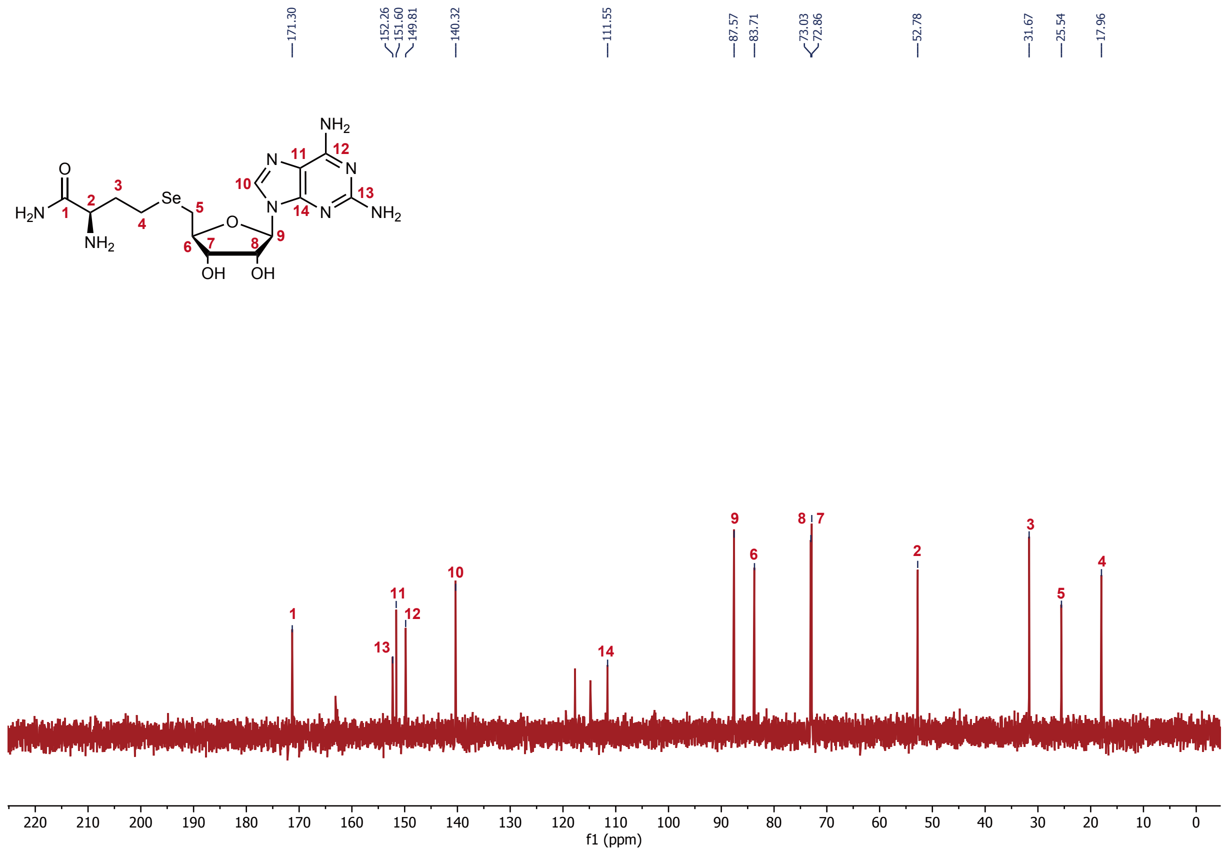

Compound 1S ^{1}HNMR

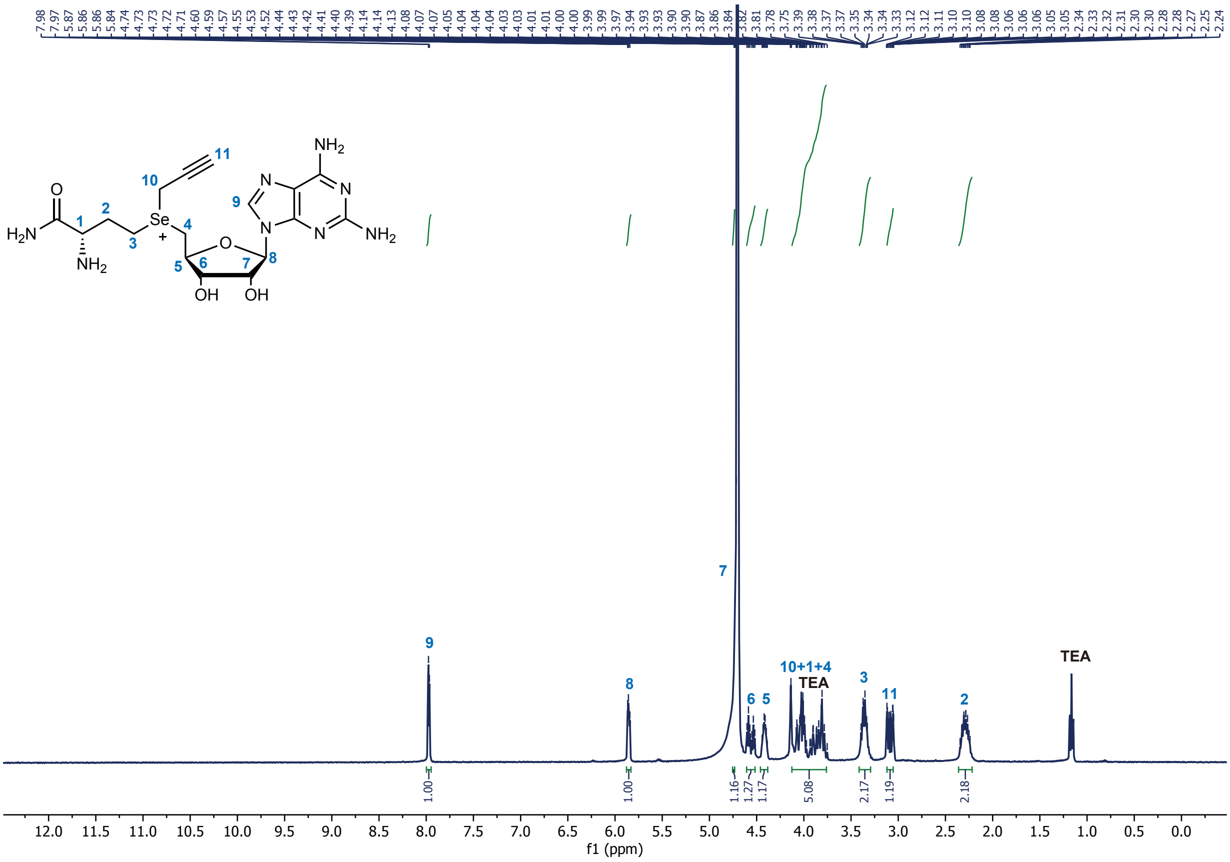

Compound 1S ^{13}CNMR

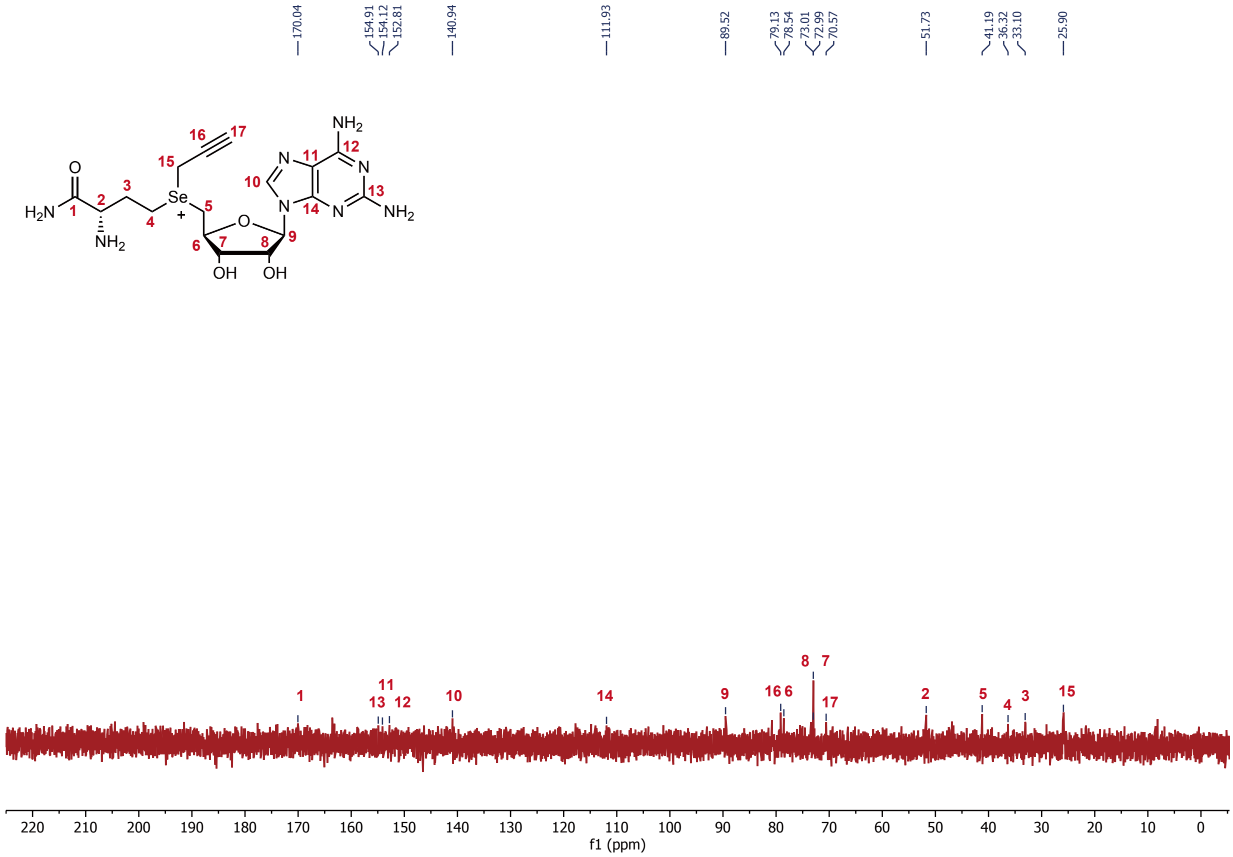

Compound 1R ¹HNMR

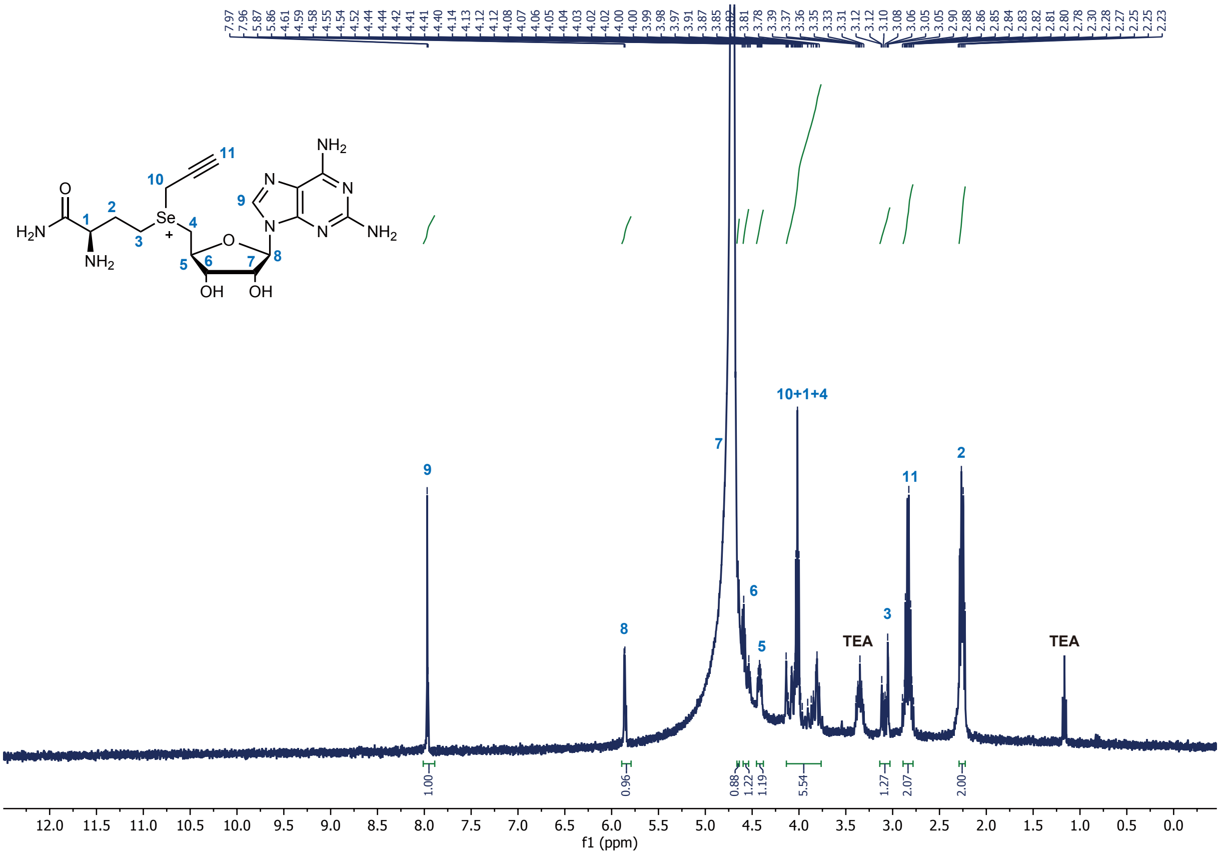

Compound 19 ¹HNMR

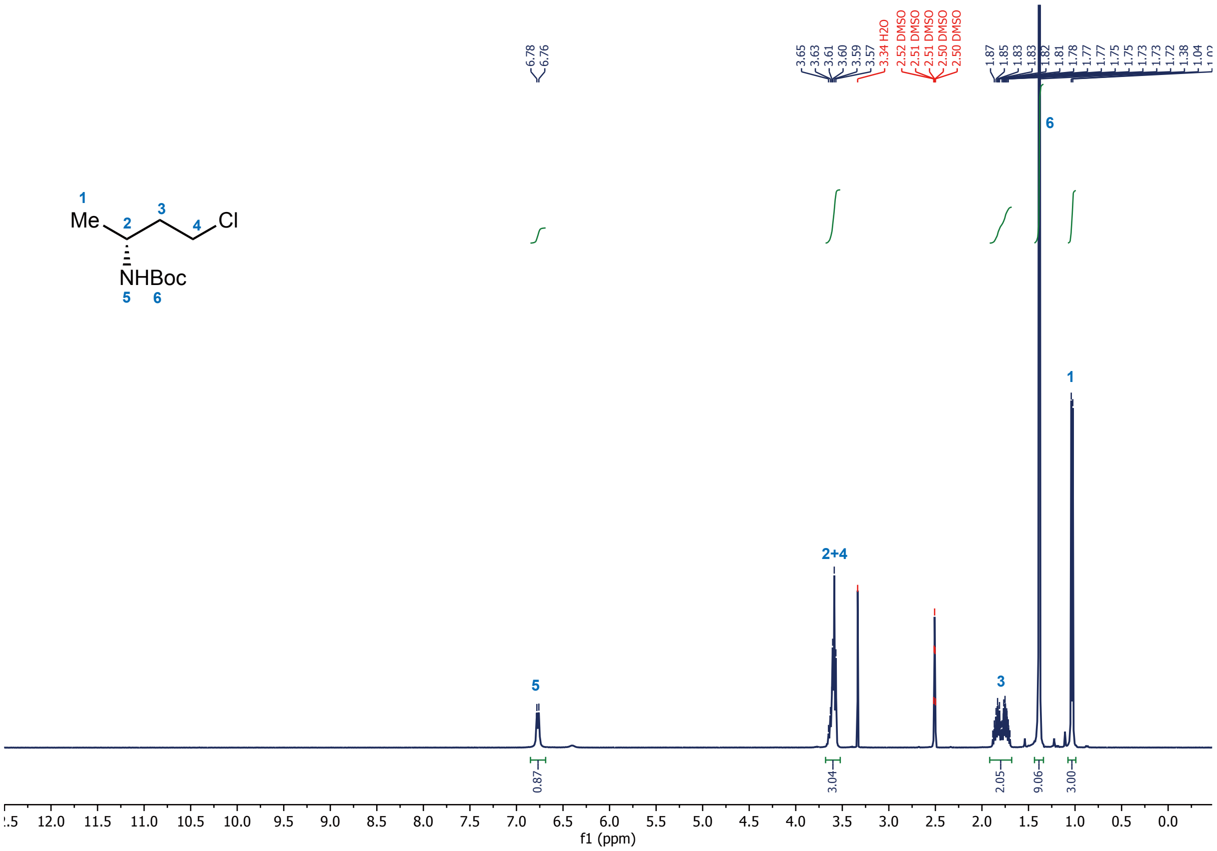

Compound 19 ¹³CNMR

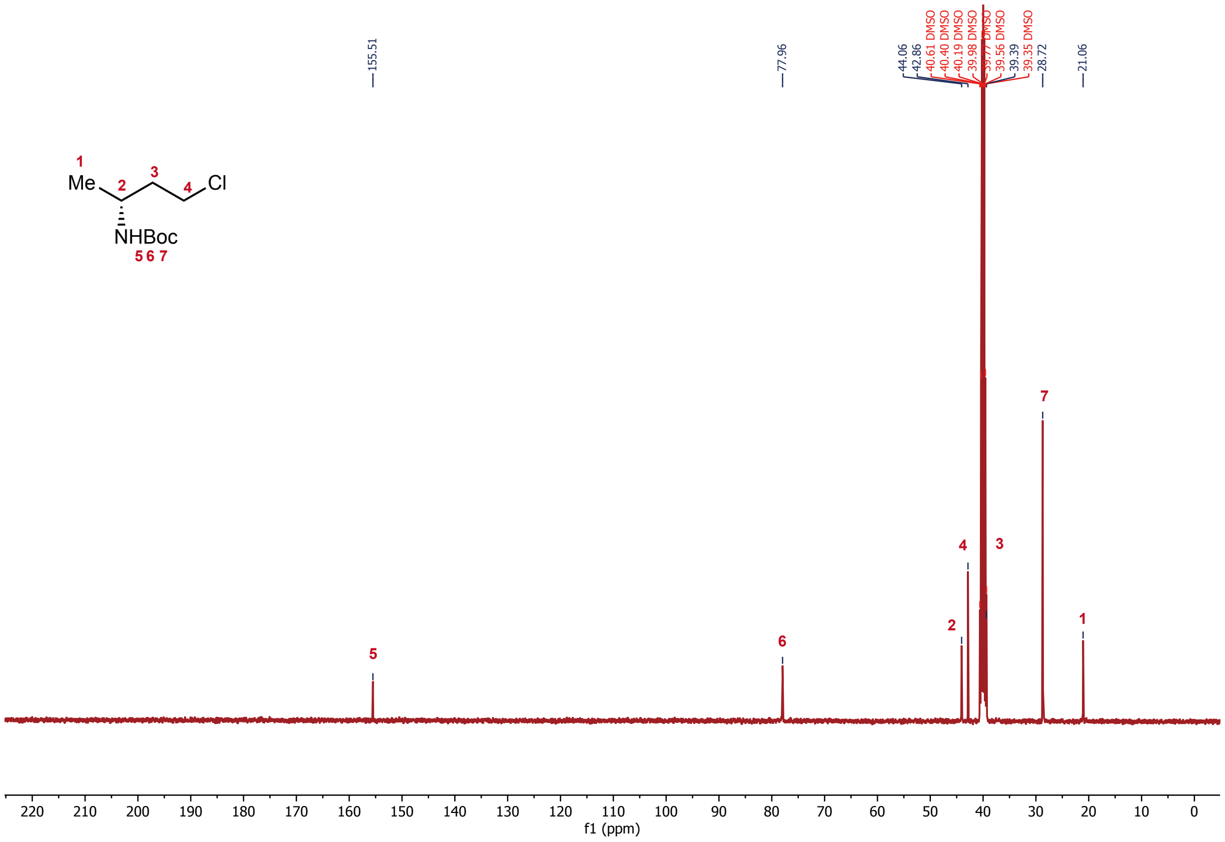

Compound 20 ^{1}HNMR

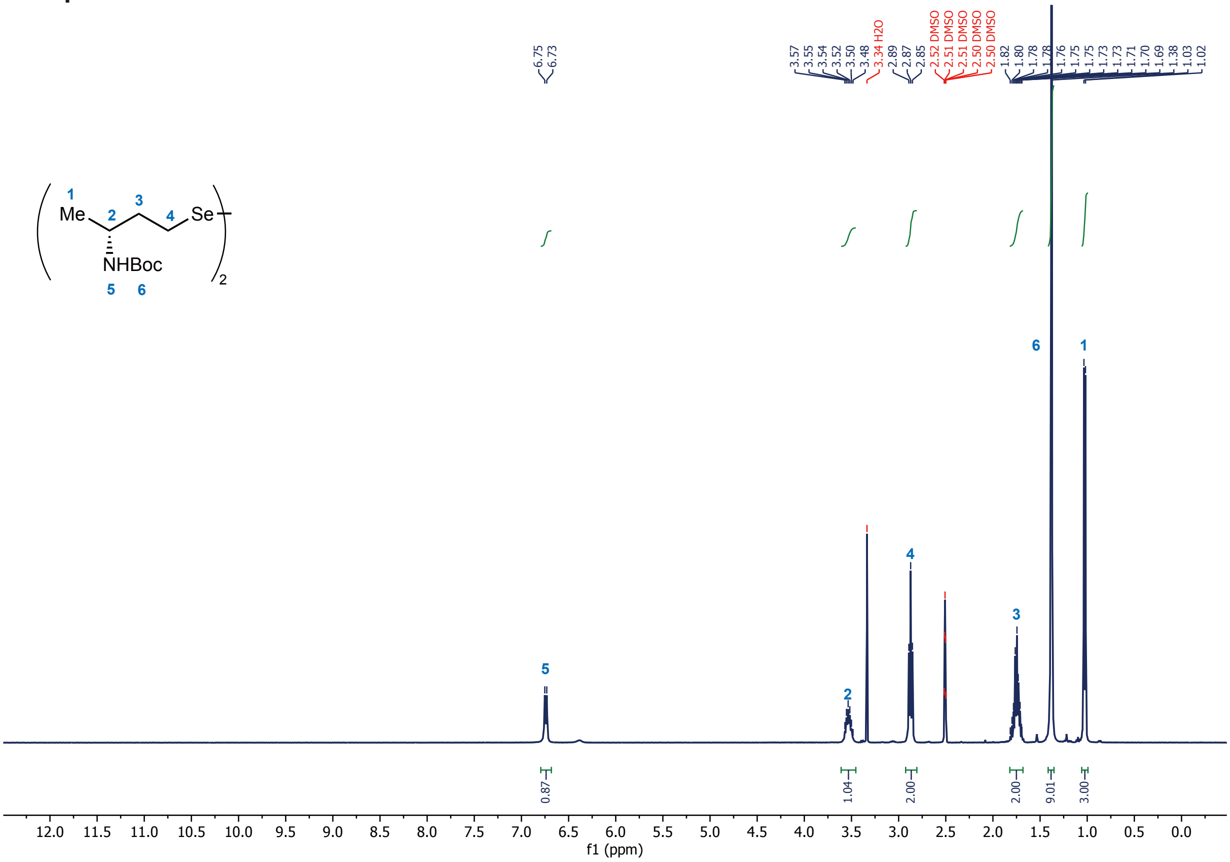

Compound 20 ^{13}CNMR

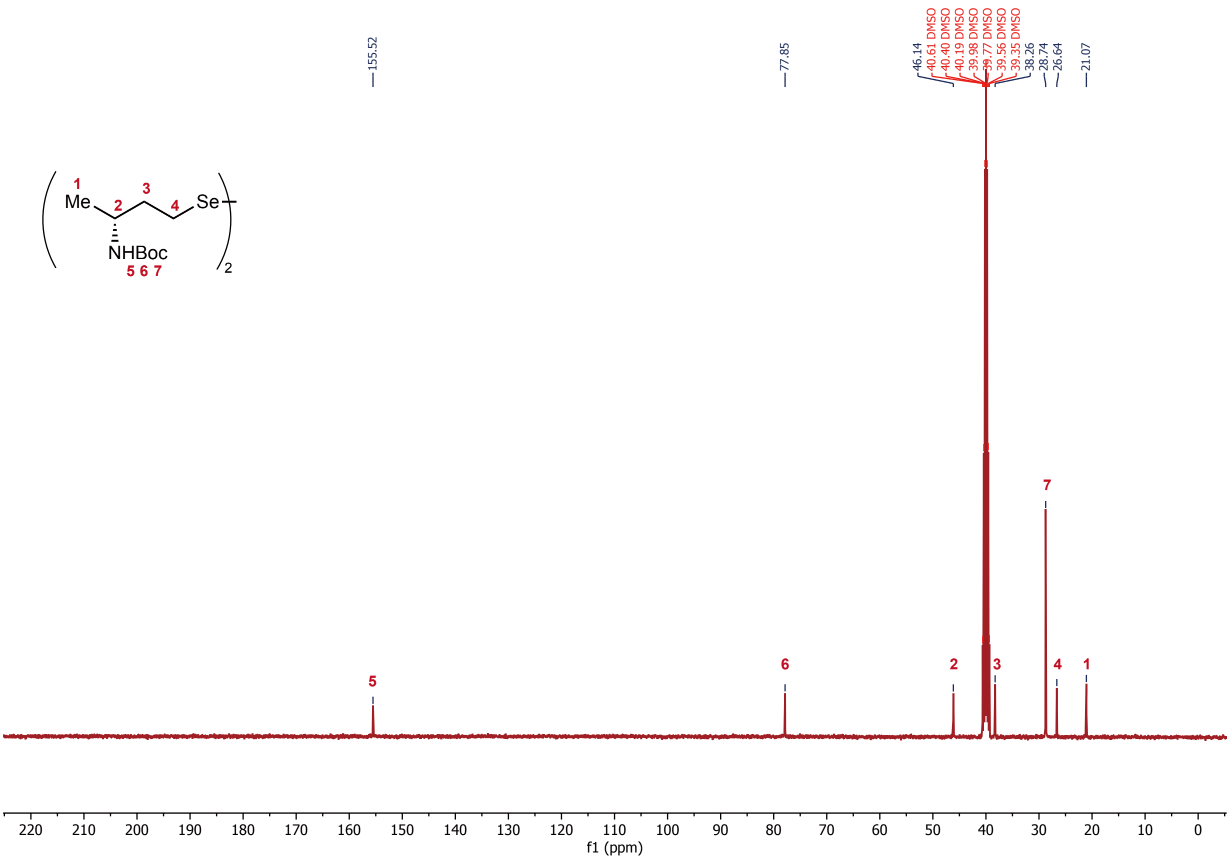

Compound 21 ¹HNMR

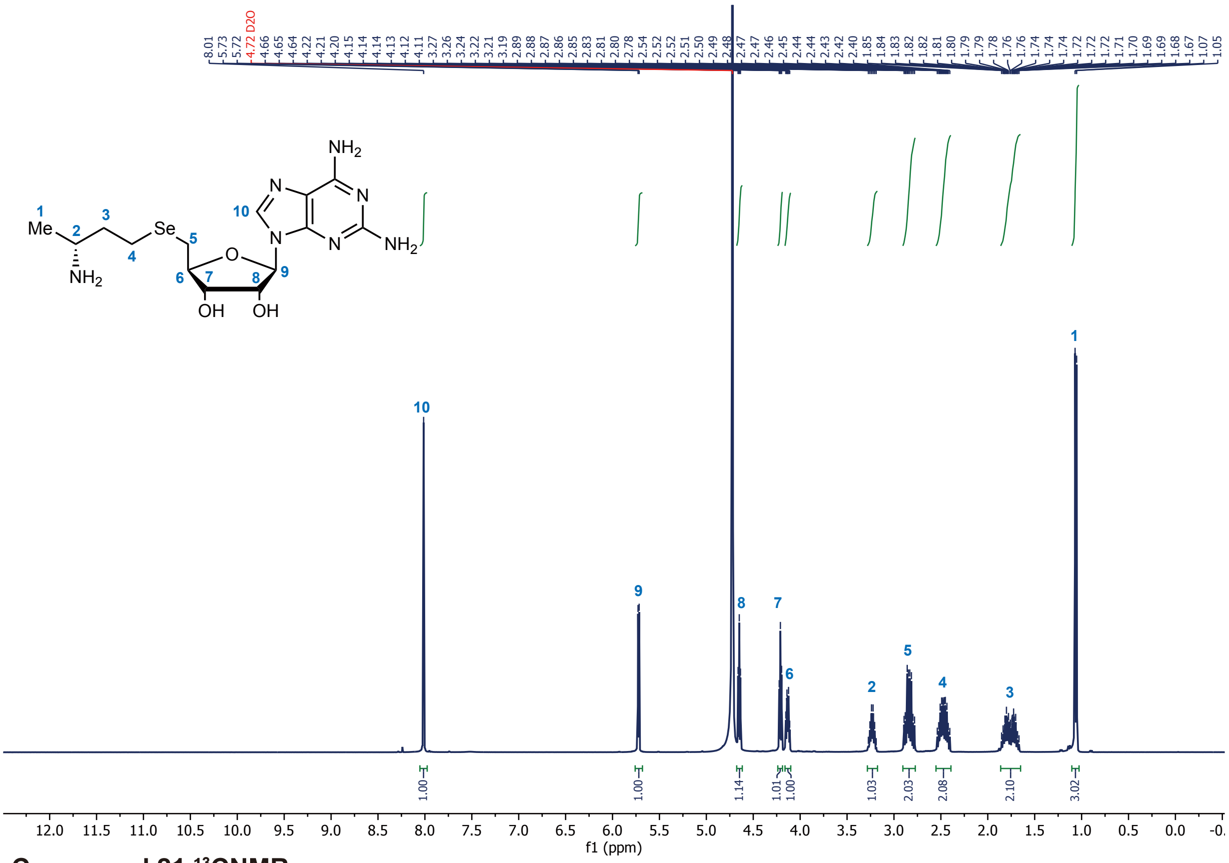

Compound 21 ¹³CNMR

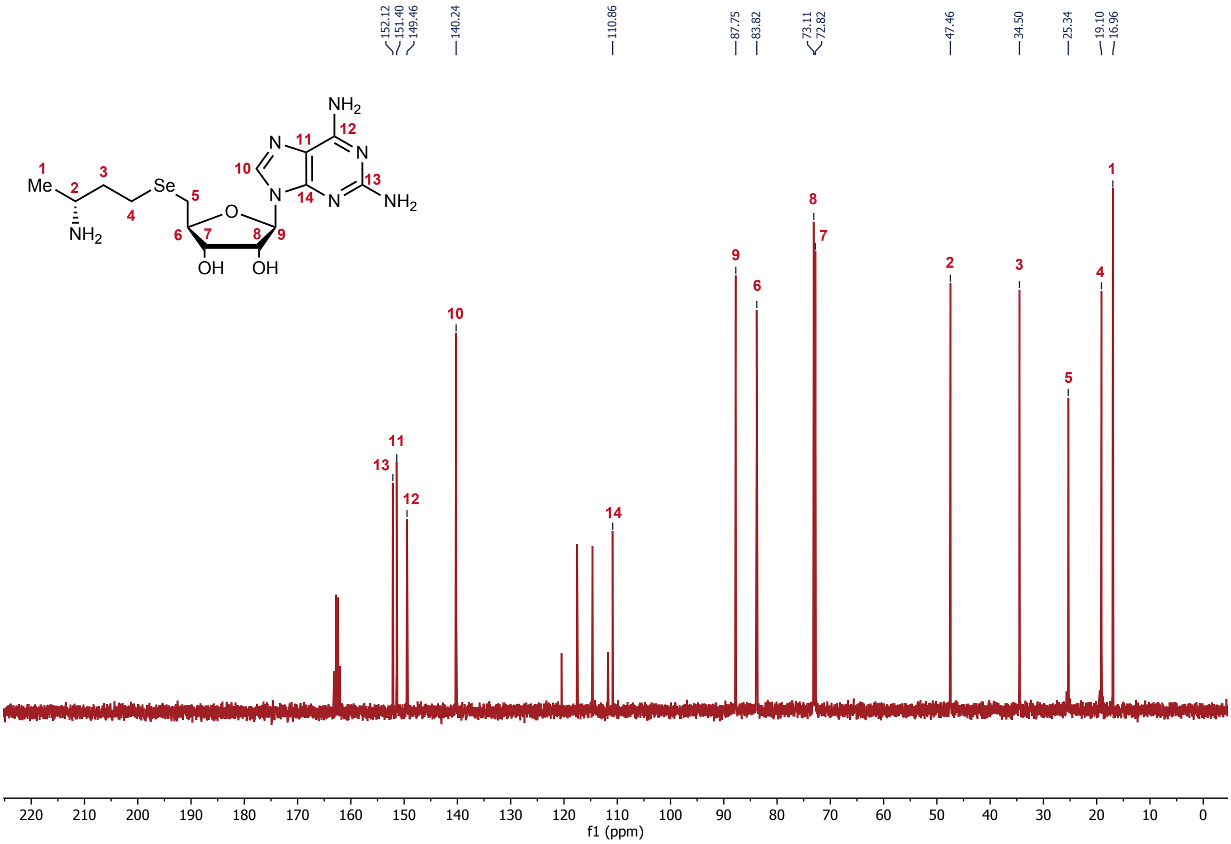

Compound 5 ^{1}HNMR

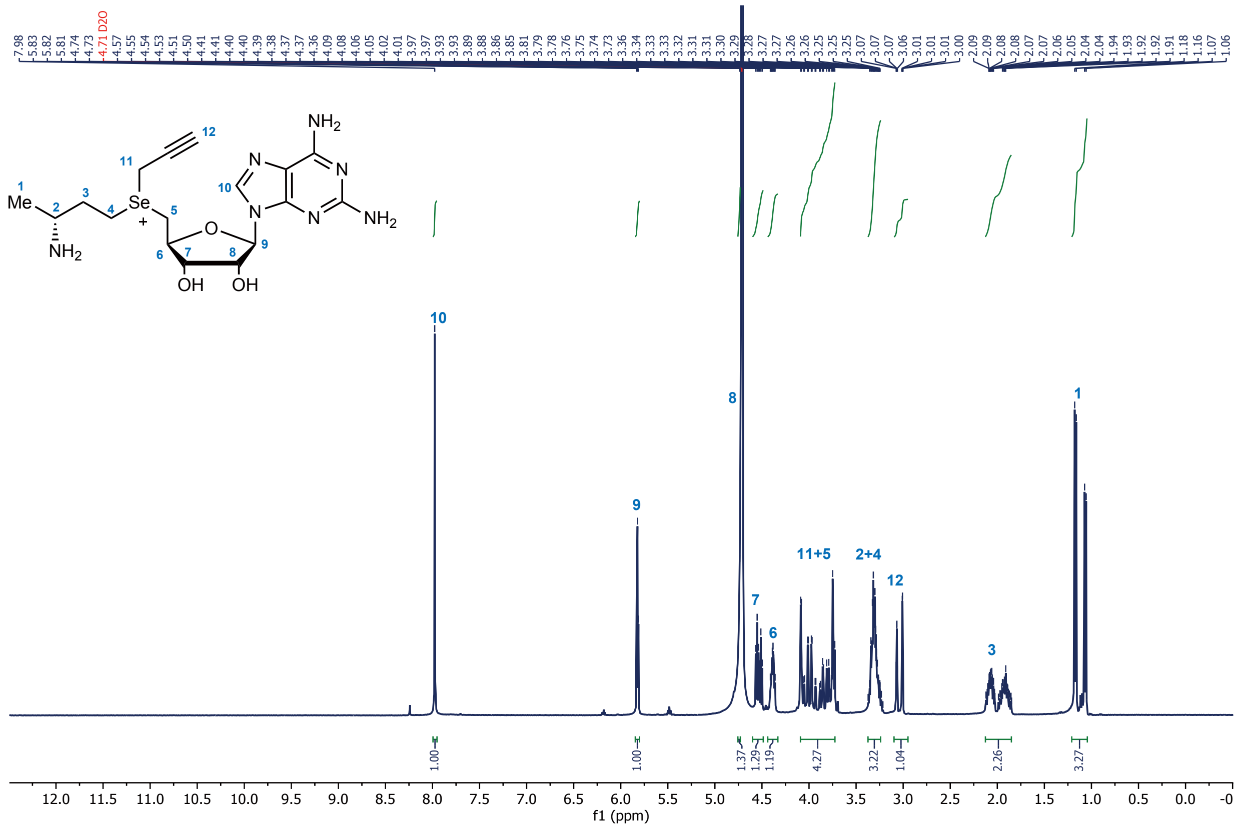

Compound 5 ^{13}CNMR

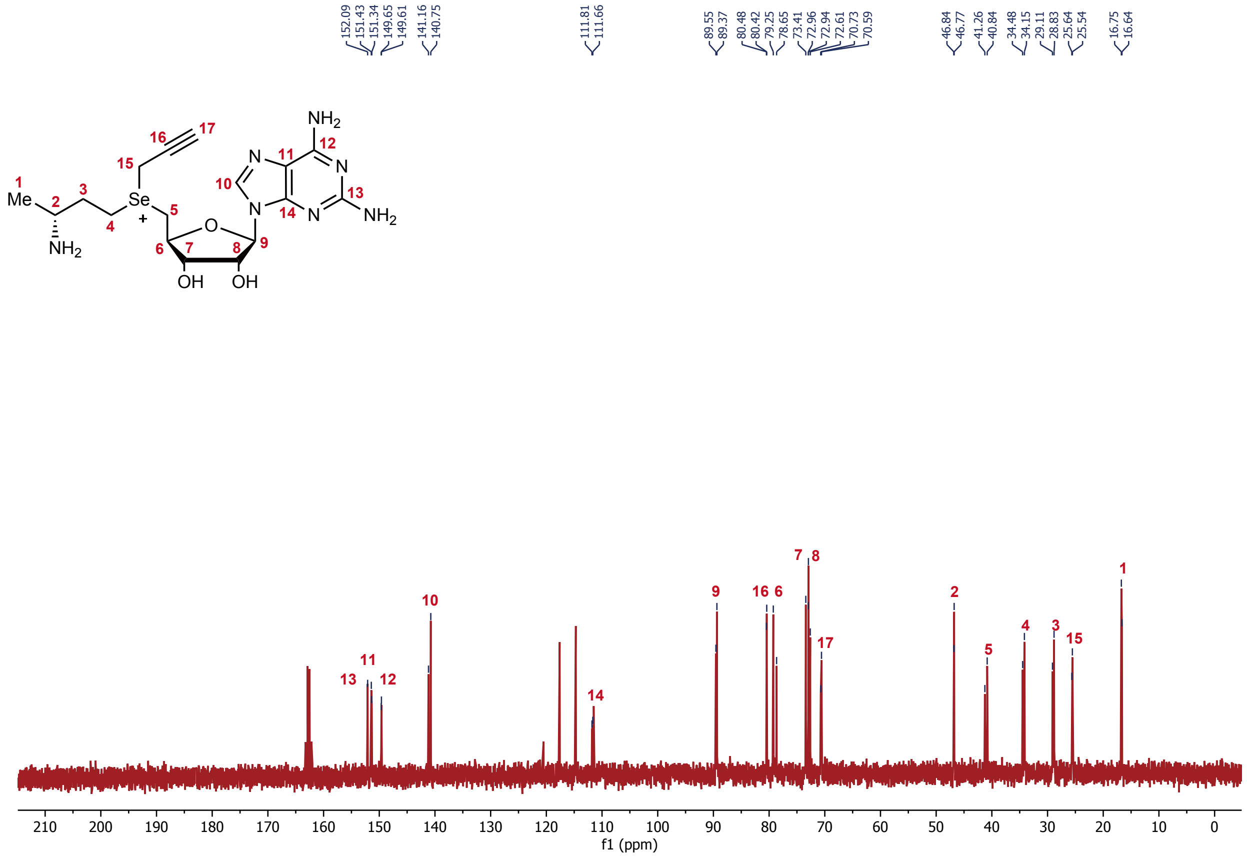

Compound 22 ^{1}HNMR

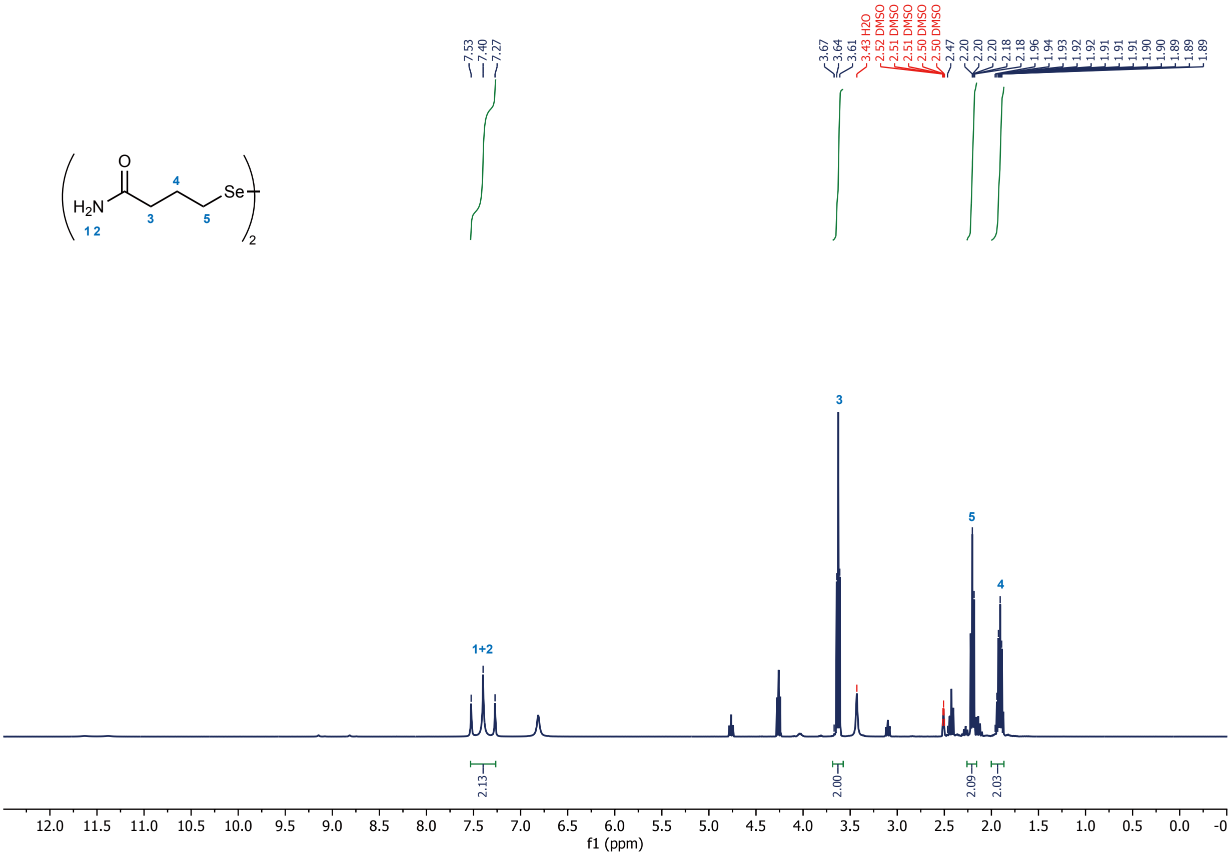

Compound 22 ^{13}CNMR

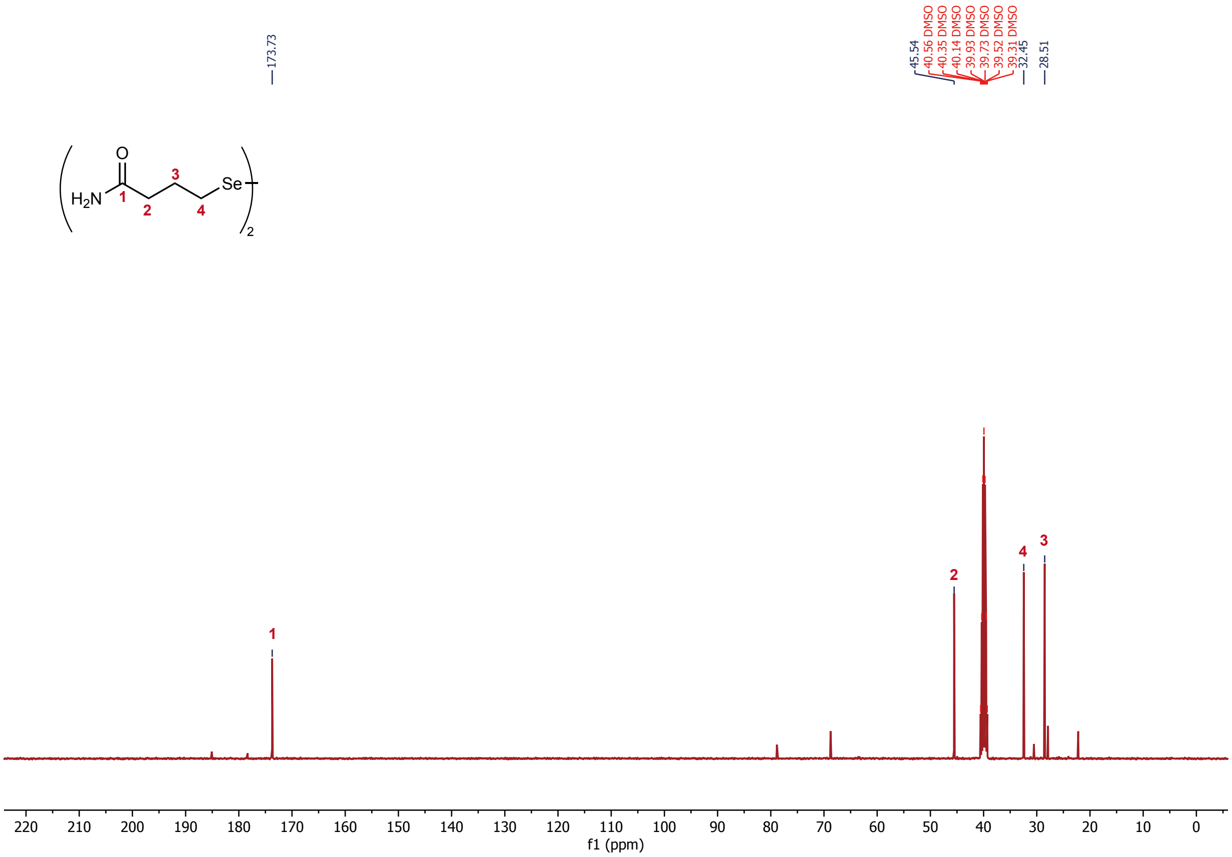

Compound 24 ^{1}HNMR

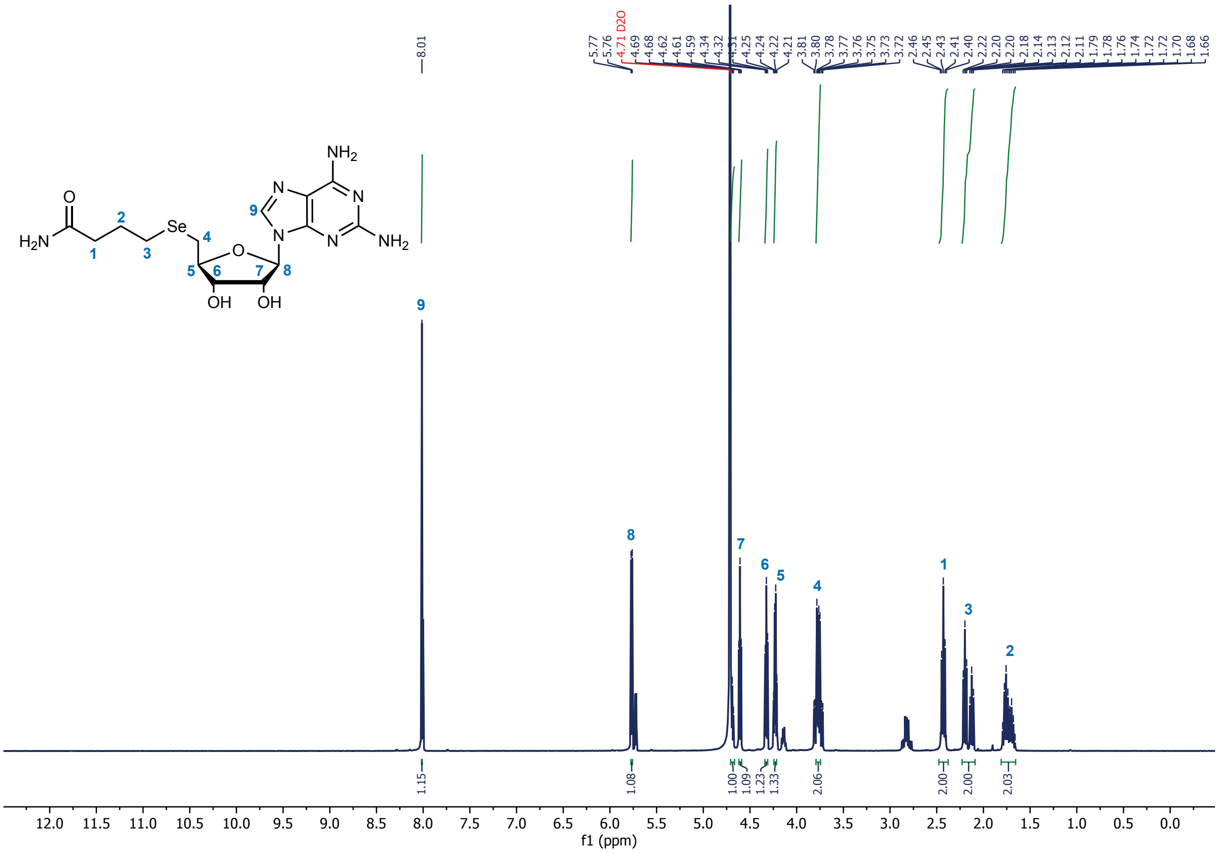

Compound 24 ^{13}CNMR

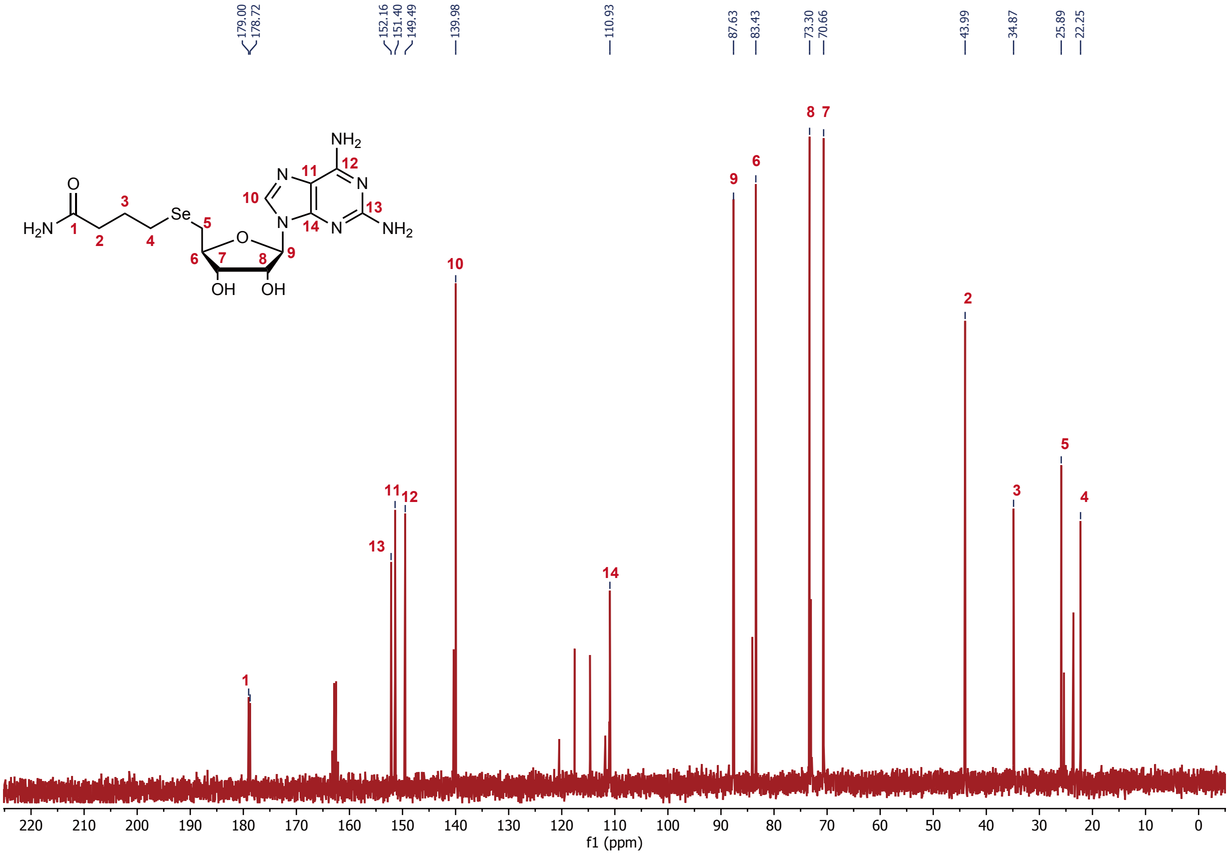

Compound 7 ¹HNMR

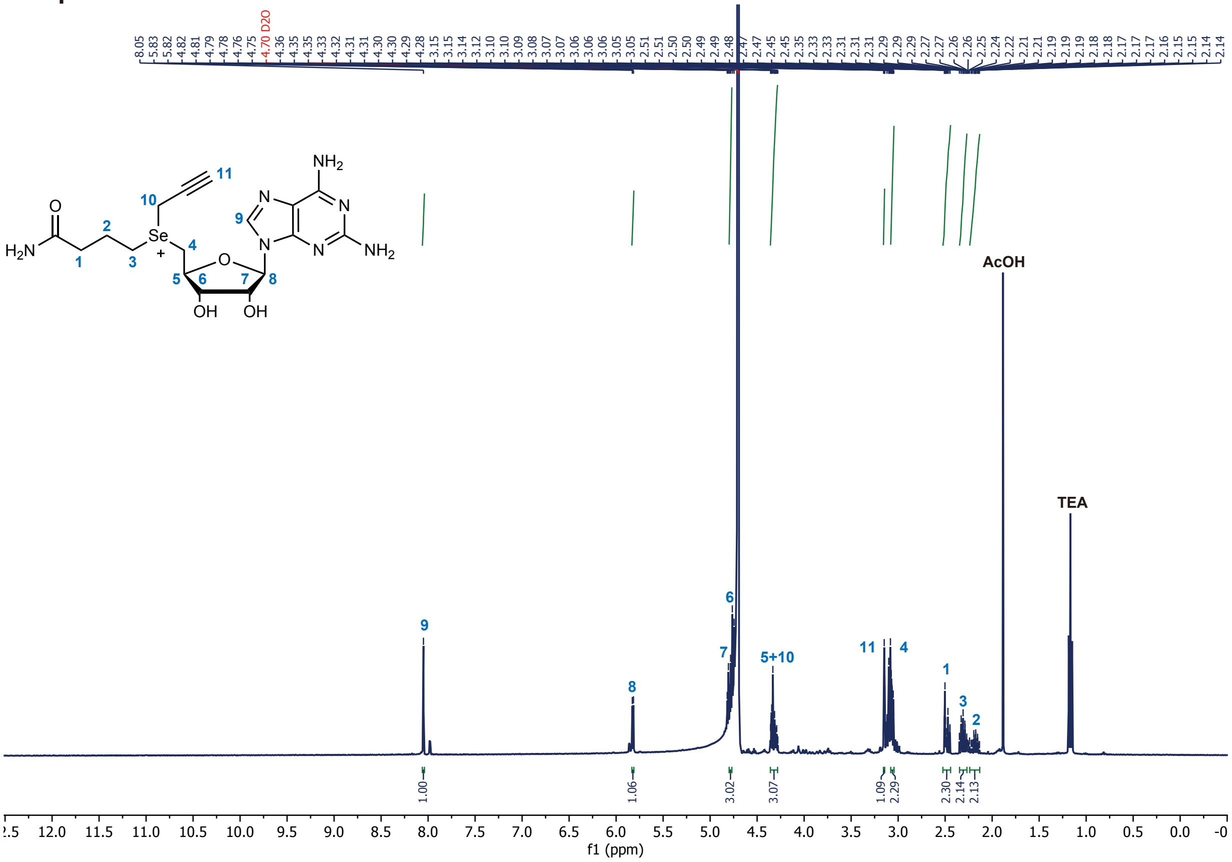

Compound 26 ^{1}HNMR

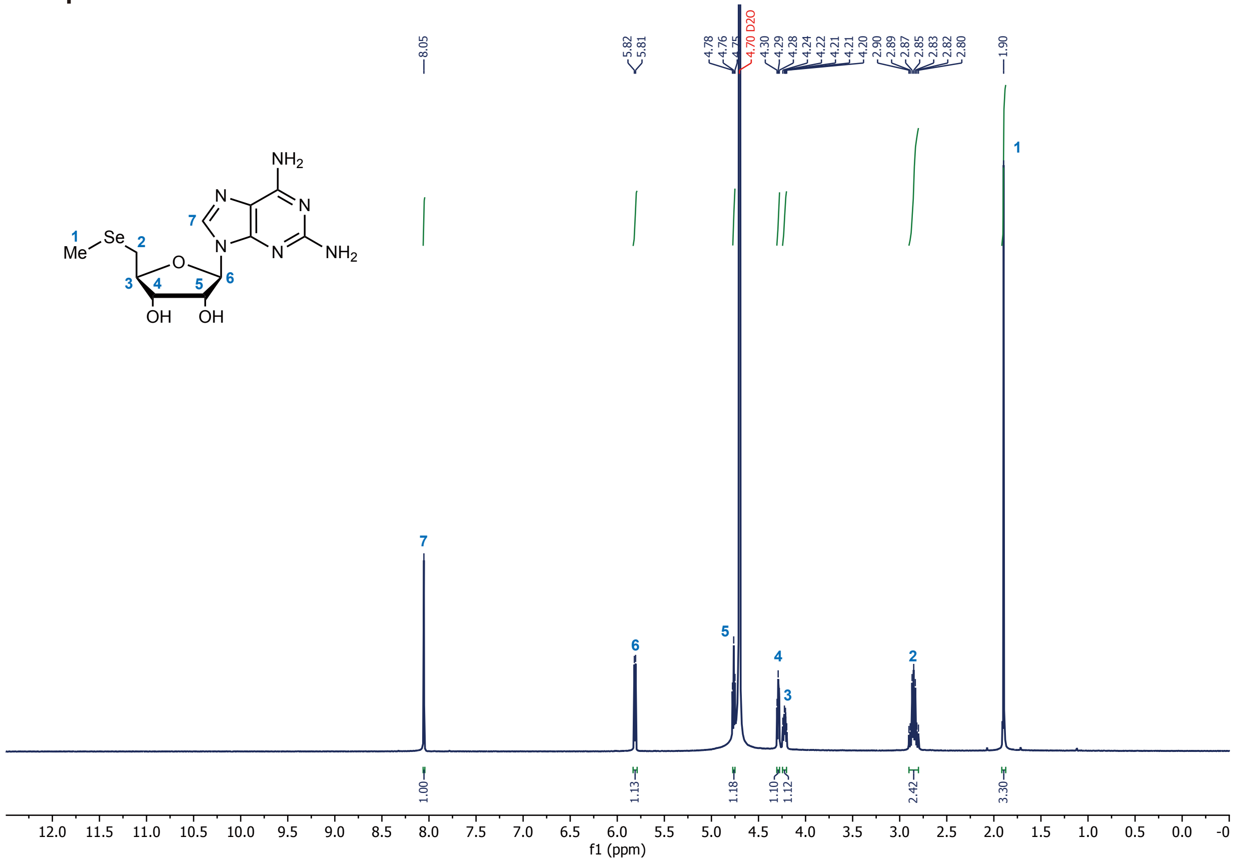

Compound 26 ^{13}CNMR

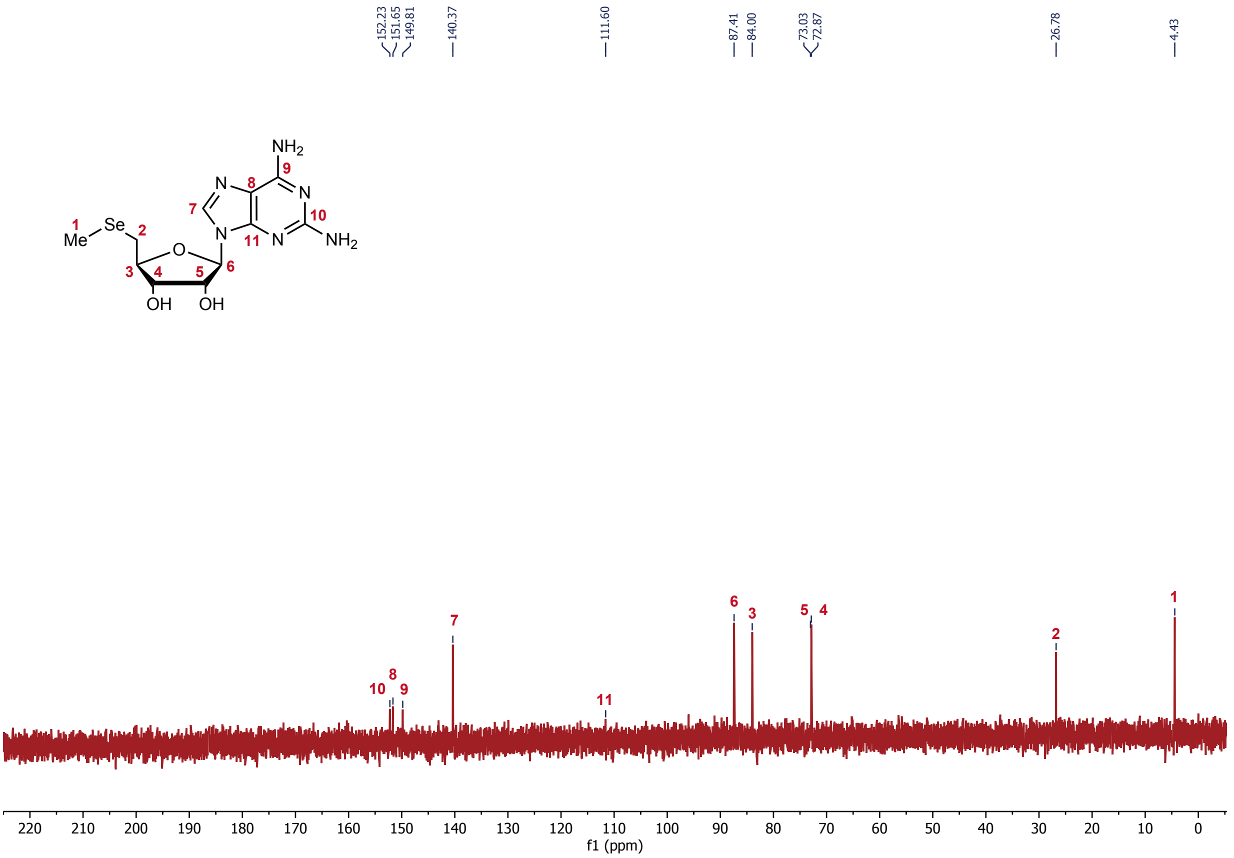

Compound 8 ¹HNMR

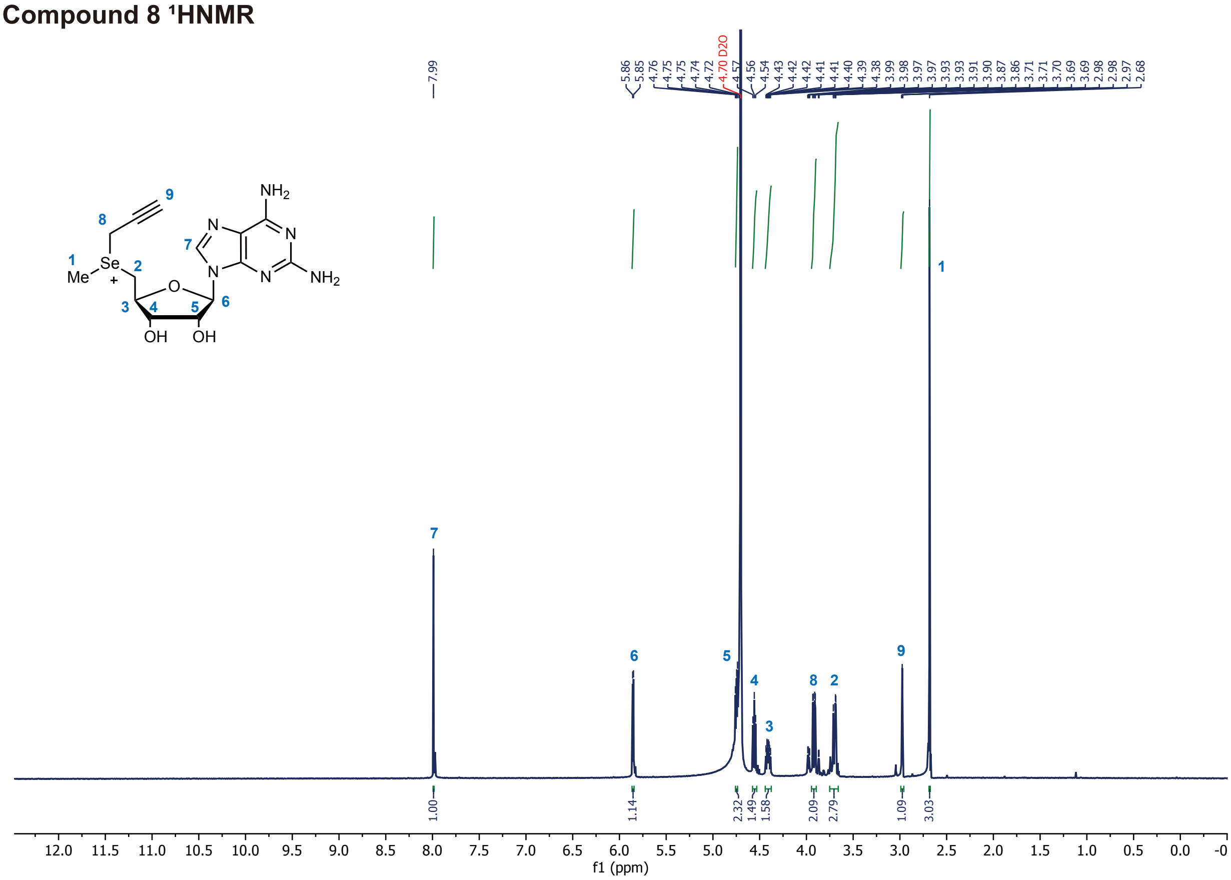

Compound 8 ¹³CNMR

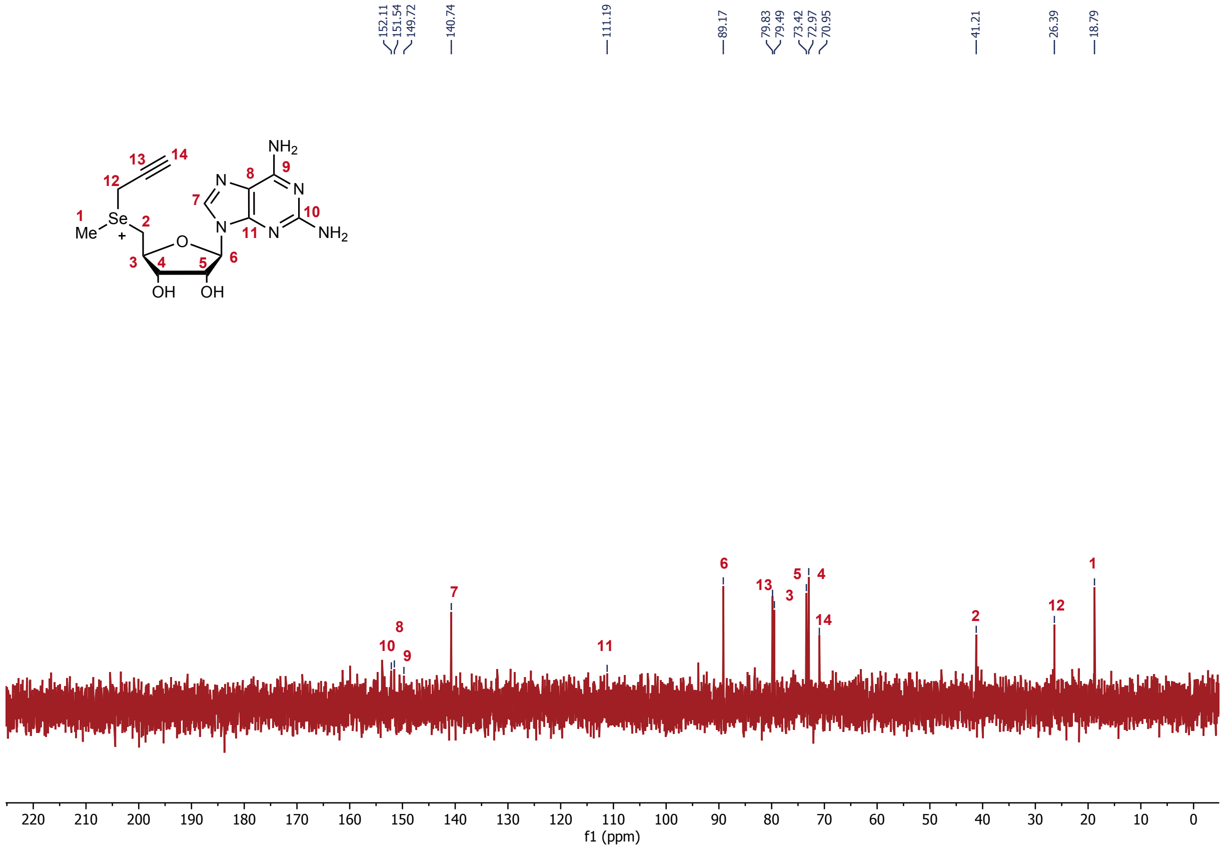

Compound 28 ^{1}HNMR

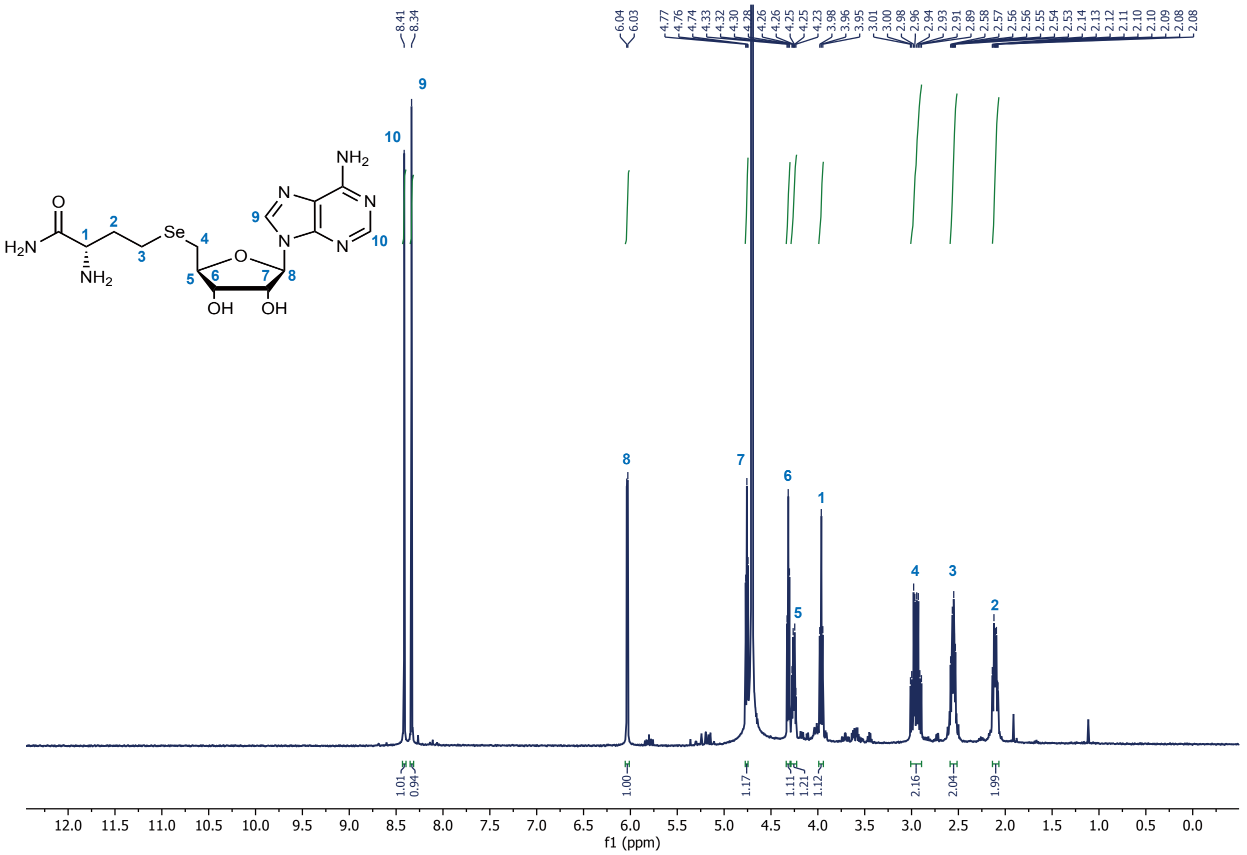

Compound 28 ^{13}CNMR

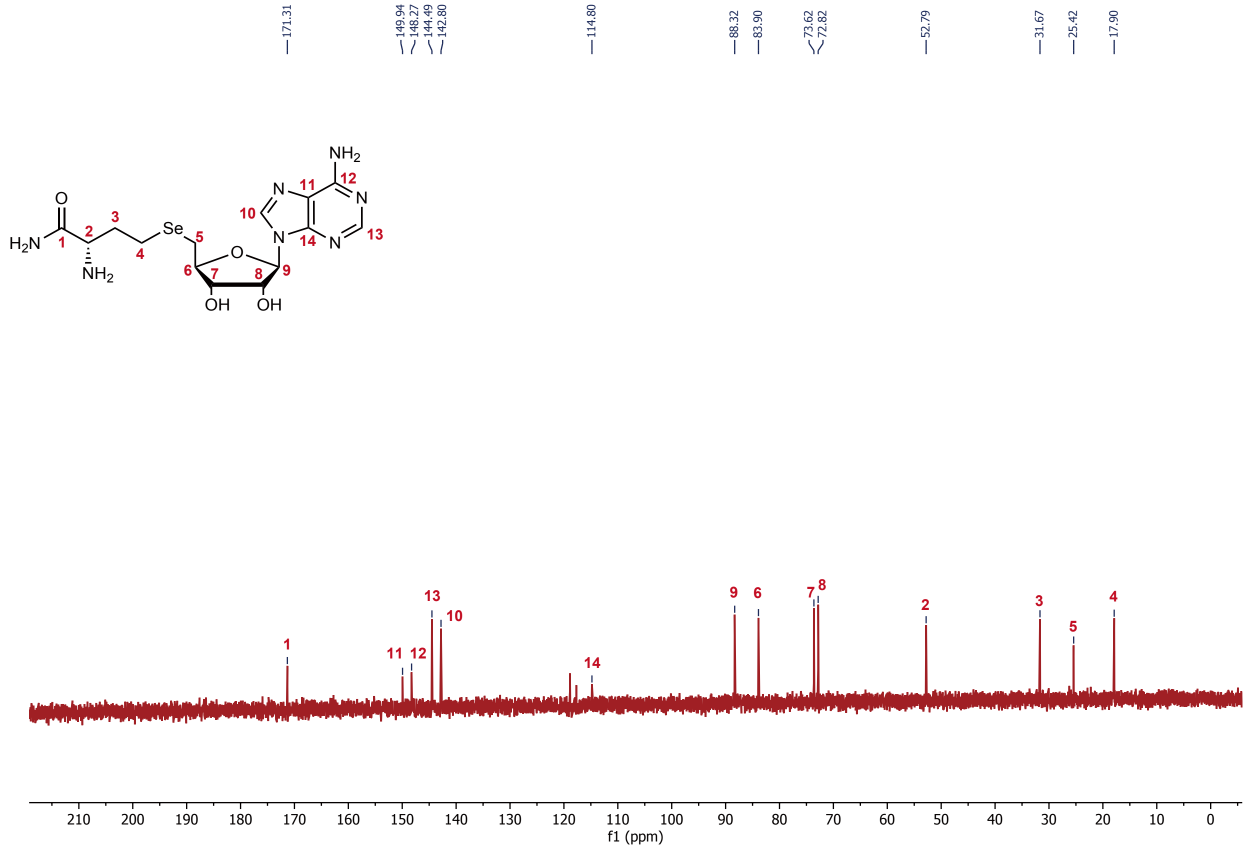

Compound 9 ¹HNMR

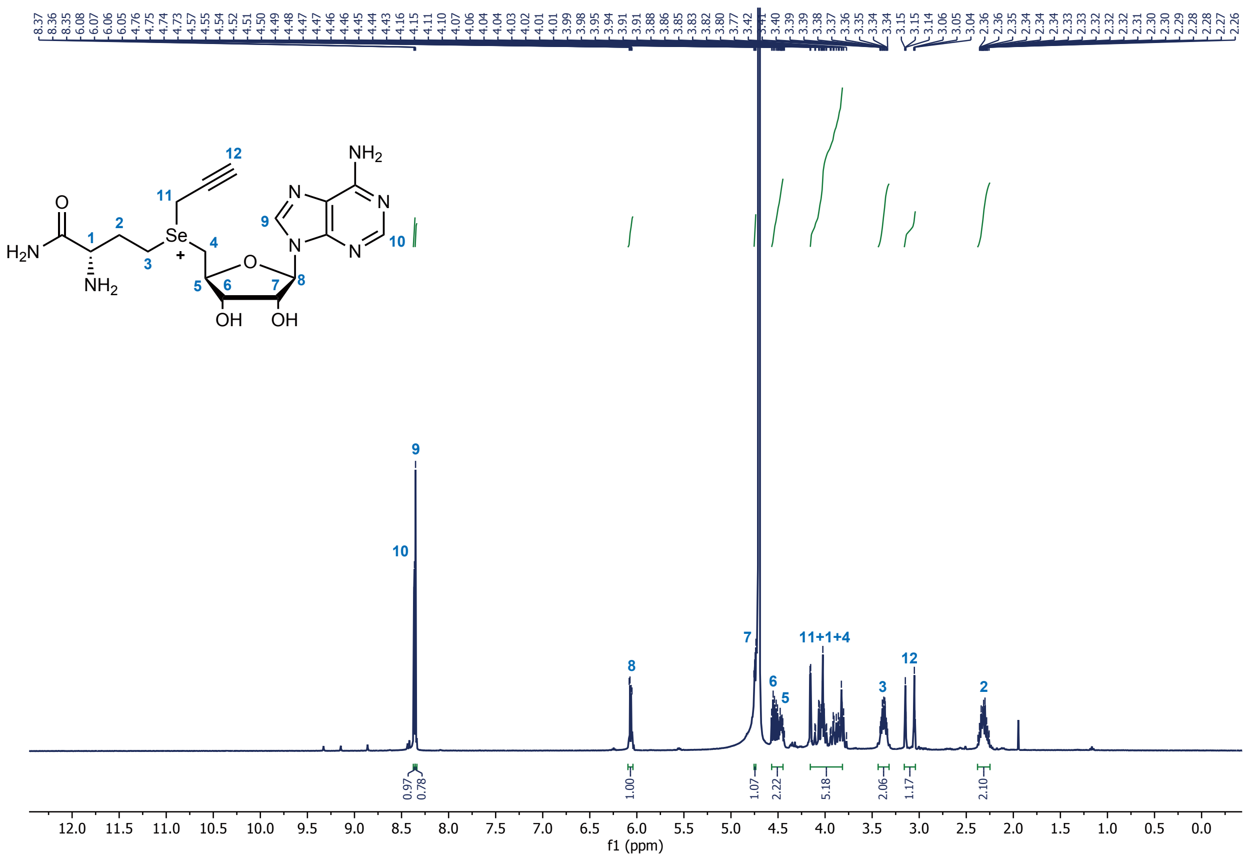

Compound 9 ¹³CNMR

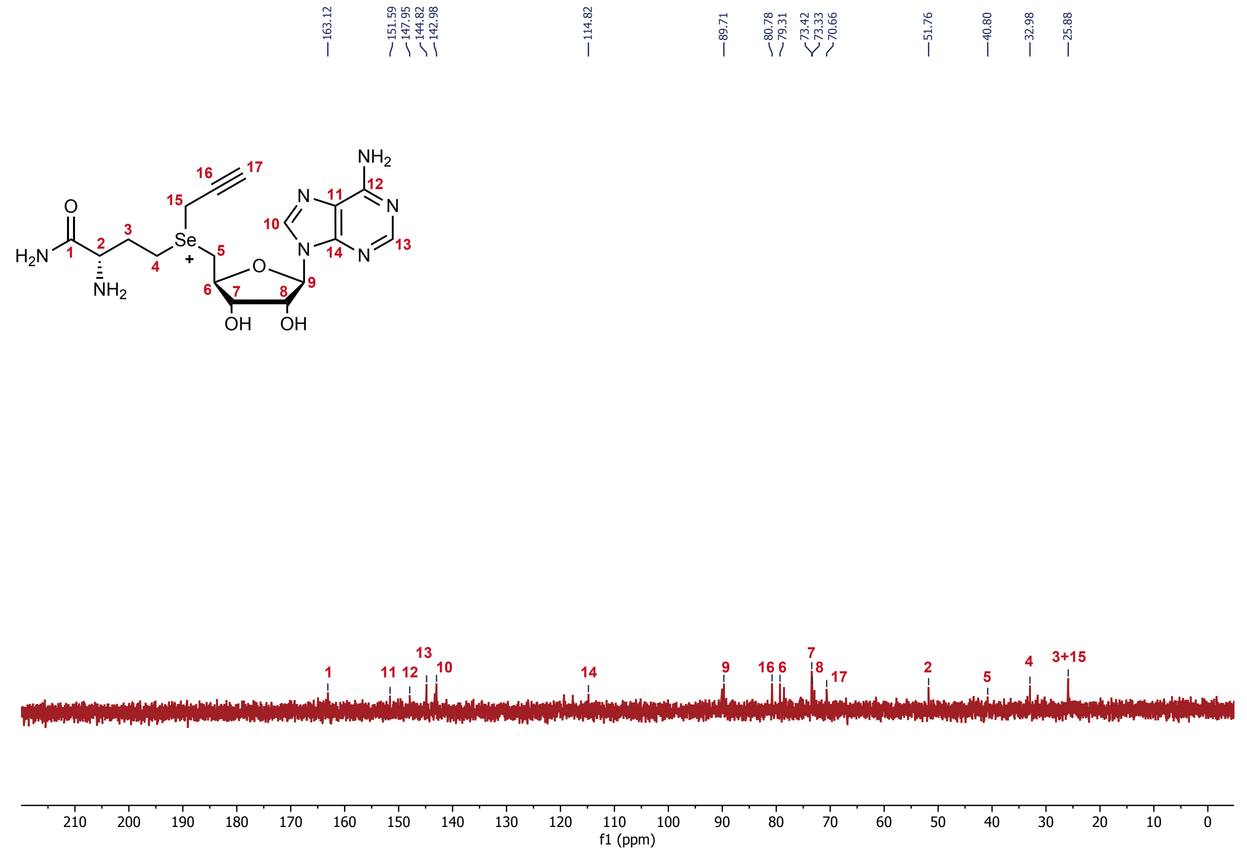

Compound 30 ^{1}HNMR

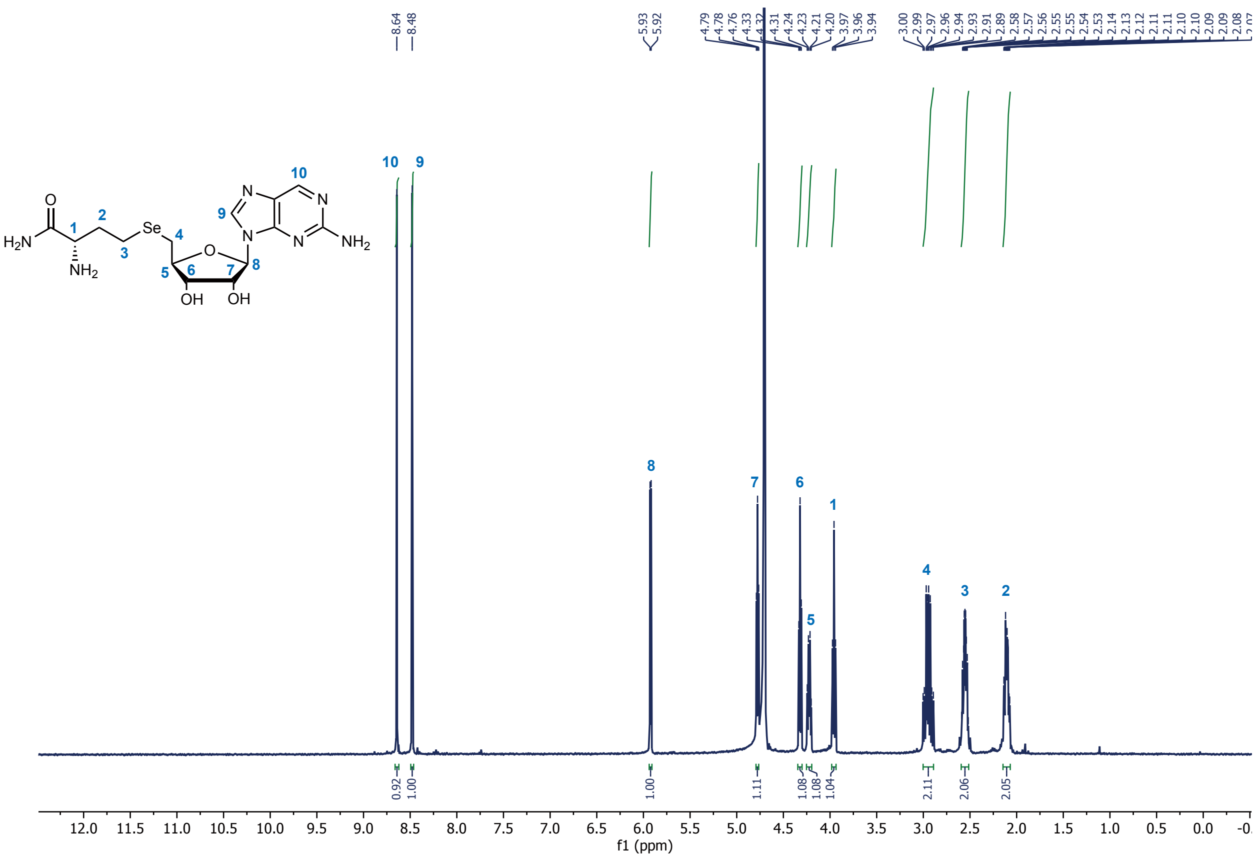

Compound 30 ^{13}CNMR

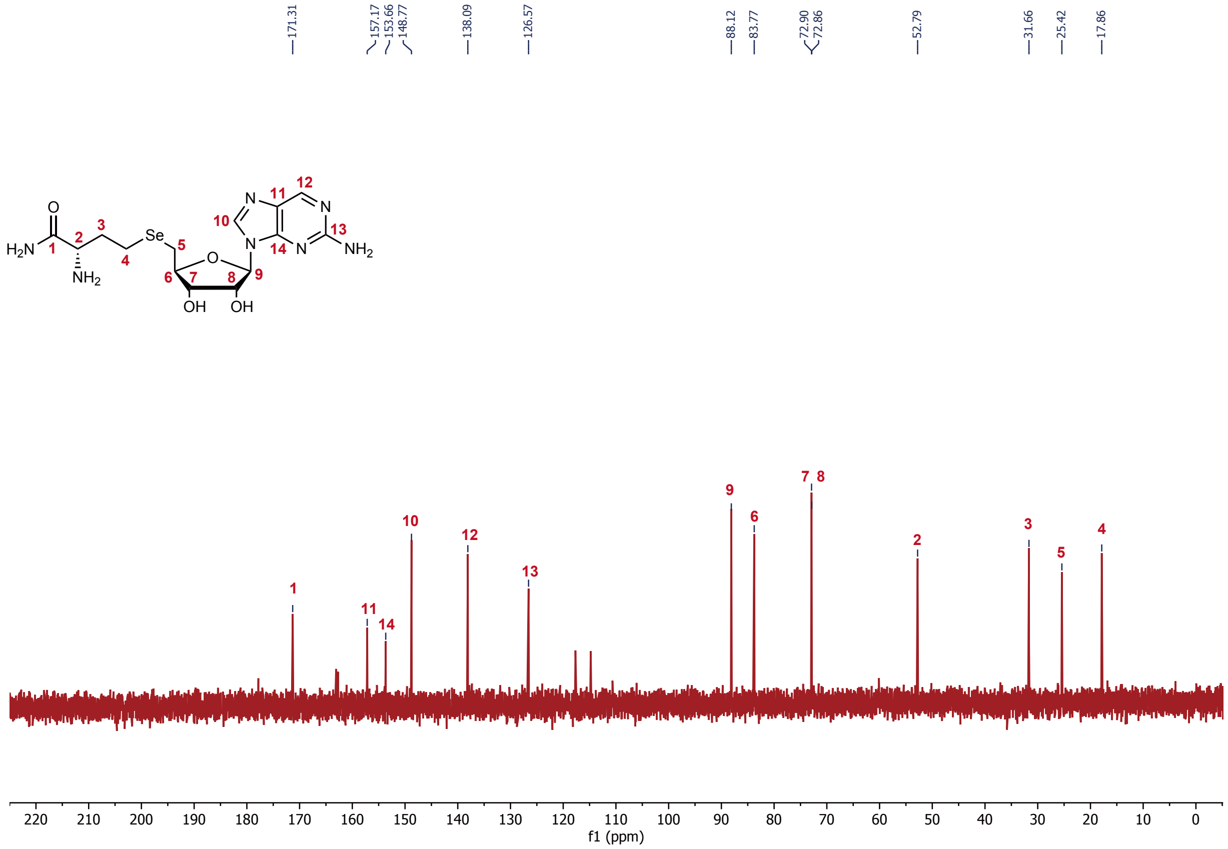

Compound 10 ^{1}HNMR

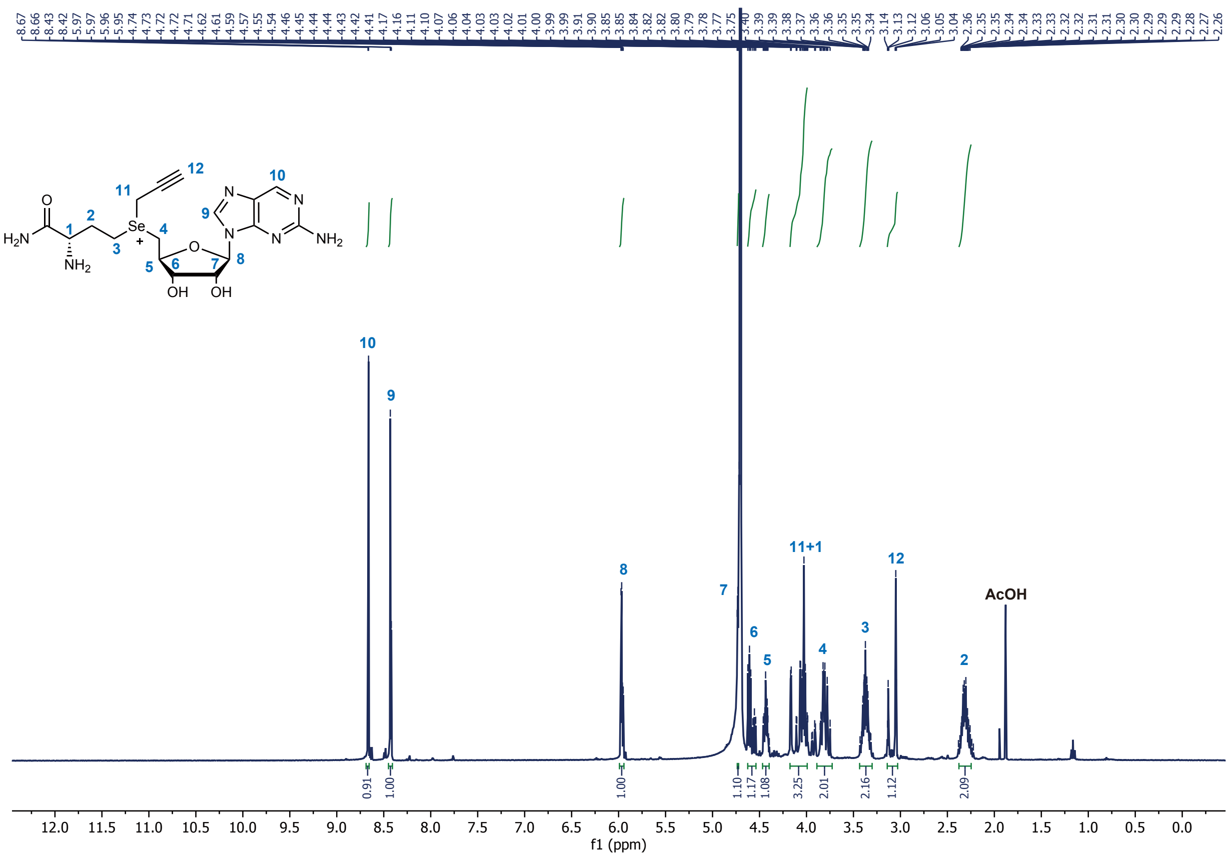

Compound 10 ^{13}CNMR

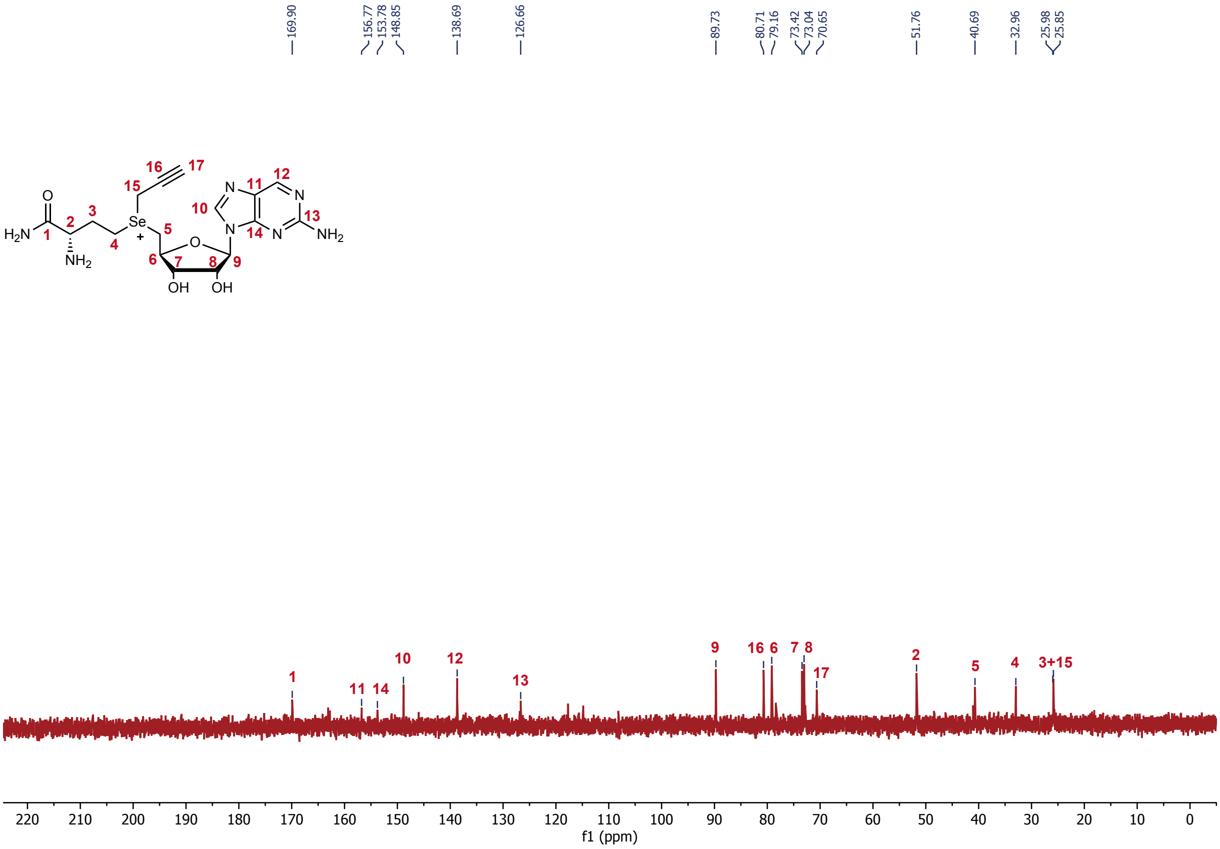

Compound 32 ^{1}HNMR

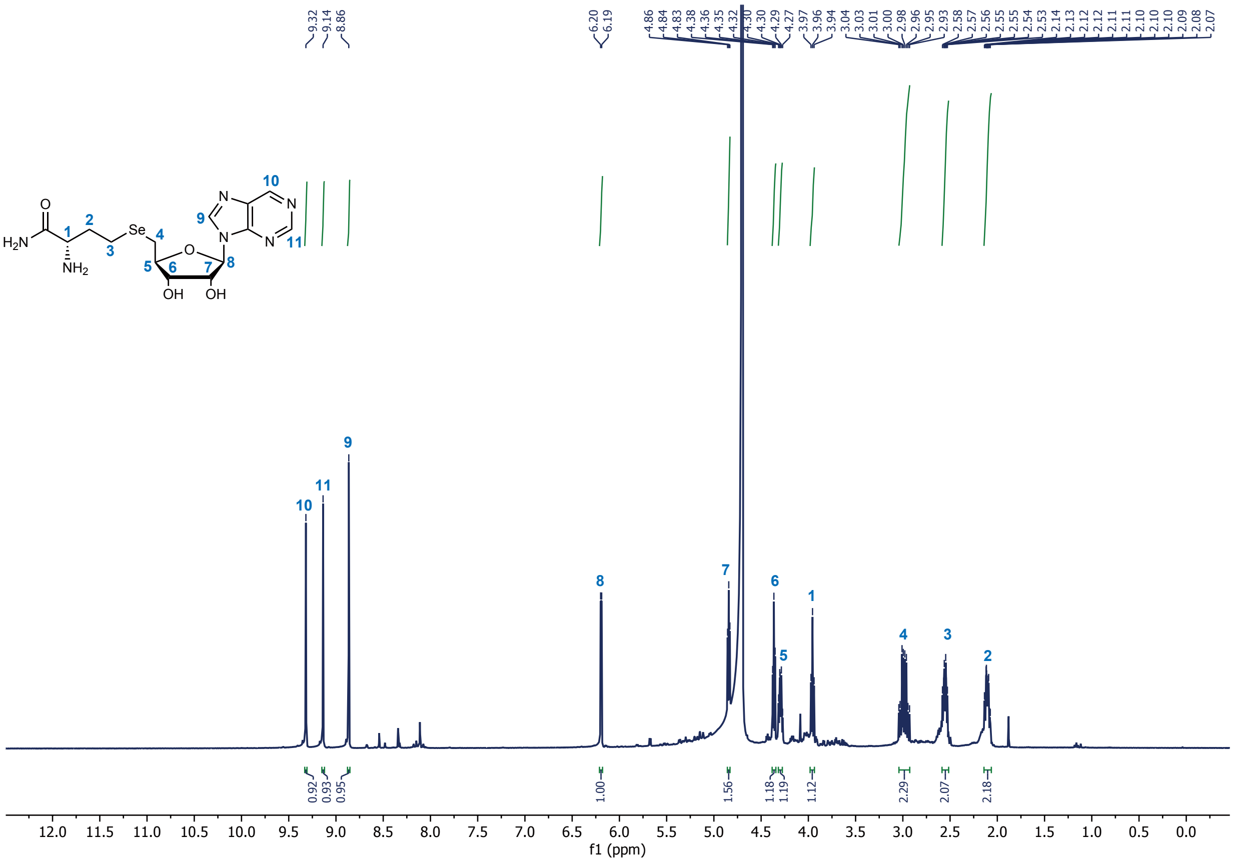

Compound 32 ^{13}CNMR

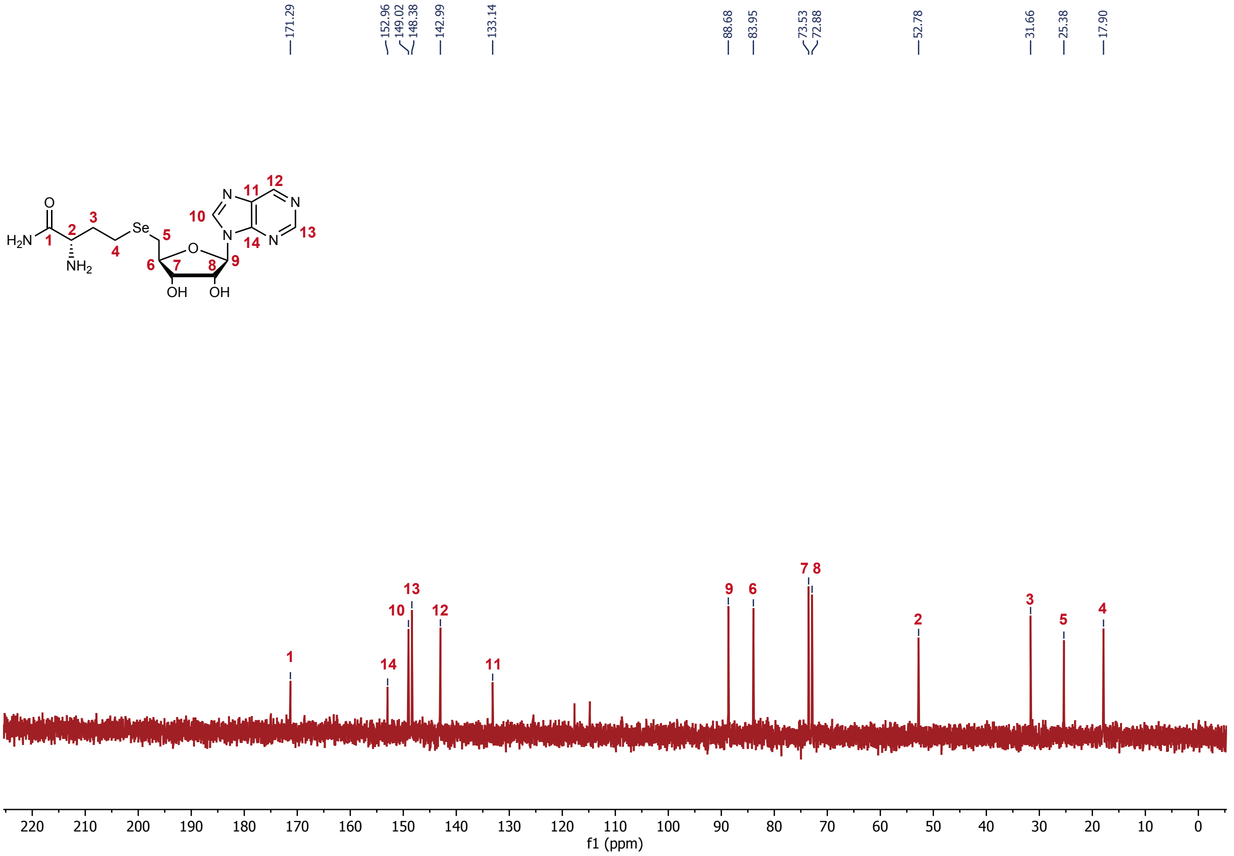

Compound 11 ^{1}HNMR

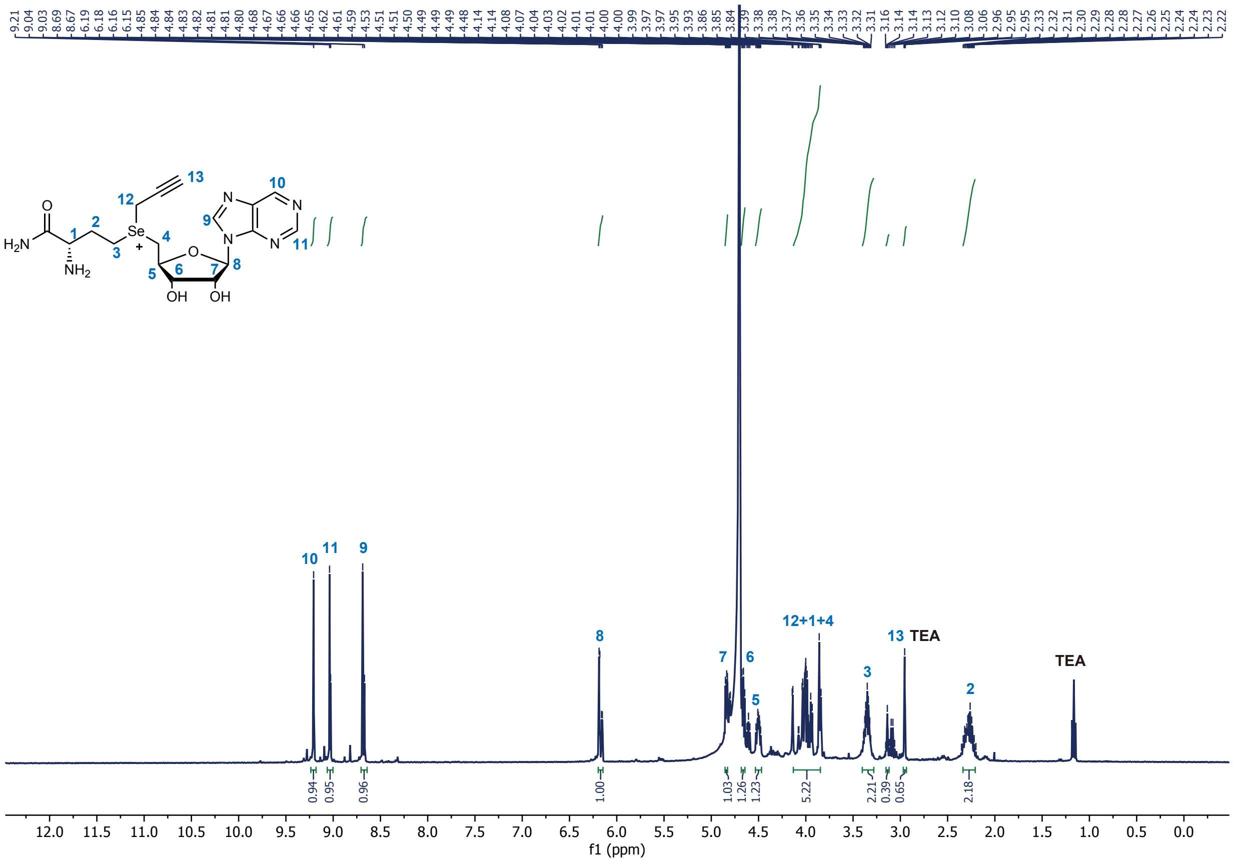

Compound 11 ^{13}CNMR

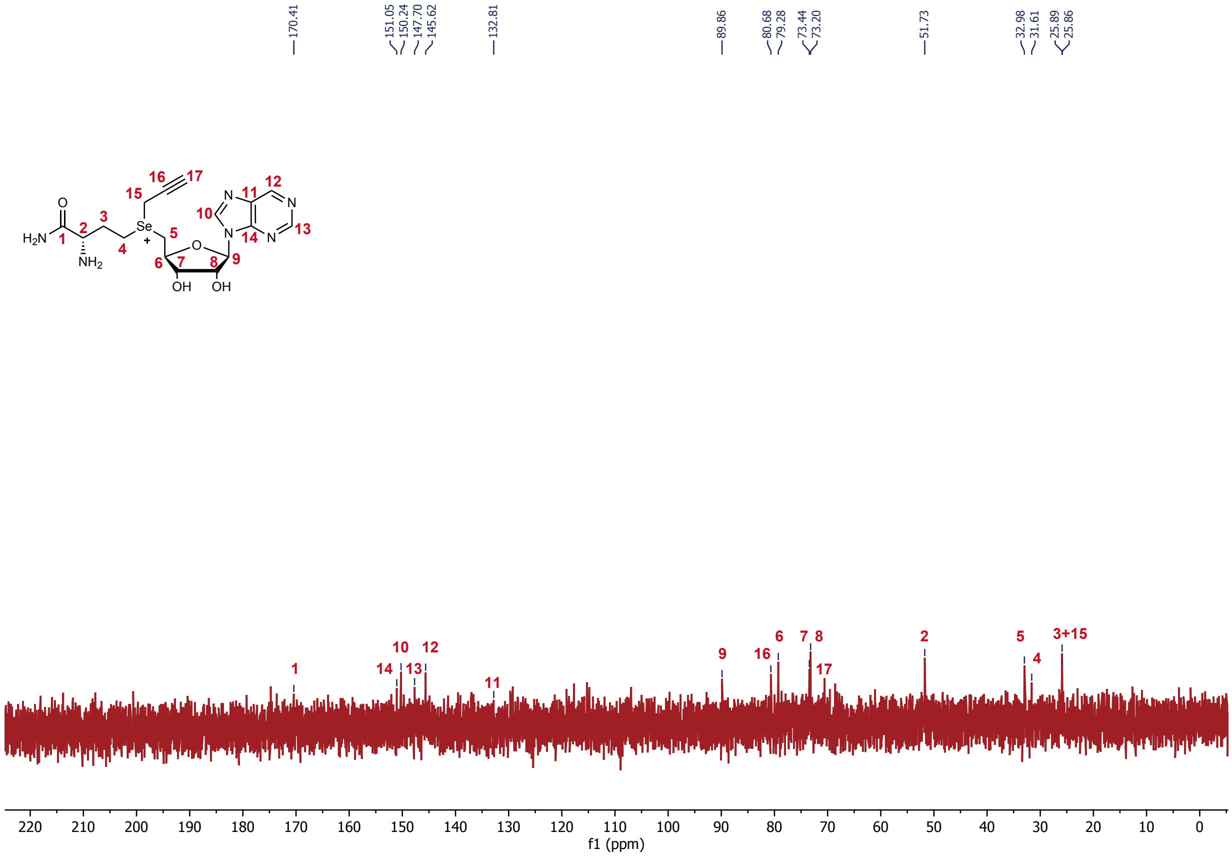

Compound 34 ¹HNMR

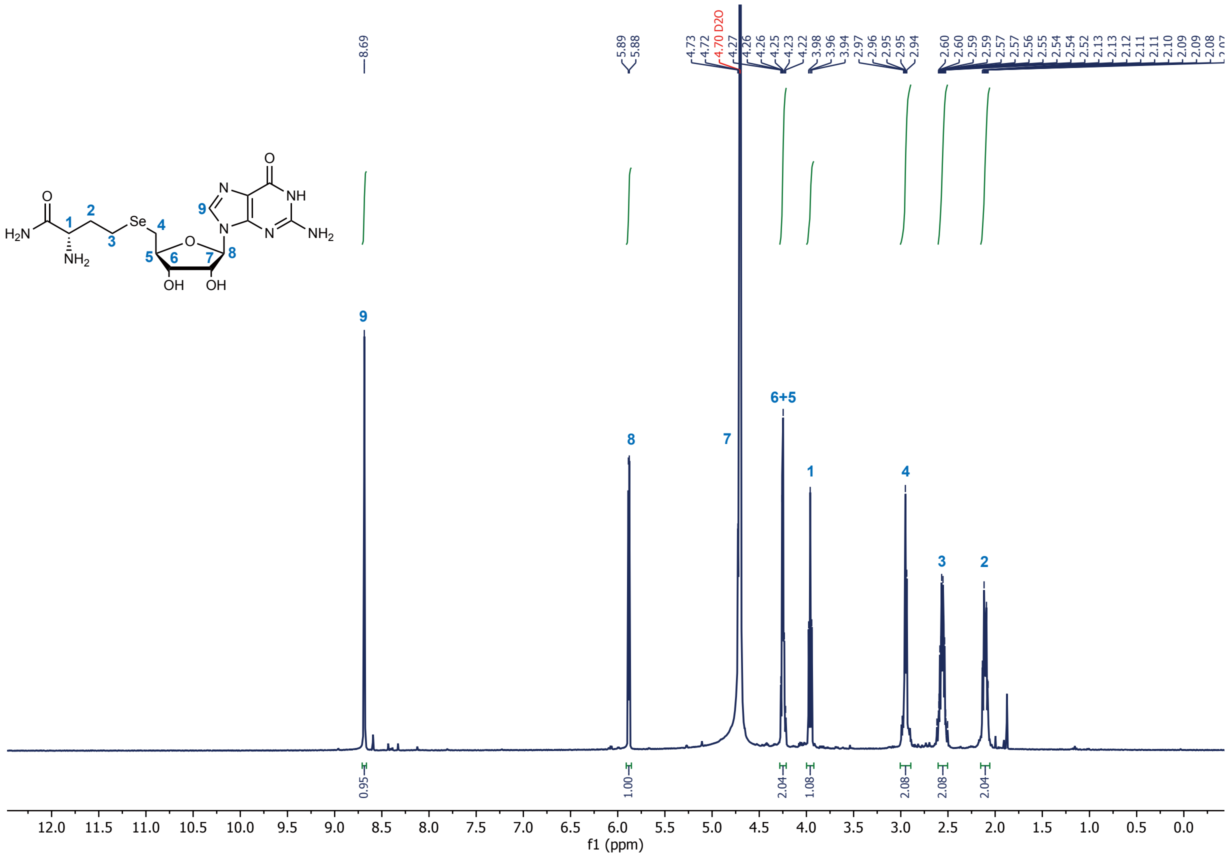

Compound 34 ¹³CNMR

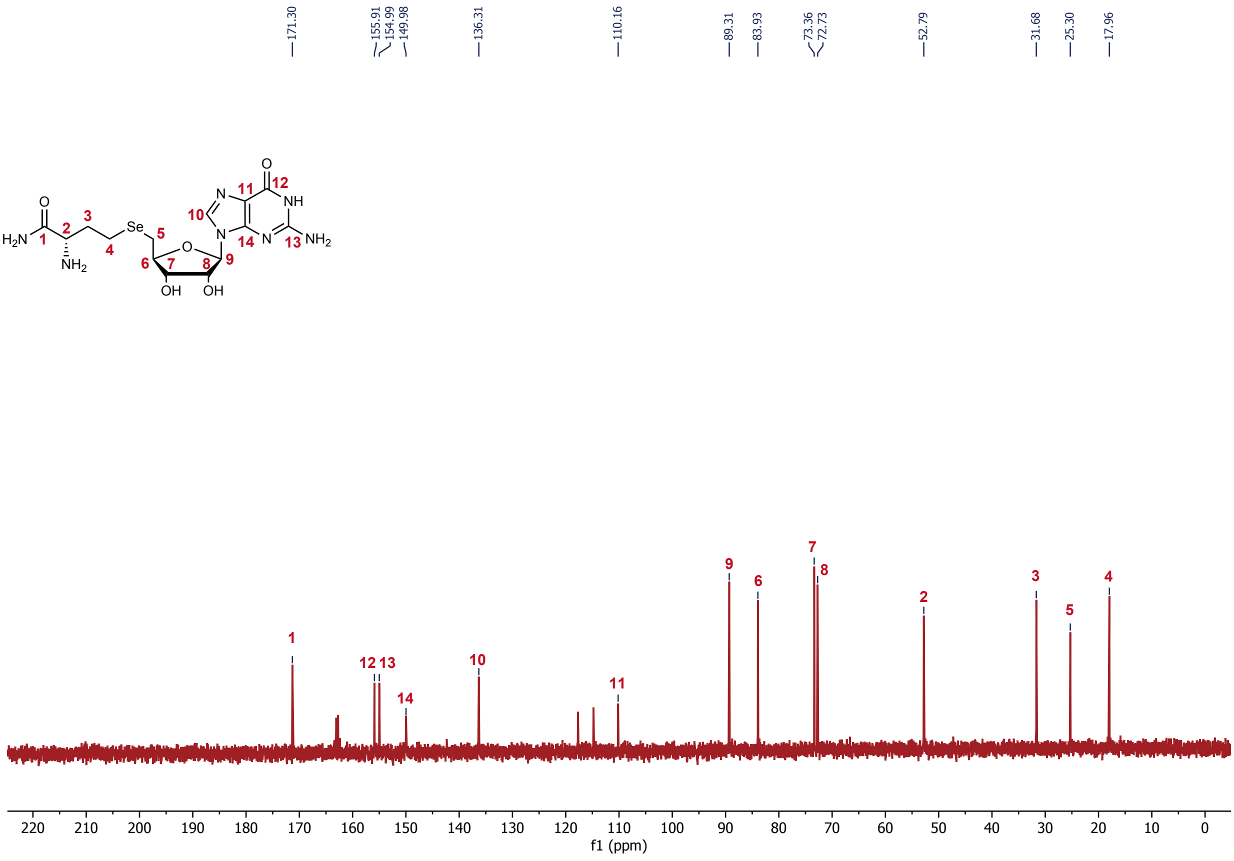

Compound 12 ¹HNMR

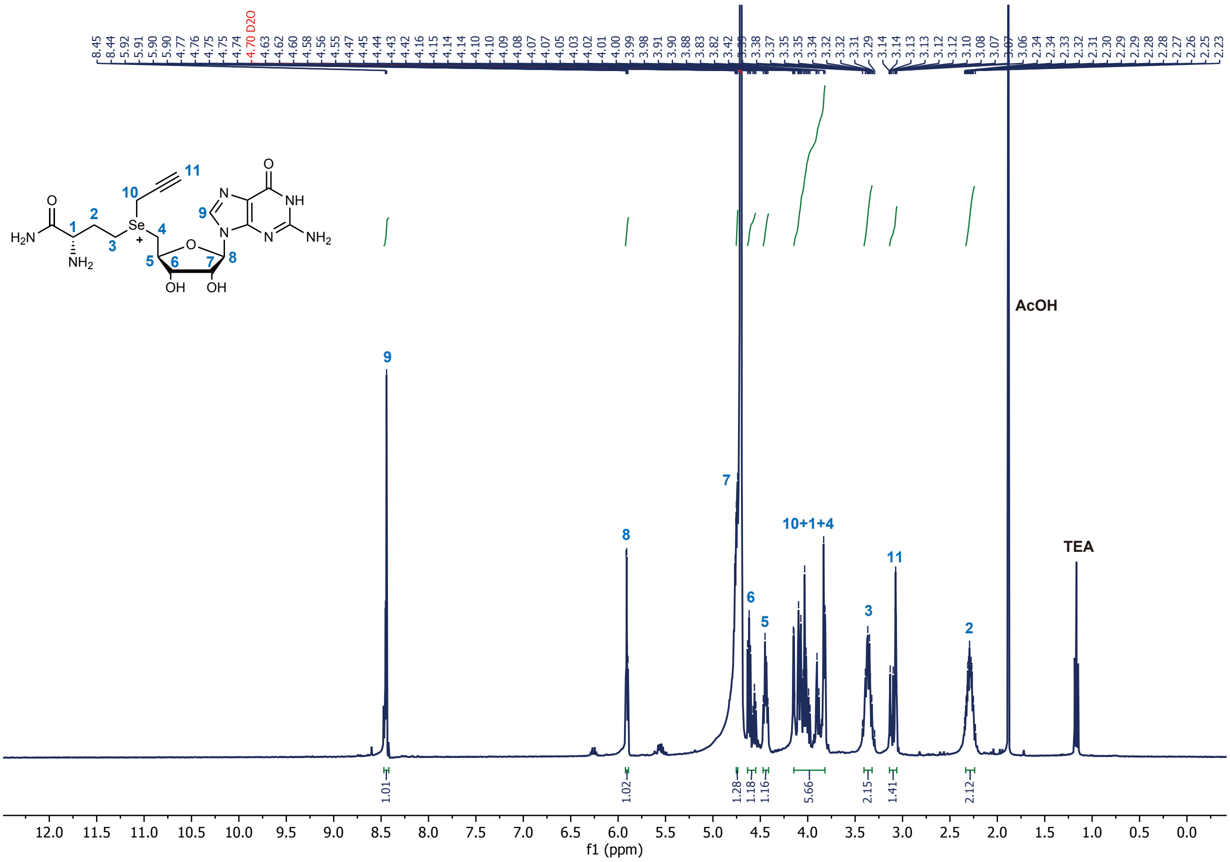

Compound 12 ¹³CNMR

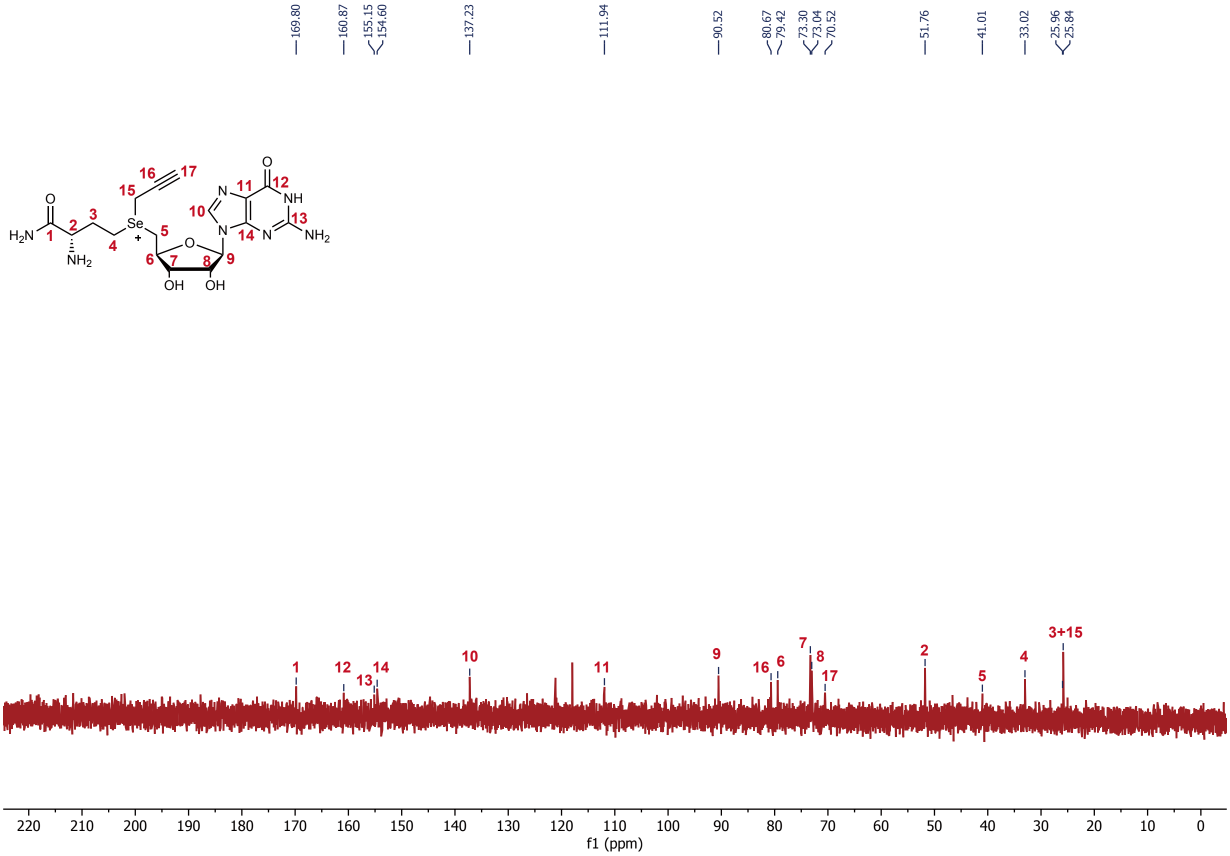

Compound 36 ¹HNMR

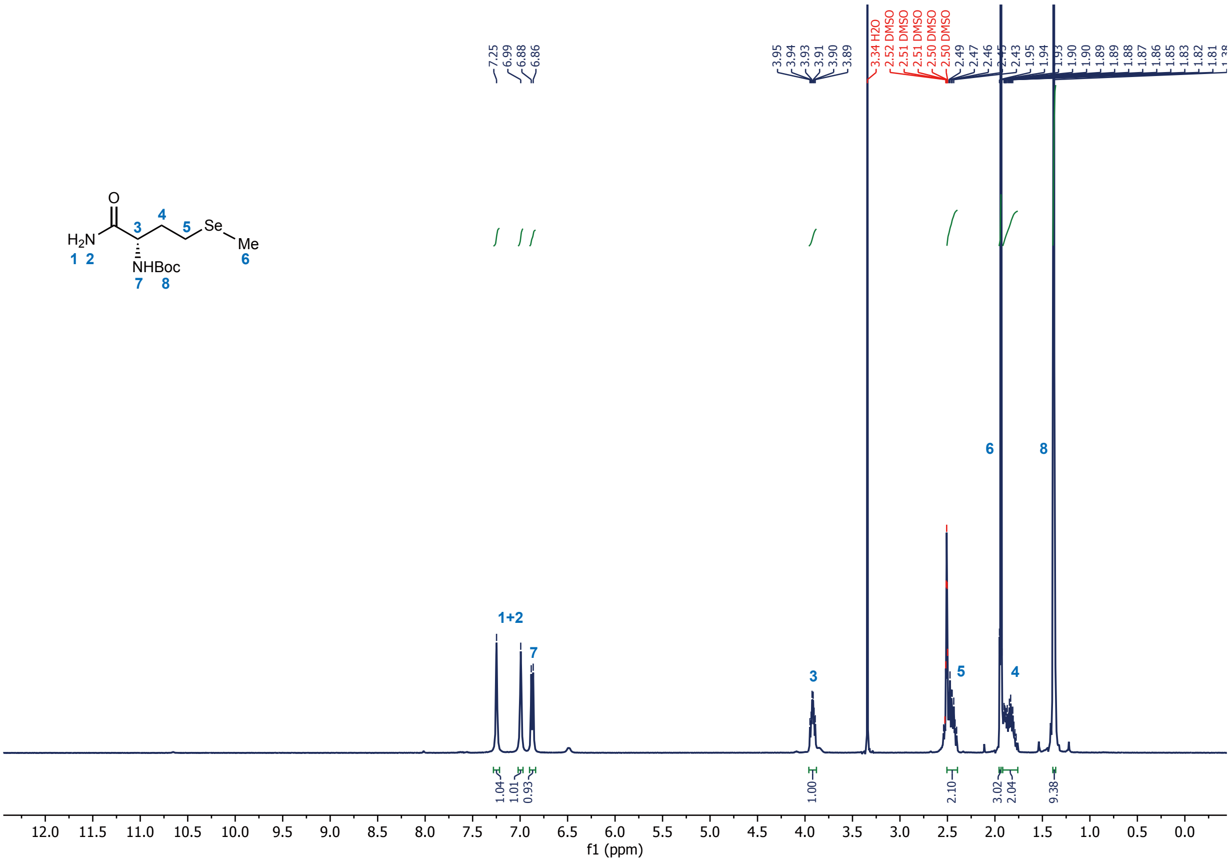

Compound 36 ¹³CNMR

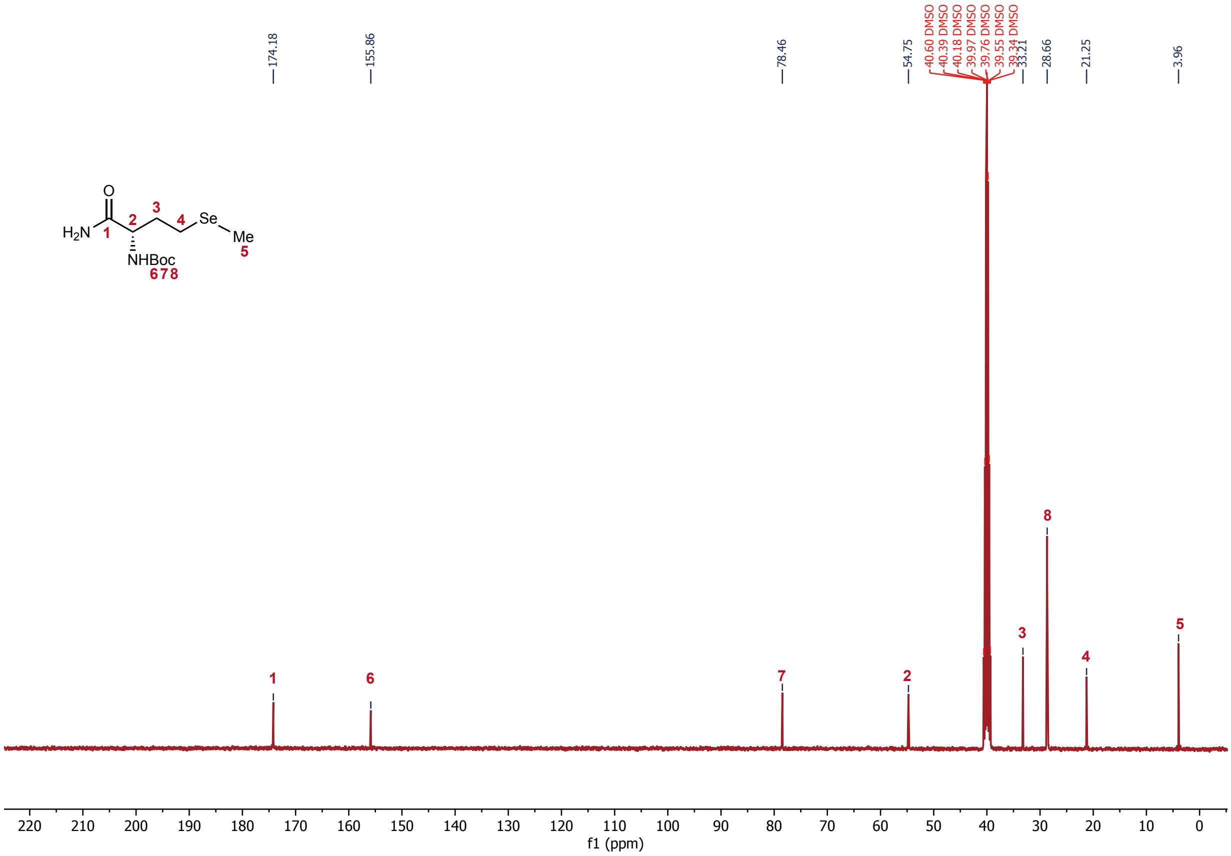

Compound 13 ¹HNMR

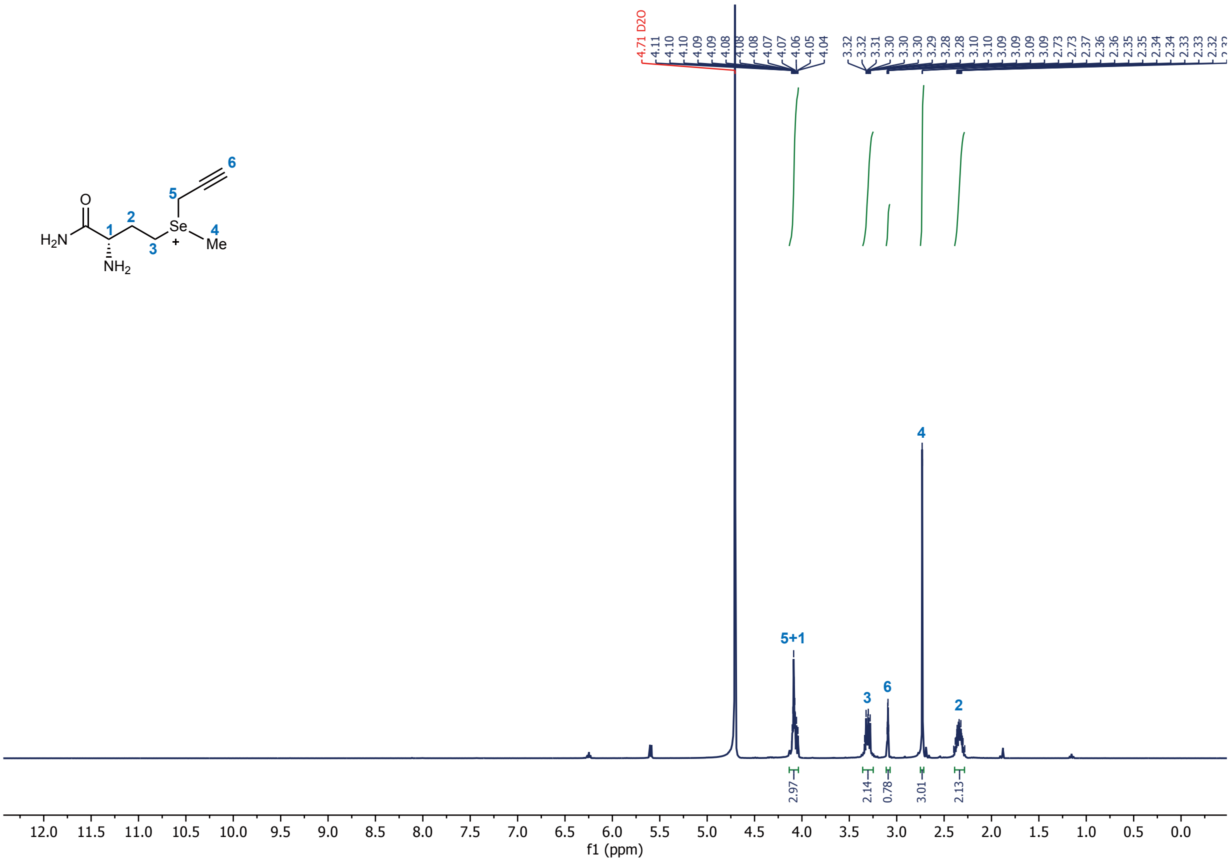

Compound 13 ¹³CNMR

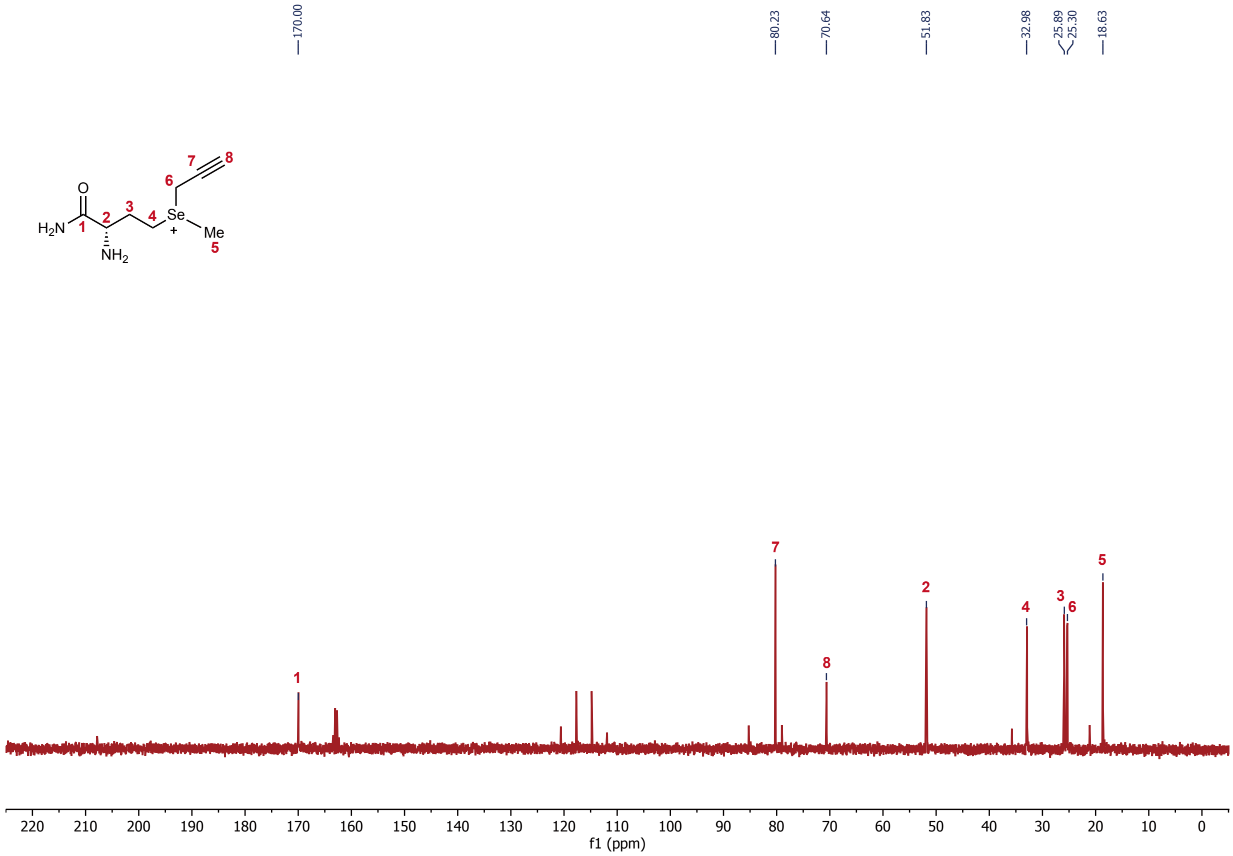

Compound 37 ¹HNMR

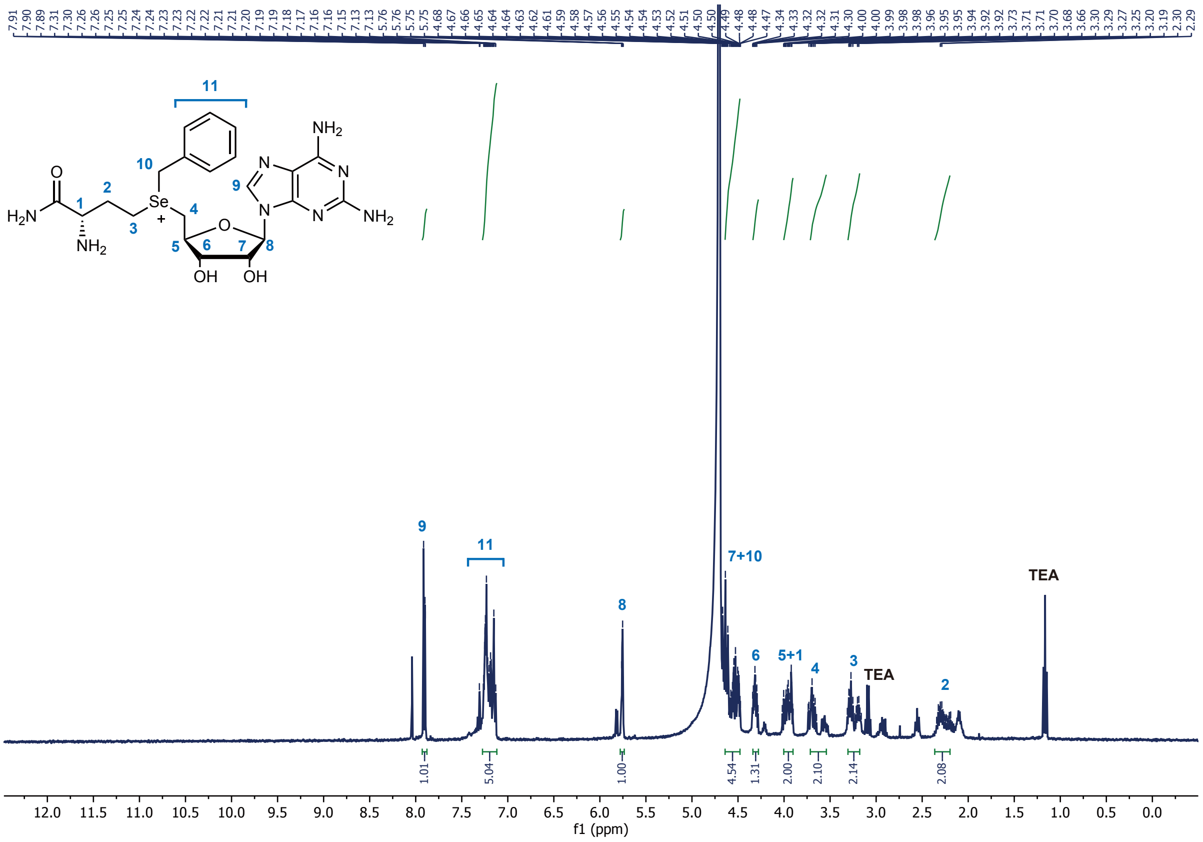

References

1 Okuda, T., Lenz, A. K., Seitz, F., Vogel, J. & Höbartner, C. A SAM analogue-utilizing ribozyme for site-specific RNA alkylation in living cells. *Nat Chem* **15**, 1523-1531 (2023)

2 Hirschmann, M., Zunino, R., Meninno, S., Falivene, L. & Fuoco, T. Bi-functional and mono-component organocatalysts for the ring-opening alternating co-polymerisation of anhydride and epoxide. *Catalysis Science & Technology* **13**, 7011-7021 (2023).

3 Duclos, R. I., Jr., Cleary, D. C., Catcott, K. C. & Zhou, Z. S. Synthesis and characterization of Se-adenosyl-L-selenohomocysteine selenoxide. *J Sulphur Chem* **36**, 135-144 (2015).

4 McKean, I. J. W. *et al.* S-Adenosyl Methionine Cofactor Modifications Enhance the Biocatalytic Repertoire of Small Molecule C-Alkylation. *Angew Chem Int Ed Engl* **58**, 17583-17588 (2019).

5 Yeo, W. L. *et al.* Probing the molecular determinants of fluorinase specificity. *Chem Commun (Camb)* **53**, 2559-2562 (2017).

6 Shah, R. *et al.* Design, Synthesis, and Characterization of Sulfamide and Sulfamate Nucleotidomimetic Inhibitors of hHint1. *ACS Med Chem Lett* **7**, 780-784 (2016).
