# Supplementary material for: Structure and catalytic activity of the SAM-utilizing ribozyme SAMURI
Source: Nat Chem Biol. 2025 Jan 8;22(6):938–47. doi: 10.1038/s41589-024-01808-w (PMC13226088; doi:10.1038/s41589-024-01808-w)
Supplement: Supplementary file 2 — Reporting Summary [file 41589_2024_1808_MOESM2_ESM.pdf]

nature portfolio

Corresponding author(s): Claudia Höbartner

Last updated by author(s): Oct 18, 2024

Reporting Summary

Nature Portfolio wishes to improve the reproducibility of the work that we publish. This form provides structure for consistency and transparency in reporting. For further information on Nature Portfolio policies, see our Editorial Policies and the Editorial Policy Checklist.

Statistics

For all statistical analyses, confirm that the following items are present in the figure legend, table legend, main text, or Methods section.

n/a	Confirmed	
☐	☒	The exact sample size (*n*) for each experimental group/condition, given as a discrete number and unit of measurement
☒	☐	A statement on whether measurements were taken from distinct samples or whether the same sample was measured repeatedly
☒	☐	The statistical test(s) used AND whether they are one- or two-sided<br>*Only common tests should be described solely by name; describe more complex techniques in the Methods section.*
☒	☐	A description of all covariates tested
☒	☐	A description of any assumptions or corrections, such as tests of normality and adjustment for multiple comparisons
☐	☒	A full description of the statistical parameters including central tendency (e.g. means) or other basic estimates (e.g. regression coefficient) AND variation (e.g. standard deviation) or associated estimates of uncertainty (e.g. confidence intervals)
☒	☐	For null hypothesis testing, the test statistic (e.g. *F*, *t*, *r*) with confidence intervals, effect sizes, degrees of freedom and *P* value noted<br>*Give P values as exact values whenever suitable.*
☒	☐	For Bayesian analysis, information on the choice of priors and Markov chain Monte Carlo settings
☒	☐	For hierarchical and complex designs, identification of the appropriate level for tests and full reporting of outcomes
☒	☐	Estimates of effect sizes (e.g. Cohen's *d*, Pearson's *r*), indicating how they were calculated

Our web collection on statistics for biologists contains articles on many of the points above.

Software and code

Policy information about availability of computer code

Data collection	diffraction data collection on EIGER M16 and EIGER2 M16 detectors (at P11 and P13 at DESY Hamburg), XDS, Typhoon PhosphorImager, BioRad Chemidoc
Data analysis	CCP4-7.0 package (inlcuding AIMLESS, POINTLESS, CTRUNCATE), PHENIX 1.20 (including autosol, phaser, eLBOW), COOT 0.9.4, Pymol 4.6.0., Origin 2019.

For manuscripts utilizing custom algorithms or software that are central to the research but not yet described in published literature, software must be made available to editors and reviewers. We strongly encourage code deposition in a community repository (e.g. GitHub). See the Nature Portfolio guidelines for submitting code & software for further information.

Data

Policy information about availability of data

All manuscripts must include a data availability statement. This statement should provide the following information, where applicable:

- Accession codes, unique identifiers, or web links for publicly available datasets
- A description of any restrictions on data availability
- For clinical datasets or third party data, please ensure that the statement adheres to our policy

Structural data obtained by X-ray crystallography were deposited in the Protein Data Bank (PDB); accession codes 9FN2, 9FN3, all other data are provided in the

Figures 1-4, Extended Data Figures 1-7, and Supplementary Information. Source gel data are available with the manuscript. other pdb codes used in the manuscript: 2GIS, 2QWY, 3E5C, 6YL5 taken from PDB (protein data bank).

Human research participants

Policy information about studies involving human research participants and Sex and Gender in Research.

Reporting on sex and gender	N/A
Population characteristics	N/A
Recruitment	N/A
Ethics oversight	N/A

Note that full information on the approval of the study protocol must also be provided in the manuscript.

Field-specific reporting

Please select the one below that is the best fit for your research. If you are not sure, read the appropriate sections before making your selection.

☒ Life sciences ☐ Behavioural & social sciences ☐ Ecological, evolutionary & environmental sciences

For a reference copy of the document with all sections, see nature.com/documents/nr-reporting-summary-flat.pdf

Life sciences study design

All studies must disclose on these points even when the disclosure is negative.

Sample size	no statistical methods were used to predetermine sample size; number of samples were based on experimental feasibility, with at least duplication, usually three independent experiments. Sample size of 2 or 3 is sufficient for replication of gel experiments when these show comparable results.
Data exclusions	no data were excluded from the analysis
Replication	experiments were replicated two, three or four times, and all replication attempts were successful
Randomization	samples were not allocated into experimental groups as this is not relevant for the study
Blinding	Investigators were not blinded during data acquisition and analysis as this is not feasible for experiments involving mult-step organic synthesis, and not commonly done for crystallization, kinetic and structure probing.

Reporting for specific materials, systems and methods

We require information from authors about some types of materials, experimental systems and methods used in many studies. Here, indicate whether each material, system or method listed is relevant to your study. If you are not sure if a list item applies to your research, read the appropriate section before selecting a response.

Materials & experimental systems

n/a	Involved in the study	
☒	☐	Antibodies
☒	☐	Eukaryotic cell lines
☒	☐	Palaeontology and archaeology
☒	☐	Animals and other organisms
☒	☐	Clinical data
☒	☐	Dual use research of concern

Methods

n/a	Involved in the study	
☒	☐	ChIP-seq
☒	☐	Flow cytometry
☒	☐	MRI-based neuroimaging
