# Supplementary figures and images for: Structure and catalytic activity of the SAM-utilizing ribozyme SAMURI
Source: Nat Chem Biol. 2025 Jan 8;22(6):938–47. doi: 10.1038/s41589-024-01808-w (PMC13226088; doi:10.1038/s41589-024-01808-w)

Fig. 1

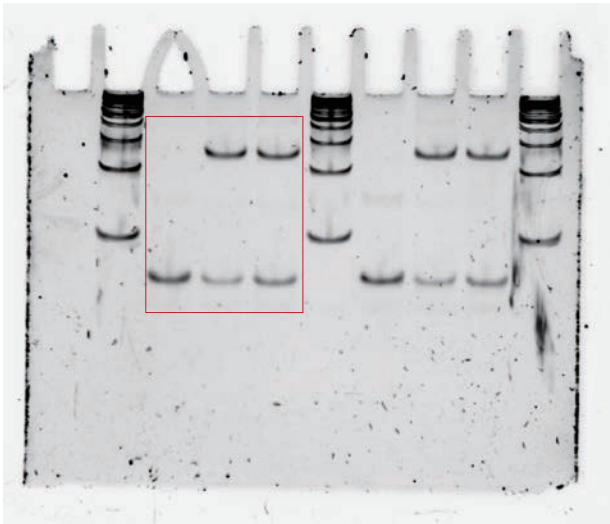

10 % nativePAGE, 10x8 cm, 200 V

Supplement: Supplementary file 3 — Unprocessed gel. [file 41589_2024_1808_MOESM3_ESM.pdf]

Fig. 2h

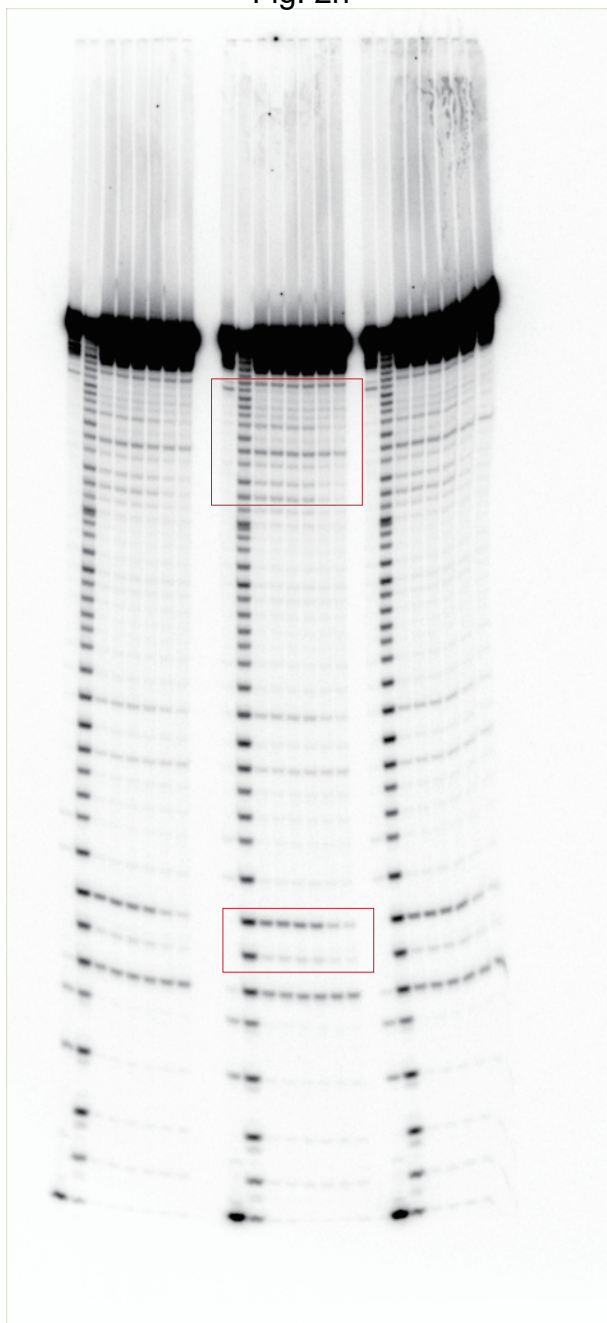

20 % dPAGE, 20×40 cm, 45W

Fig. 2i

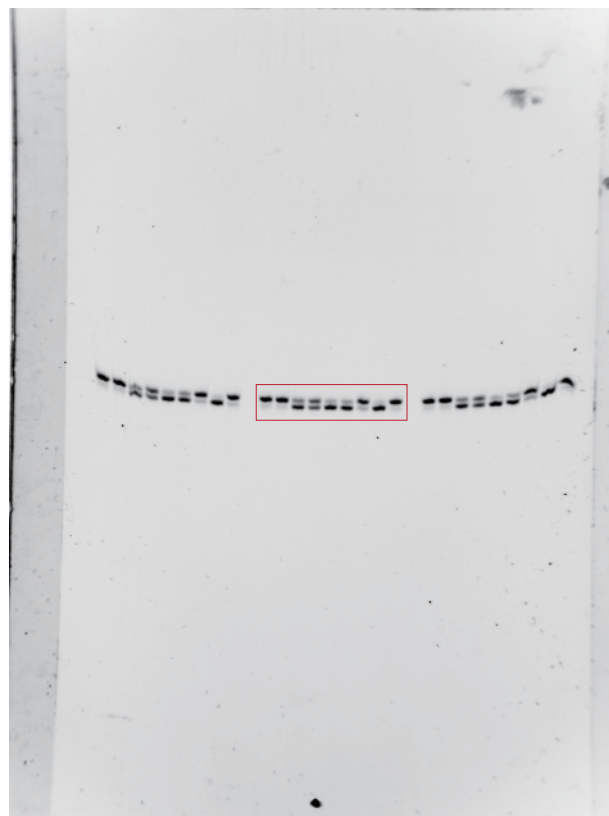

20 % dPAGE, 20×30 cm, 35W

Supplement: Supplementary file 4 — Unprocessed gels. [file 41589_2024_1808_MOESM4_ESM.pdf]

Fig. 4e

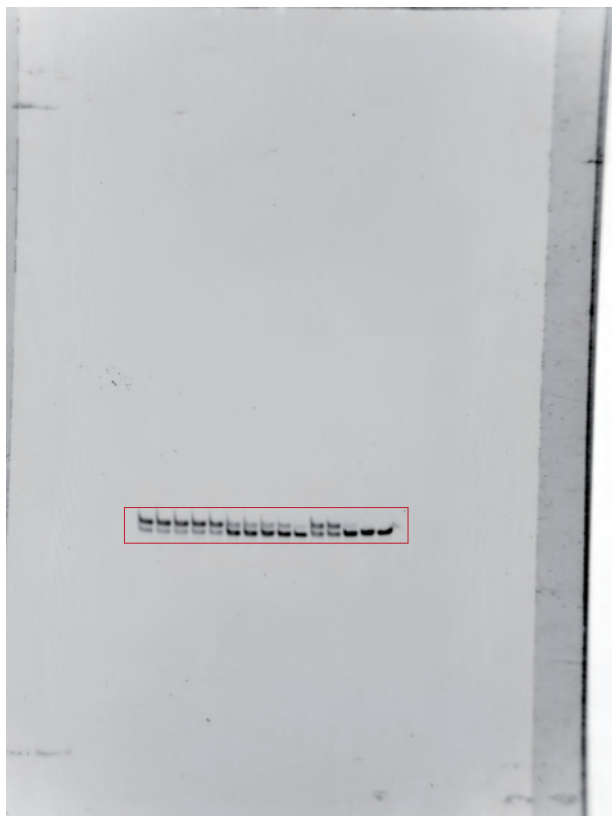

20 % dPAGE, 20x30 cm, 35W

Supplement: Supplementary file 5 — Unprocessed gel. [file 41589_2024_1808_MOESM5_ESM.pdf]

Fig. ED3 a

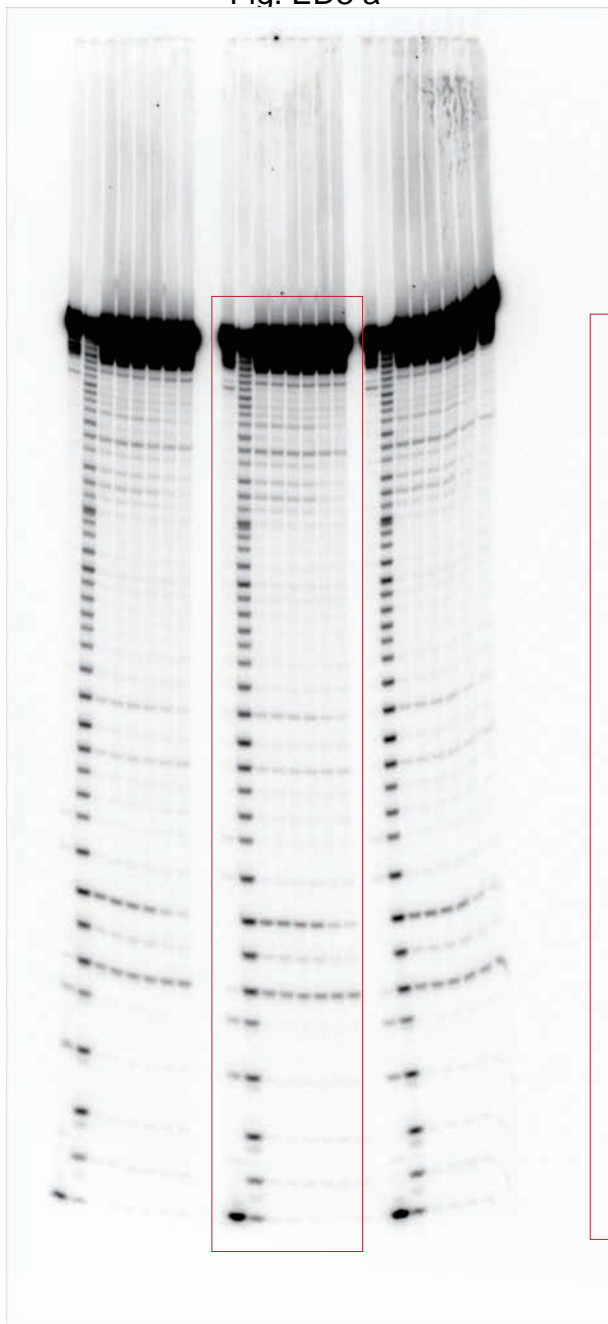

Fig. ED3 b

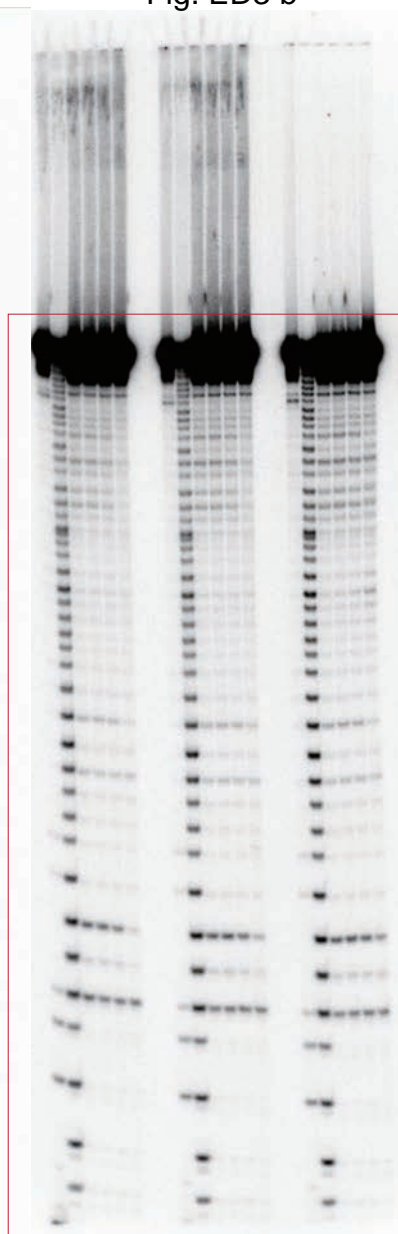

Fig. ED3 b 2nd

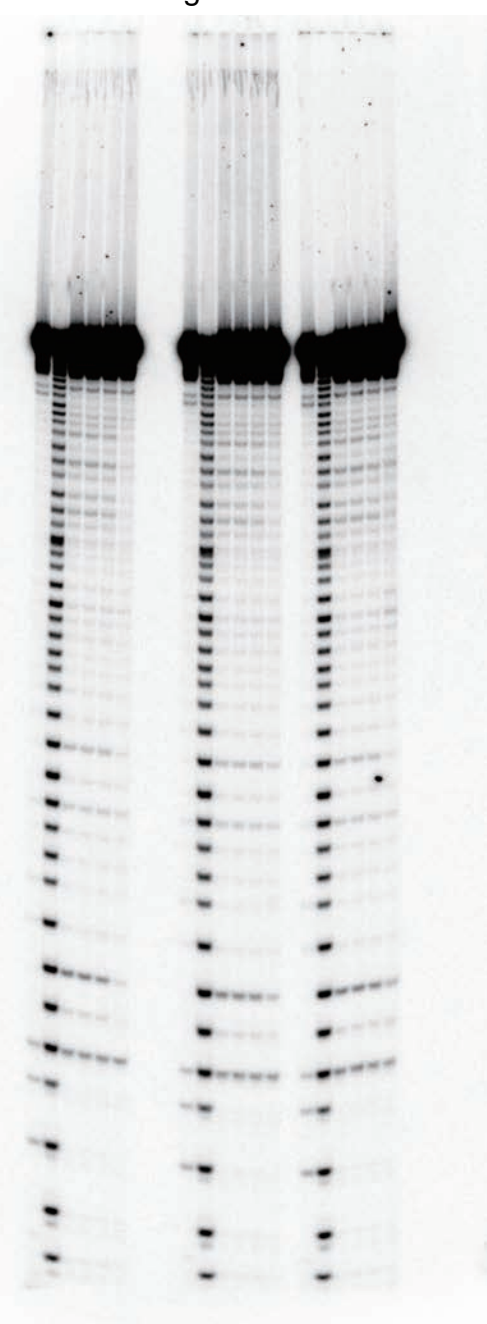

20 % dPAGE, 20x40 cm, 45W

20 % dPAGE, 20x40 cm, 45W

20 % dPAGE, 20x40 cm, 45W

Supplement: Supplementary file 7 — Unprocessed gels. [file 41589_2024_1808_MOESM7_ESM.pdf]

Fig. ED6a

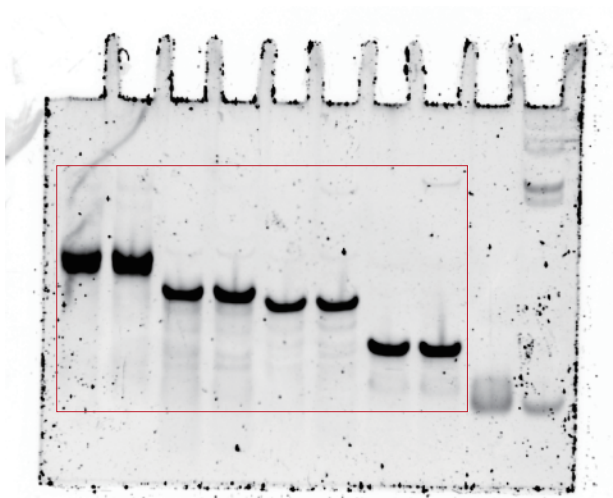

10 % nativePAGE, 10×8 cm, 200 V

Fig. ED6b

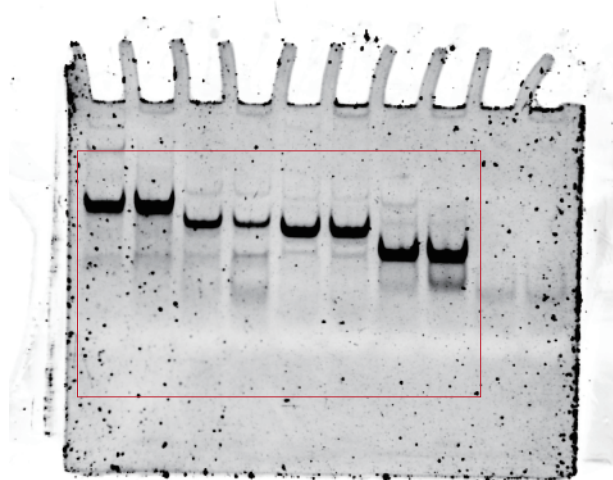

10 % nativePAGE, 10×8 cm, 200 V

Fig. ED6c

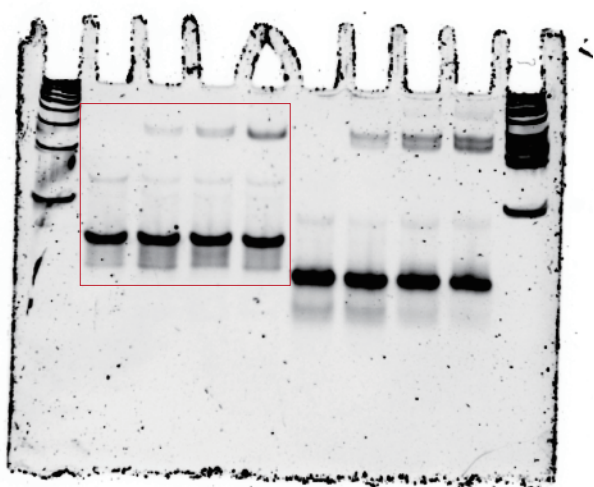

10 % nativePAGE, 10×8 cm, 200 V

Supplement: Supplementary file 11 — Unprocessed gels. [file 41589_2024_1808_MOESM11_ESM.pdf]
