# Supplementary material for: Structure and catalytic activity of the SAM-utilizing ribozyme SAMURI
Source: Nat Chem Biol. 2025 Jan 8;22(6):938–47. doi: 10.1038/s41589-024-01808-w (PMC13226088; doi:10.1038/s41589-024-01808-w)
Supplement: Supplementary file 8 — Unprocessed gels. [file 41589_2024_1808_MOESM8_ESM.pdf]

Fig. ED4 R5+R7 1st 2nd

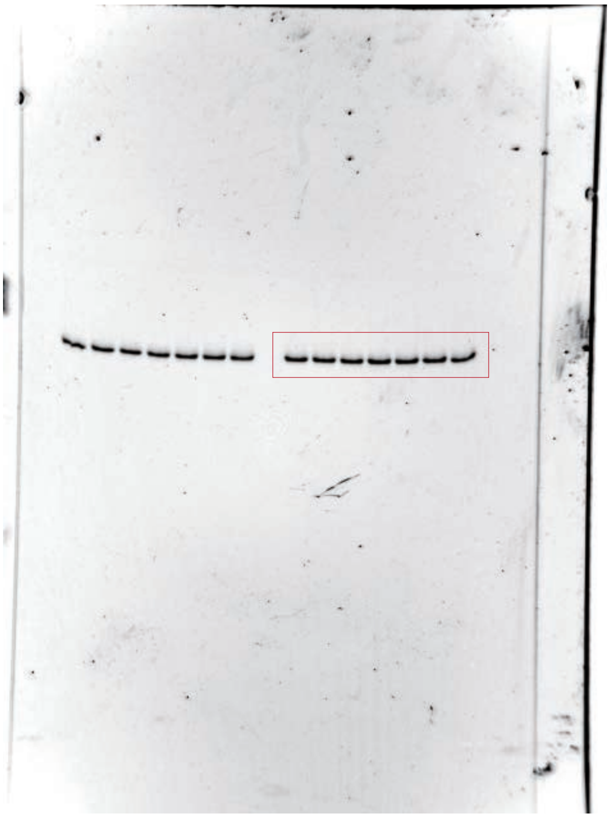

20 % dPAGE, 20×30 cm, 35W

Fig. ED4 R6+R7 1st 2nd

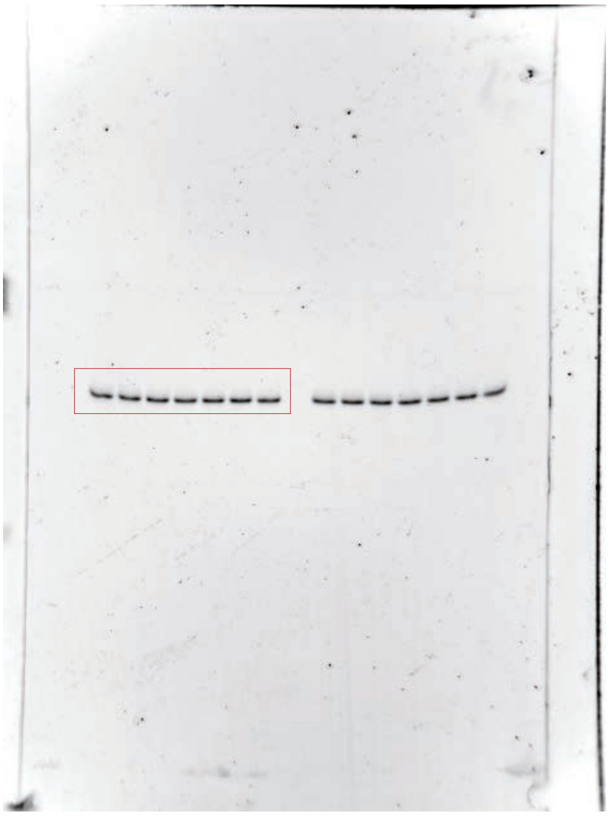

20 % dPAGE, 20×30 cm, 35W

Fig. ED4 R8 1st 2nd 10 µM

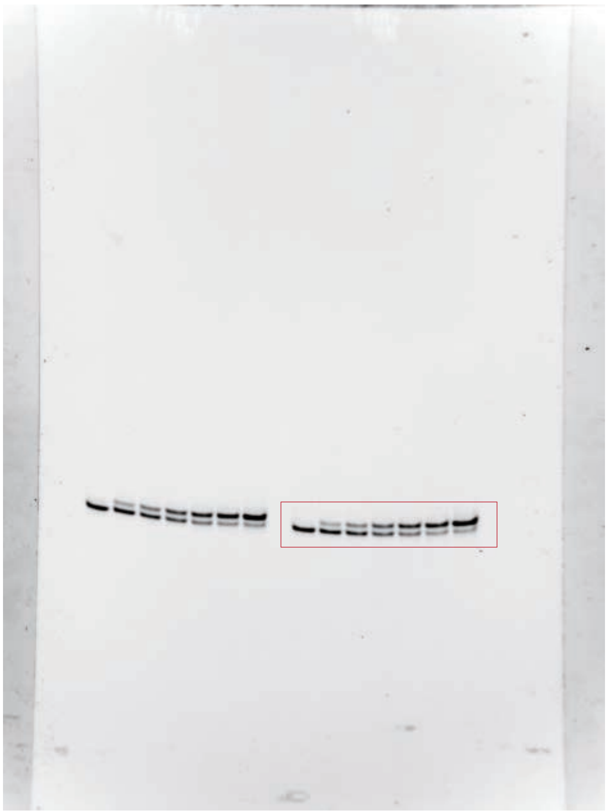

20 % dPAGE, 20×30 cm, 35W

Fig. ED4 R8 1st 2nd 50 µM

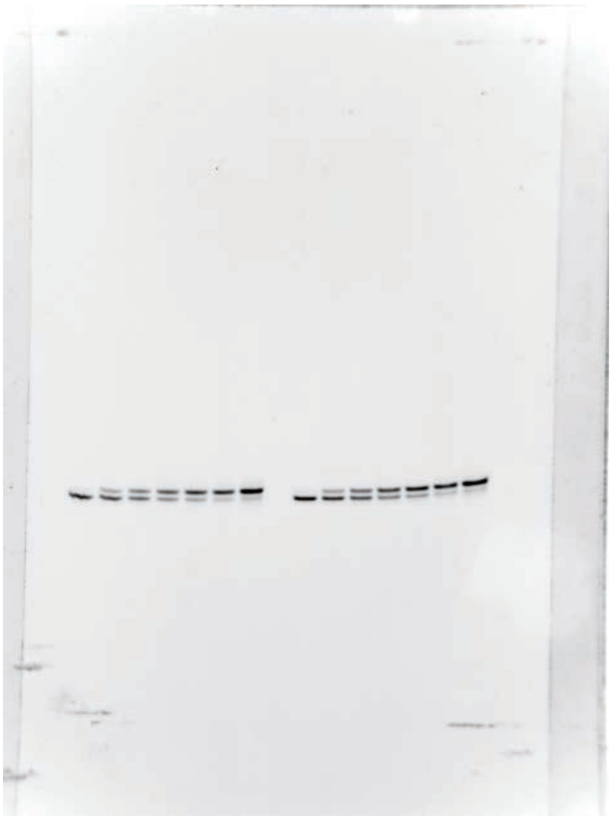

20 % dPAGE, 20×30 cm, 35W

Fig. ED4 R9 1st 2nd 10 µM

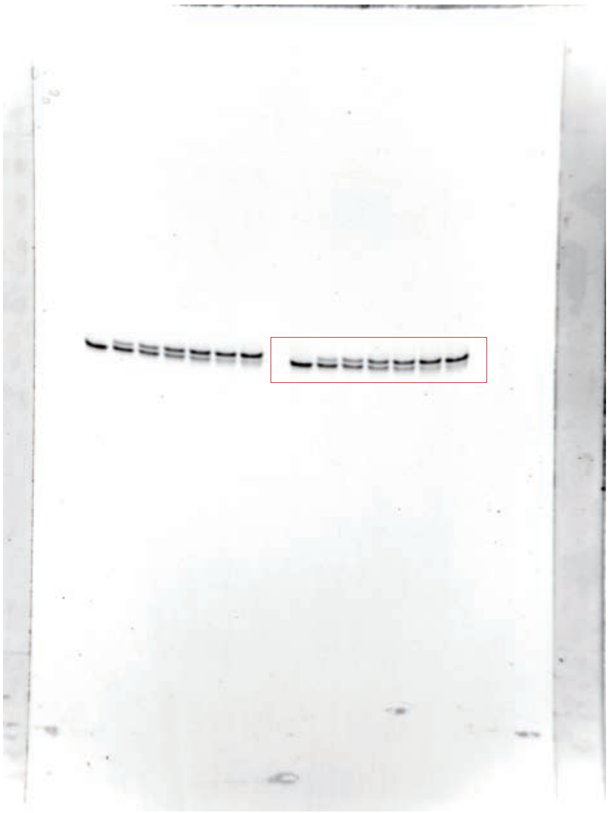

20 % dPAGE, 20×30 cm, 35W

Fig. ED4 R9 1st 2nd 50 µM

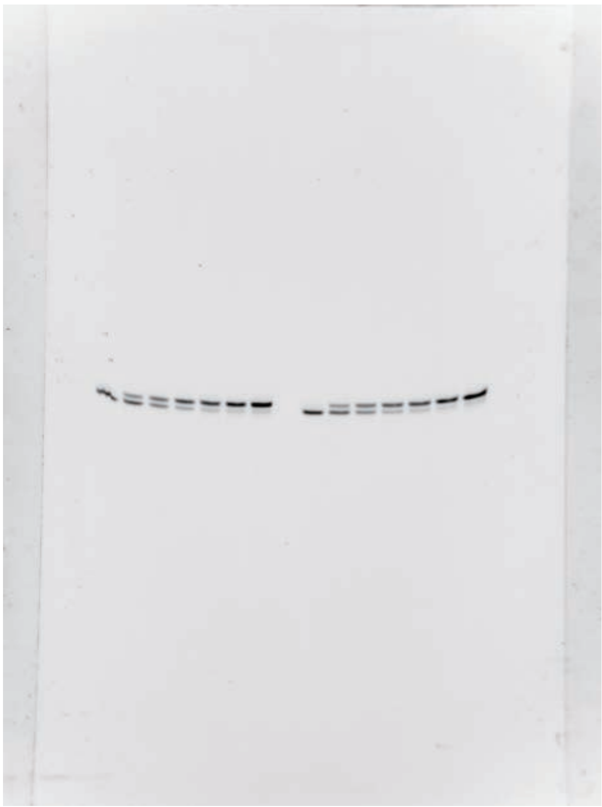

20 % dPAGE, 20×30 cm, 35W

Fig. ED4 R10 1st 10 µM

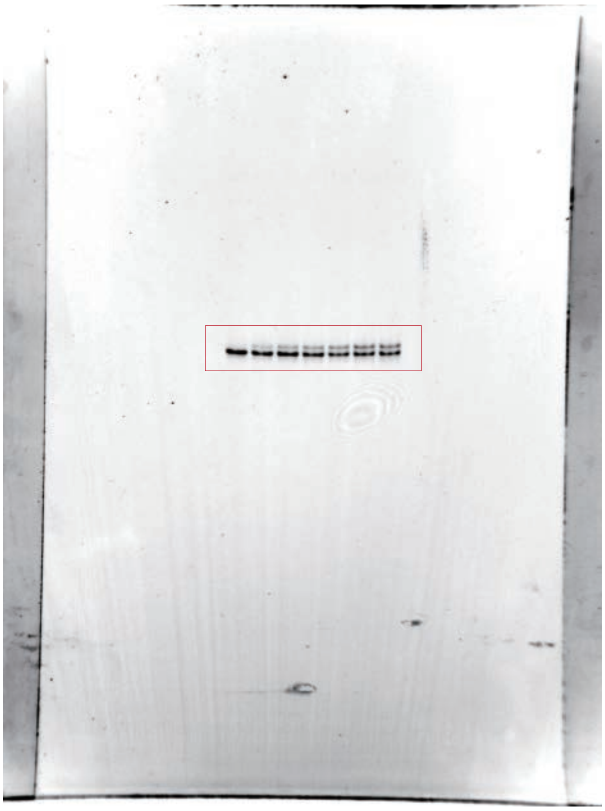

20 % dPAGE, 20×30 cm, 35W

Fig. ED4 R15, R10 2nd 10 µM

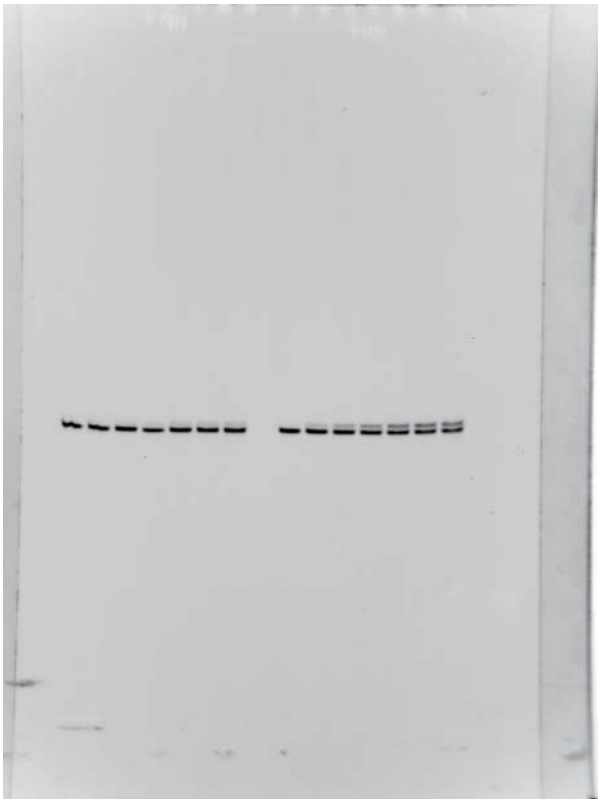

20 % dPAGE, 20×30 cm, 35W

Fig. ED4 R10 1st 2nd 50 µM

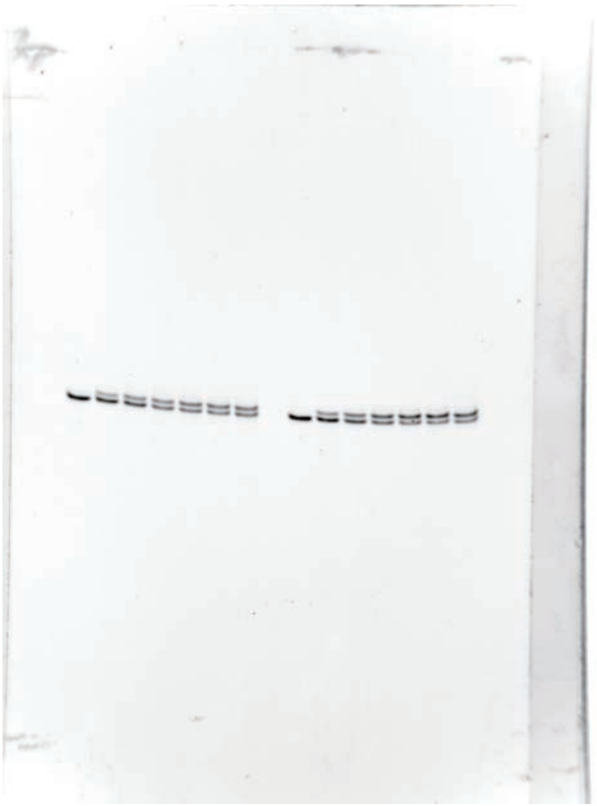

20 % dPAGE, 20×30 cm, 35W

Fig. ED4 R11, R12 1st 10µM

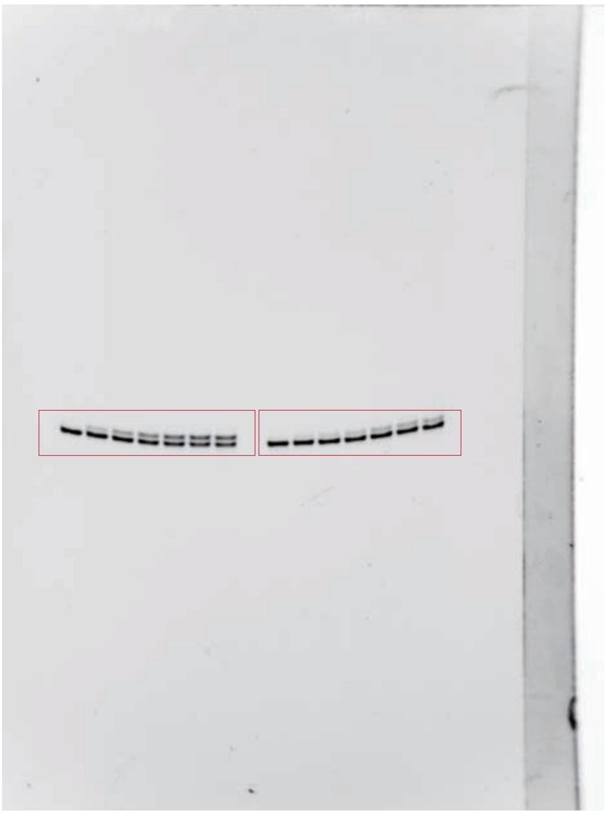

20 % dPAGE, 20×30 cm, 35W

Fig. ED4 R11, R12 2nd 10µM

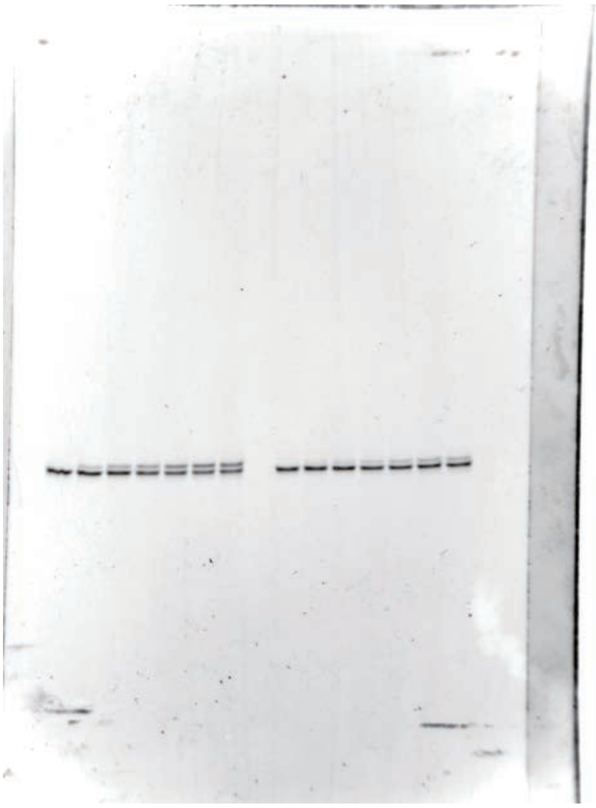

20 % dPAGE, 20×30 cm, 35W

Fig. ED4 R11 1st 2nd 50µM

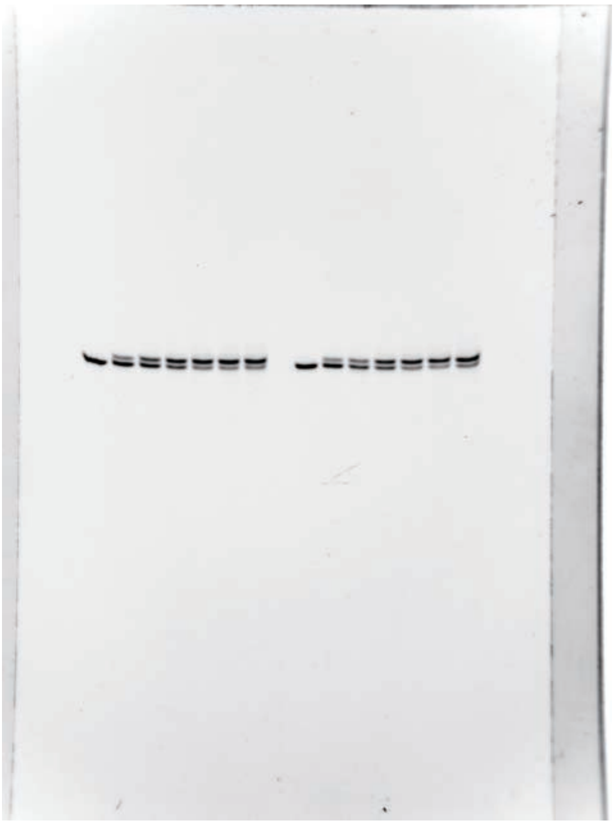

20 % dPAGE, 20×30 cm, 35W

Fig. ED4 R12 1st 2nd 50 µM

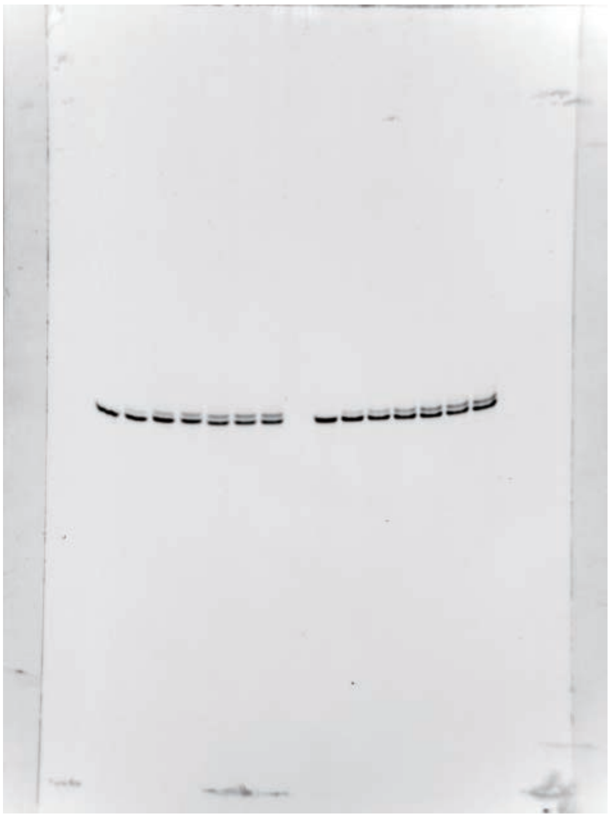

20 % dPAGE, 20×30 cm, 35W

Fig. ED4 R13, R14 1st 10µM

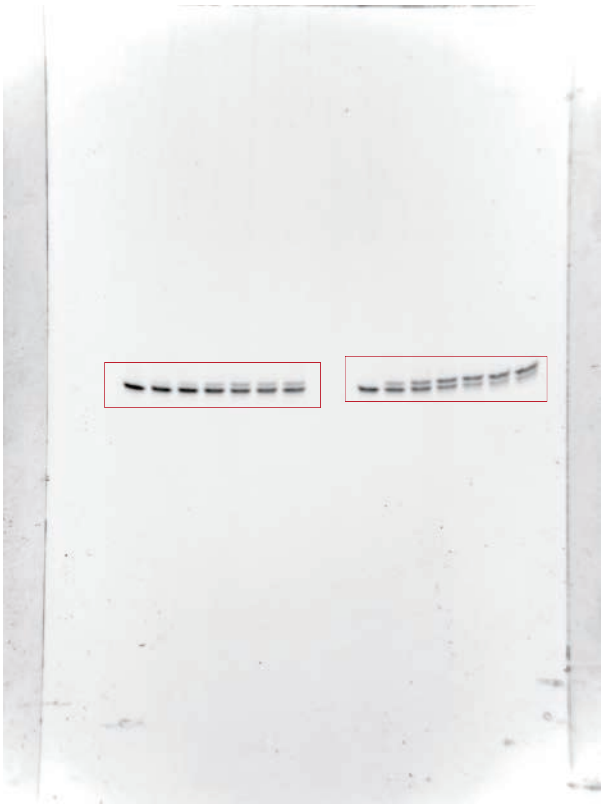

20 % dPAGE, 20×30 cm, 35W

Fig. ED4 R13, R14 2nd 10 µM

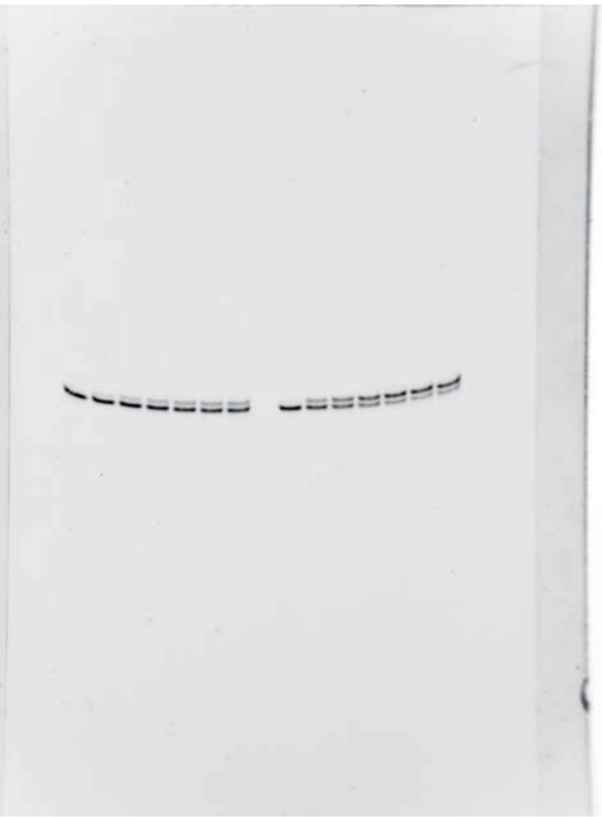

20 % dPAGE, 20×30 cm, 35W

Fig. ED4 R13 1st 2nd 50µM

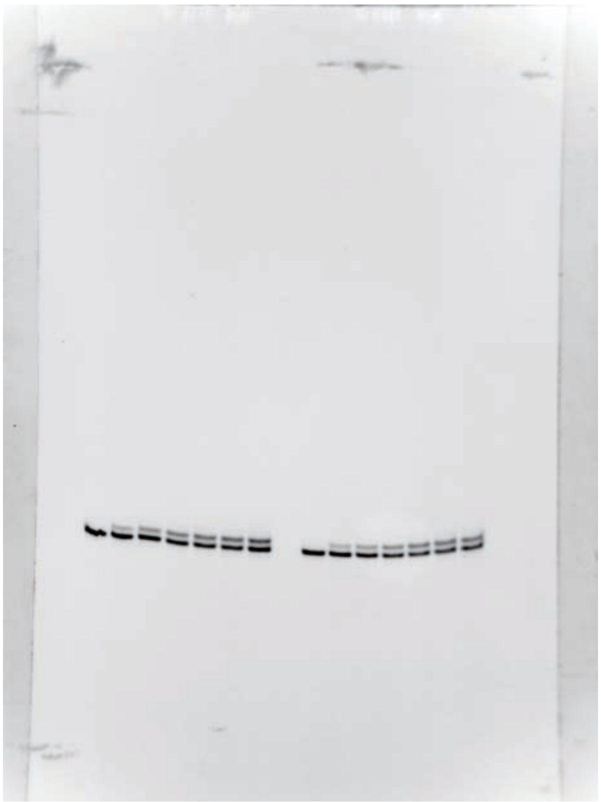

20 % dPAGE, 20×30 cm, 35W

Fig. ED4 R14 1st 2nd 50 µM

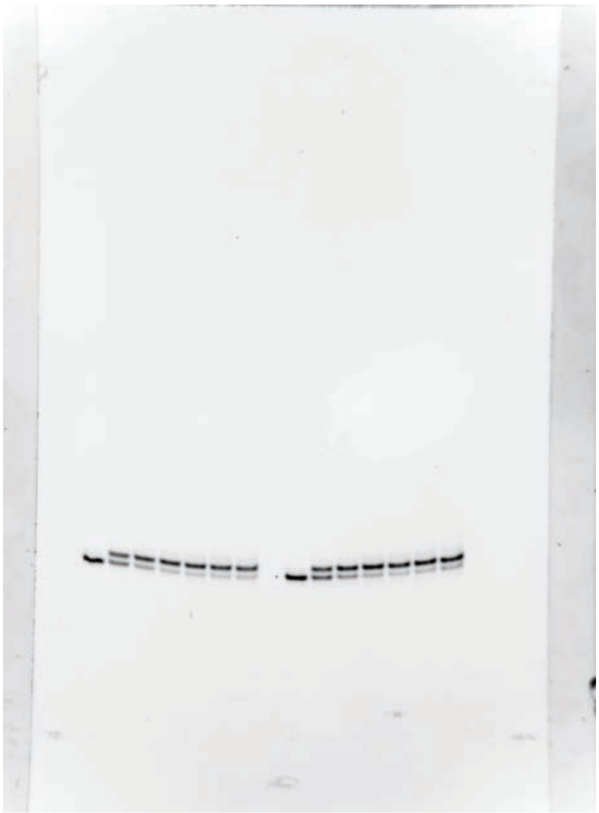

20 % dPAGE, 20×30 cm, 35W

Fig. ED4 R15 1st 10 µM

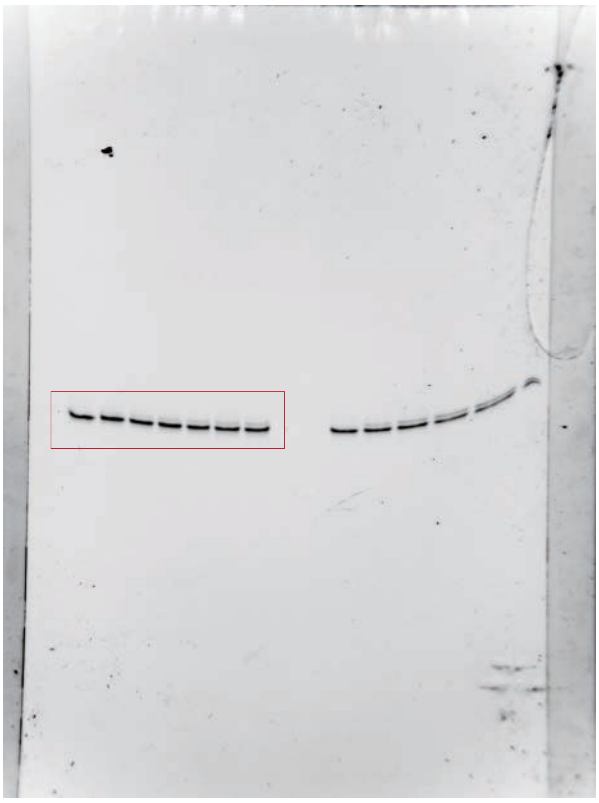

20 % dPAGE, 20×30 cm, 35W

Fig. ED4 R15 1st 2nd 50µM

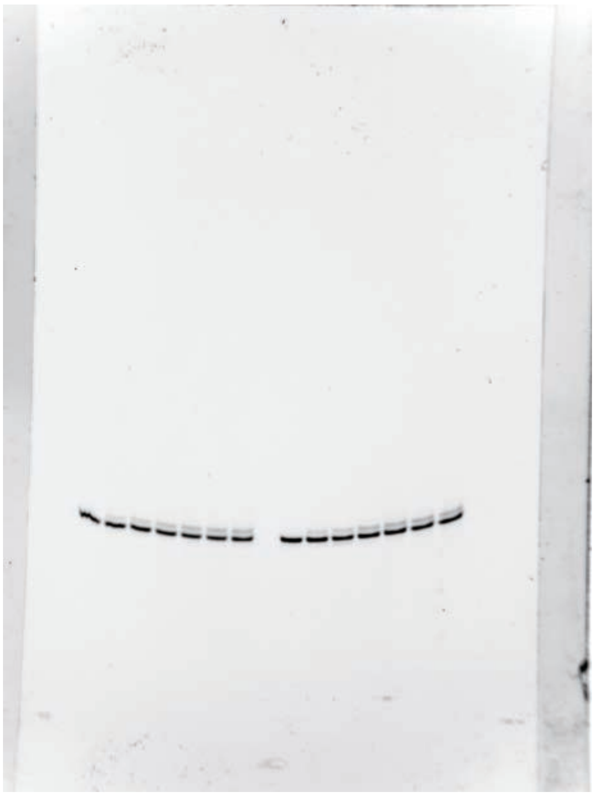

20 % dPAGE, 20×30 cm, 35W
