# Supplementary material for: Structure and catalytic activity of the SAM-utilizing ribozyme SAMURI
Source: Nat Chem Biol. 2025 Jan 8;22(6):938–47. doi: 10.1038/s41589-024-01808-w (PMC13226088; doi:10.1038/s41589-024-01808-w)
Supplement: Supplementary file 10 — Unprocessed gels. [file 41589_2024_1808_MOESM10_ESM.pdf]

Fig. ED5 ProSeDMA **1** (**S**)

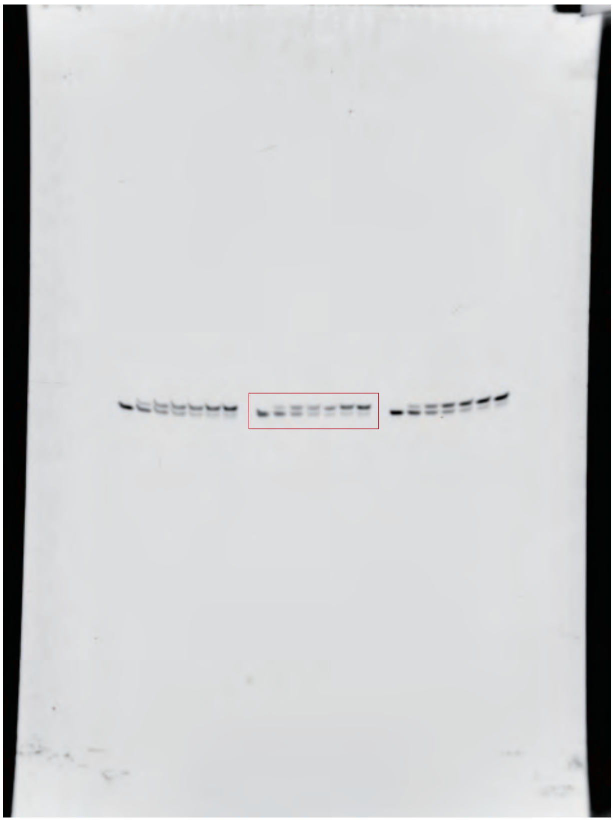

20 % dPAGE, 20×30 cm, 35W

Fig. ED5 ProSeDMA **1** (**R**)

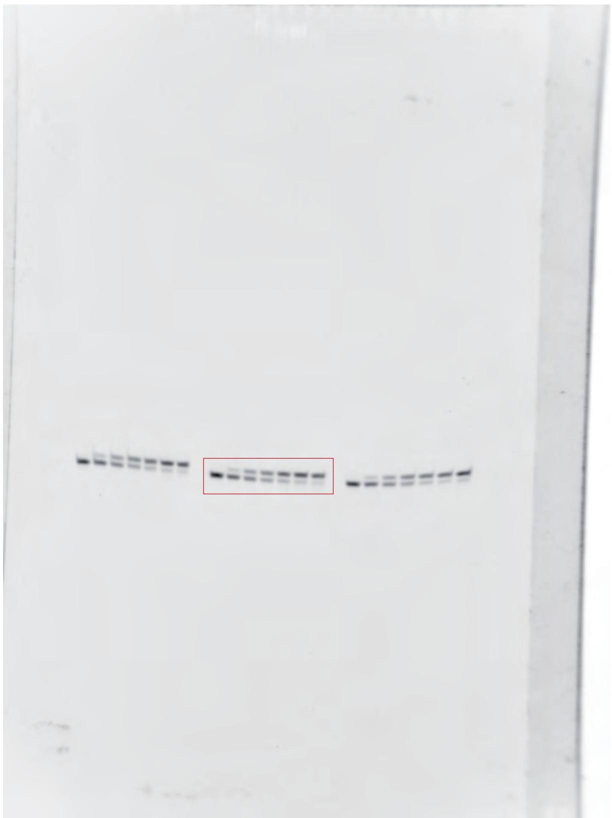

20 % dPAGE, 20×30 cm, 35W

Fig. ED5 ProSeDAB **5**

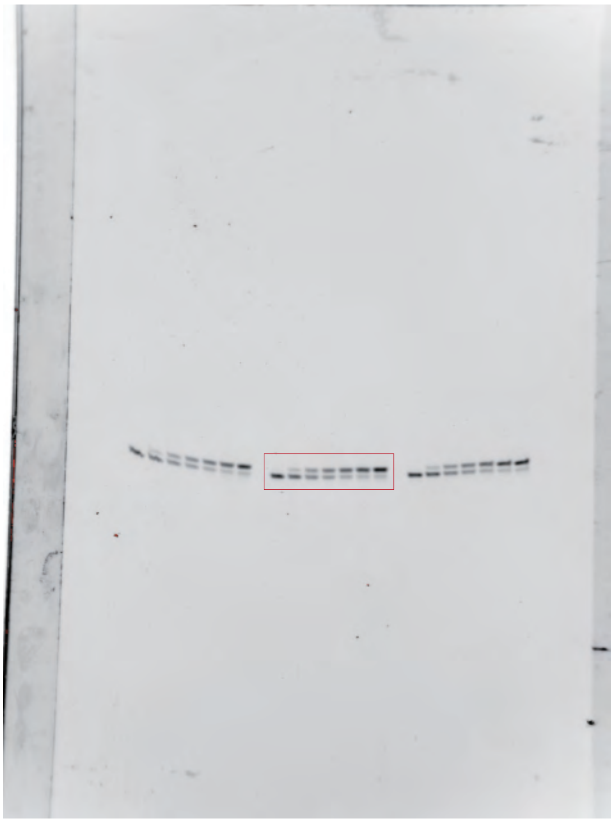

20 % dPAGE, 20×30 cm, 35W

Fig. ED5 ProSeDBA **7**

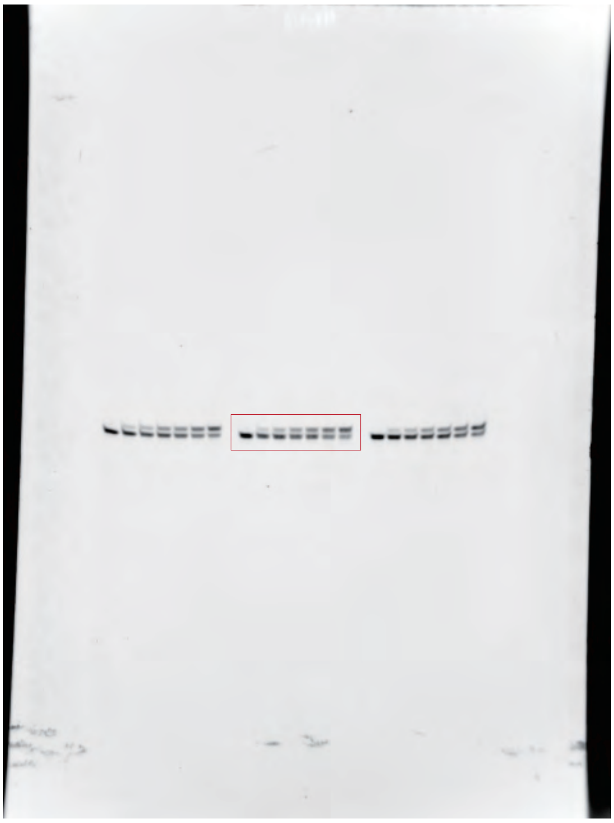

20 % dPAGE, 20×30 cm, 35W

Fig. ED5 ProSeDM **8** 9 min

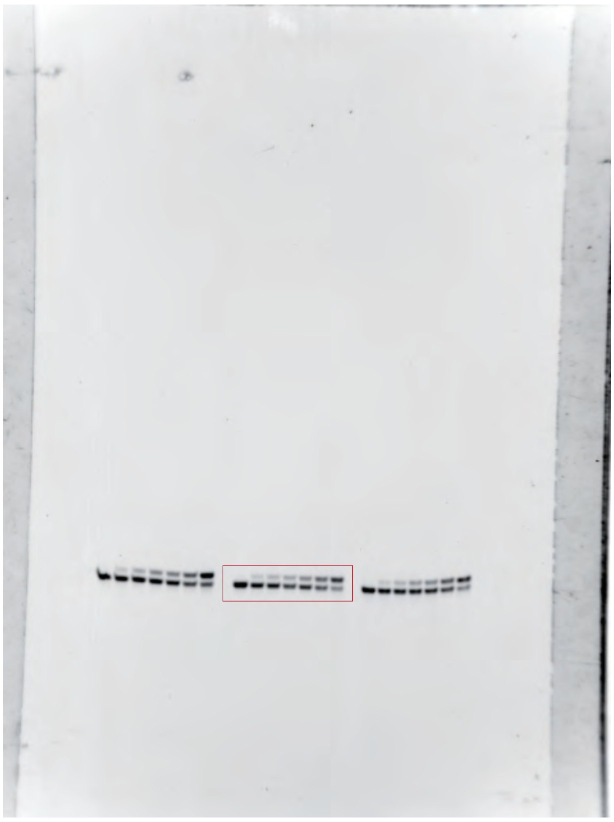

20 % dPAGE, 20×30 cm, 35W

Fig. ED5 ProSeDM **8** 10 min

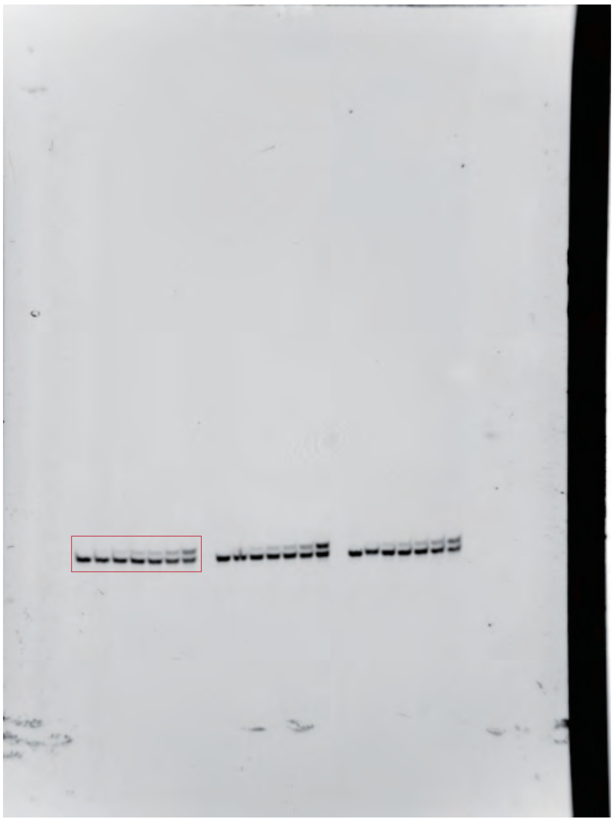

20 % dPAGE, 20×30 cm, 35W

Fig. ED5 ProSeAMA **9**

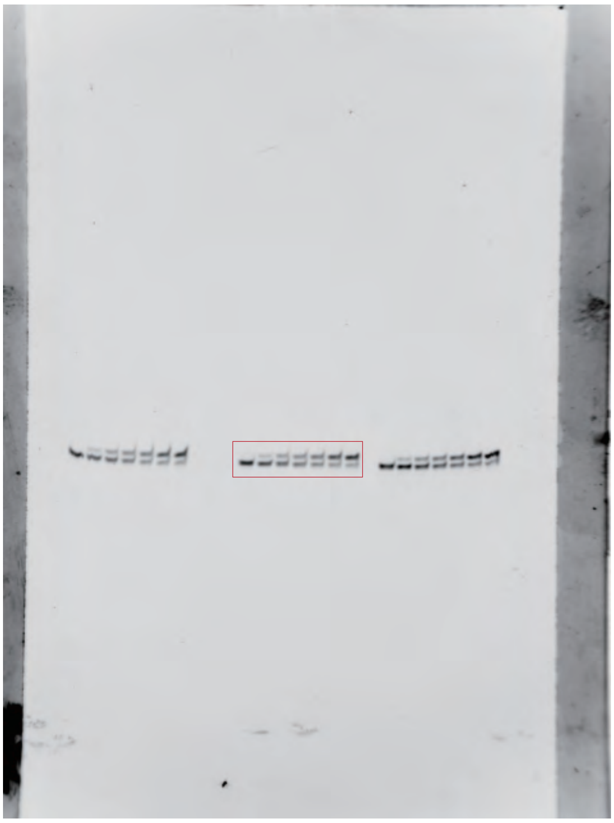

20 % dPAGE, 20×30 cm, 35W

Fig. ED5 ProSeAPMA **10**

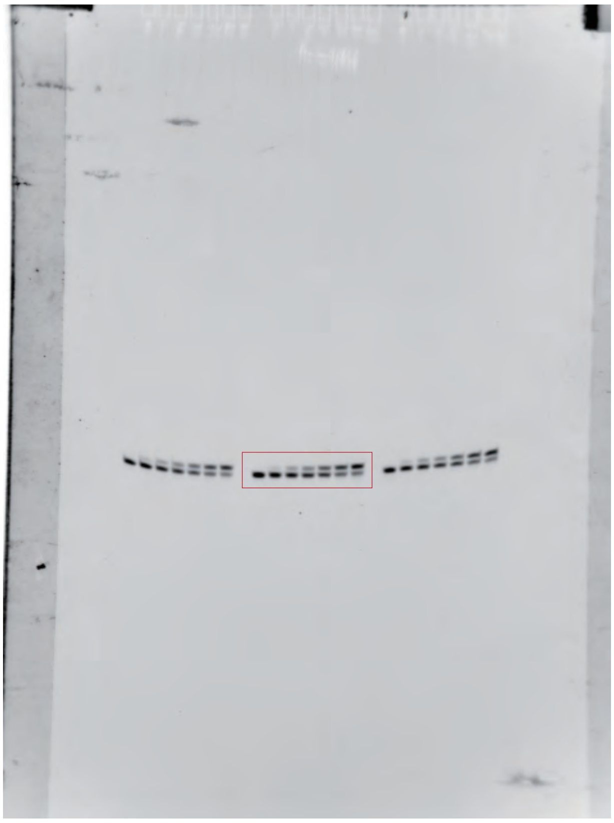

20 % dPAGE, 20×30 cm, 35W

Fig. ED5 ProSeNMA **11**

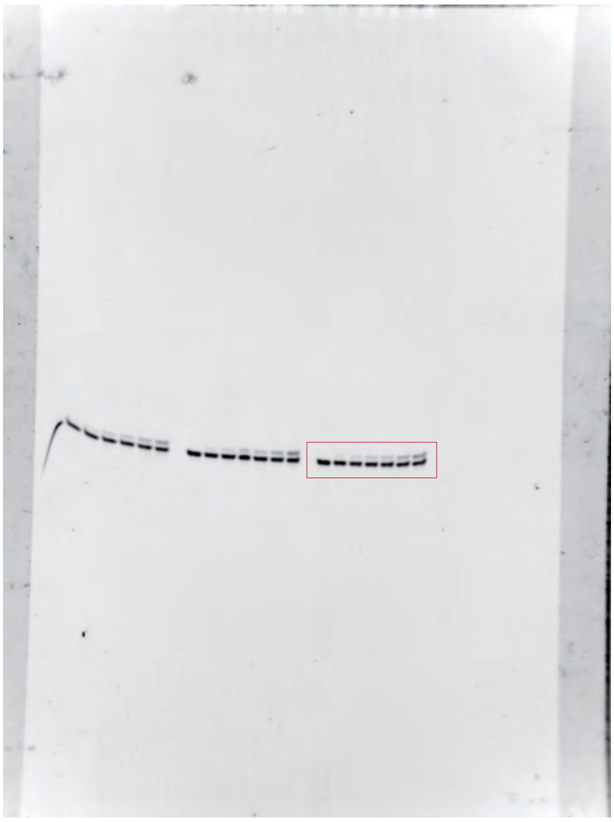

20 % dPAGE, 20×30 cm, 35W

Fig. ED5 ProSeGMA **12**

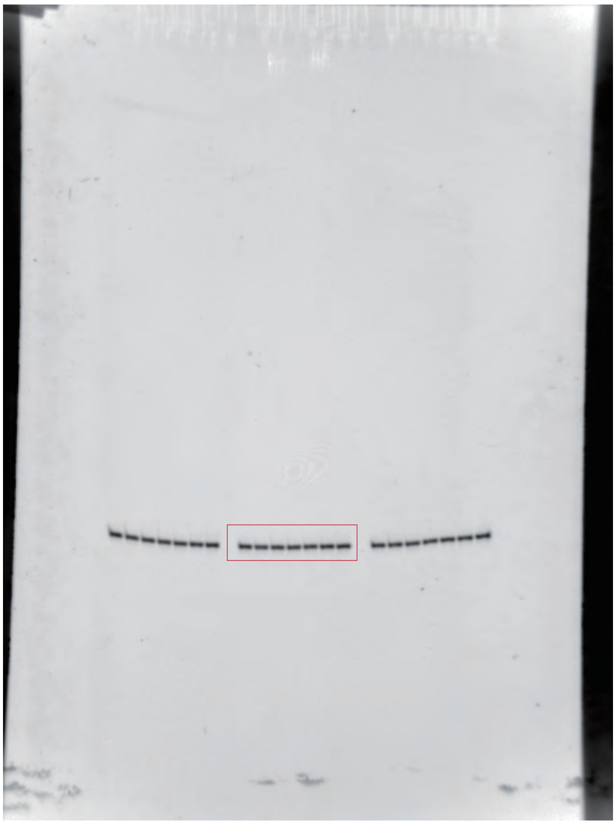

20 % dPAGE, 20×30 cm, 35W

Fig. ED5 ProSeMA **13**

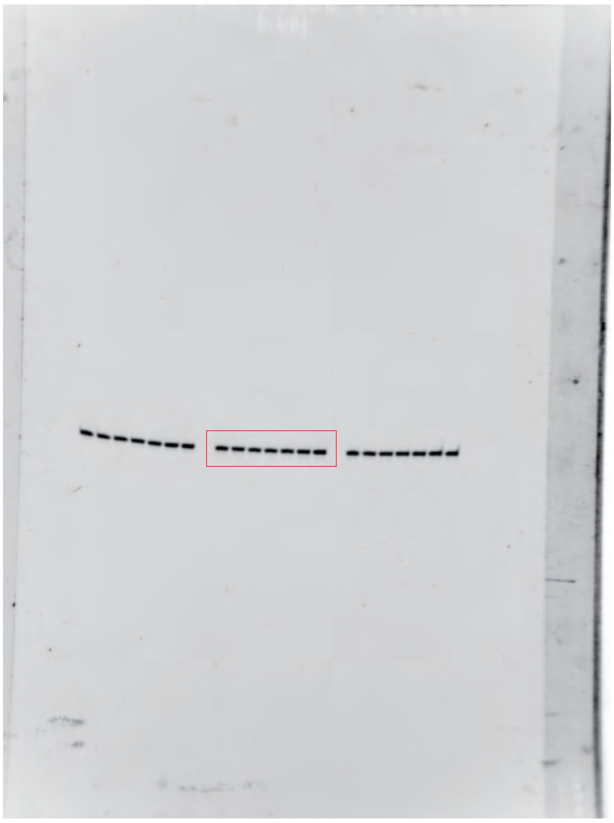

20 % dPAGE, 20×30 cm, 35W

Fig. ED5 BnSeDMA **37** 1st 2nd

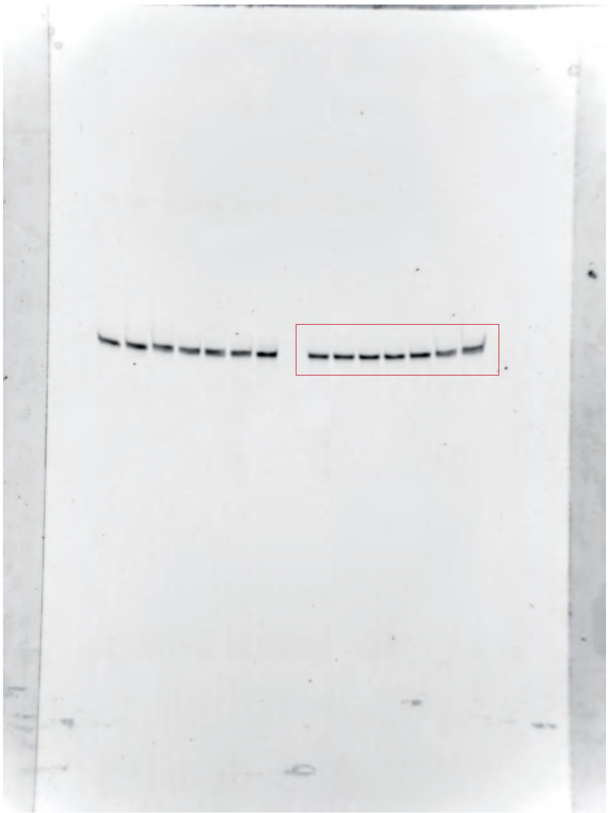

20 % dPAGE, 20×30 cm, 35W

Fig. ED5 BnSeDMA **37** 3rd

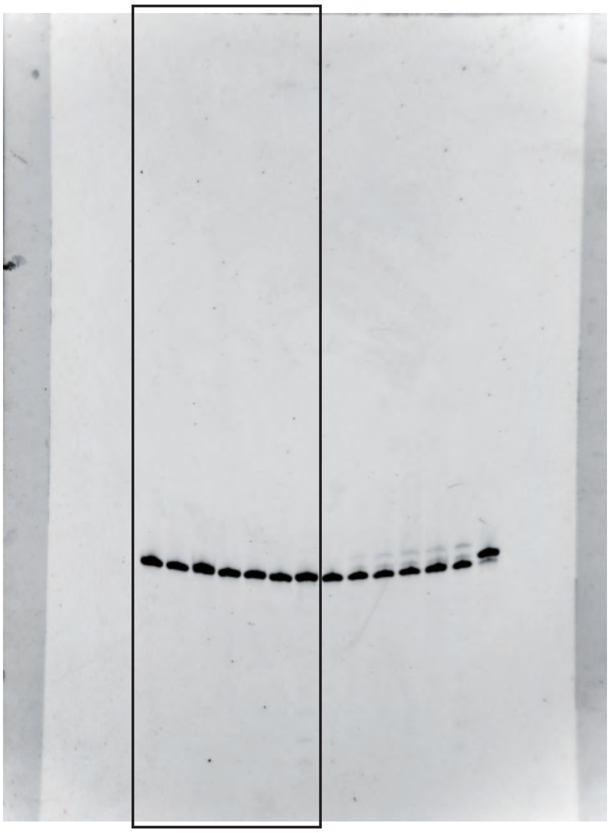

20 % dPAGE, 20×30 cm, 35W
